# Incompletely Observed Nonparametric Factorial Designs With Repeated Measurements: A Wild Bootstrap Approach

**DOI:** 10.1002/bimj.70008

**Published:** 2024-11-23

**Authors:** Lubna Amro, Frank Konietschke, Markus Pauly

**Affiliations:** ^1^ Department of Statistics TU Dortmund University Dortmund Germany; ^2^ Institute of Biometry and Clinical Epidemiology Charitè—Universitätsmedizin Berlin Berlin Germany; ^3^ Berlin Institute of Health (BIH) Berlin Germany; ^4^ UA Ruhr Research Center Trustworthy Data Science and Security Dortmund Germany

**Keywords:** missing values, nonparametric hypotheses, ordered categorical data, rank tests, repeated measures, wild bootstrap

## Abstract

In many life science experiments or medical studies, subjects are repeatedly observed and measurements are collected in factorial designs with multivariate data. The analysis of such multivariate data is typically based on multivariate analysis of variance (MANOVA) or mixed models, requiring complete data, and certain assumption on the underlying parametric distribution such as continuity or a specific covariance structure, for example, compound symmetry. However, these methods are usually not applicable when discrete data or even ordered categorical data are present. In such cases, nonparametric rank‐based methods that do not require stringent distributional assumptions are the preferred choice. However, in the multivariate case, most rank‐based approaches have only been developed for complete observations. It is the aim of this work to develop asymptotic correct procedures that are capable of handling missing values, allowing for singular covariance matrices and are applicable for ordinal or ordered categorical data. This is achieved by applying a wild bootstrap procedure in combination with quadratic form‐type test statistics. Beyond proving their asymptotic correctness, extensive simulation studies validate their applicability for small samples. Finally, two real data examples are analyzed.

## 1 Introduction

Factorial designs have a long history in several scientific fields such as biology, ecology, or medicine (Traux and Carkhuff 1965; Nesnow et al. 1998; Wildsmith et al. 2001; Biederman, Boutton, and Whisenant 2008; Lekberg et al. 2018). The reasons are simple: They are an efficient way to study main and interaction effects between different factors. Typically, such designs are inferred by parametric mean‐based procedures such as linear mixed models, or multivariate analysis of variance (MANOVA). These procedures, however, rely on restrictive distributional assumptions, such as multivariate normality, or special dependencies (Lawley 1938; Bartlett 1939; Davis 2002; Johnson and Wichern 2007; Fitzmaurice, Laird, and Ware 2012).

However, assessing multivariate normality or a specific type of covariance matrix is difficult in practice (Micceri 1989; Qian and Huang 2005; Bourlier et al. 2008; Xu and Cui 2008), especially when the sample sizes are small. In particular, Erceg‐Hurn and Mirosevich (2008) pointed out that “*Researchers relying on statistical tests (e.g., Levene's test) to identify assumption violations may frequently fail to detect deviations from normality and homoscedasticity that are large enough to seriously affect the type‐I error rate and power of classic parametric tests*” (p. 600). In fact, classical MANOVA procedures are usually nonrobust to deviations and may result in inaccurate decisions caused by possibly conservative or inflated type‐I error rates (Arnau et al. 2012; Pesarin and Salmaso 2012; Brombin, Midena, and Salmaso 2013; Konietschke et al. 2015; Pauly, Ellenberger, and Brunner 2015; Friedrich, Brunner, and Pauly 2017; Friedrich and Pauly 2018). Moreover, if ordinal, or ordered categorical data are present, mean‐based approaches are not applicable. A tempting alternative are nonparametric rank‐based methods, which are applicable for non‐normal data, in particular discrete data or even ordered categorical data. Their key features are their robustness and their invariance under monotone transformations of the data. Consequently, Akritas and Arnold (1994), Akritas and Brunner (1997), Munzel and Brunner (2000), Brunner and Puri (2001), Akritas (2011), Brunner et al. (2017), and Friedrich, Konietschke, and Pauly (2017) proposed nonparametric ranking methods for all kinds of factorial designs. Thereby, hypotheses are no longer formulated in terms of means (which do not exist for ordinal data), distribution functions are used instead. However, all these methods are only applicable for completely observed factorial designs data and cannot be used to analyze multivariate data with missing values.

In contrast, there are only a few methods that are applicable in case of missing values, require no parametric assumptions, and also lead to valid inferences in case of arbitrary covariance structures, skewed distributions, or unbalanced experimental designs. Examples in the nonparametric framework for the matched pairs designs include the ranking methods proposed by Akritas, Kuha, and Osgood (2002), Konietschke et al. (2012), and Fong et al. (2018). A promising approach for factorial designs with repeated measurements is given by the proposals of Brunner, Munzel, and Puri (1999) and Domhof, Brunner, and Osgood (2002) who recommended two types of quadratic forms for testing nonparametric hypotheses in terms of distribution functions: rank‐based Wald‐type statistic (WTS) and ANOVA‐type statistic (ATS). The Wald‐type test is an asymptotically valid test, which usually needs large sample sizes to obtain accurate test decisions, see Brunner (2001) and Friedrich, Konietschke, and Pauly (2017) for the case of complete observations and our simulation study below. Apart from that, the ANOVA‐type test is based on an approximation of its distribution with scaled $\chi^{2}$‐distributions. As the latter does not coincide with the ANOVA‐type test's limiting distribution under the null hypothesis $H_{0}$, the test is in general not asymptotically correct and also exhibits a more or less conservative behavior under small sample sizes, see Brunner (2001) for the case of complete observations. Another applicable technique is the nonparametric imputation method proposed by Gao (2007), which possesses a good type‐I error control but a lower power behavior than the methods by Brunner, Munzel, and Puri (1999) and Domhof, Brunner, and Osgood (2002), see the simulation study of Gao (2007). Additionally, recent works by Rubarth, Pauly, and Konietschke (2022) and Rubarth et al. (2022) introduce promising inference methods for testing null hypotheses formulated in terms of relative effects instead of distribution functions in factorial designs with missing data, without relying on parametric assumptions. Rubarth, Pauly, and Konietschke (2022) focus on nonparametric techniques for repeated measures designs, while Rubarth et al. (2022) extend this research to factorial designs with clustered data.

The aims of the present paper are (i) to provide statistical tests for hypotheses formulated in distribution functions that are capable of treating missing values in factorial designs; (ii) work without parametric assumptions such as continuity of the distribution functions, or nonsingular covariance matrices; (iii) are asymptotically correct while (iv) showing a satisfactorily type‐I error control and good power properties. To accomplish this, we propose three different quadratic‐form–type test statistics and equip them with a nonparametric Wild bootstrap procedure for calculating critical values to enhance their small sample performance. Here, we throughout assume missing completely at random (MCAR) mechanism, when constructing the test statistics and developing their related theories. However, in the simulation studies, we investigate the effects of some missing at random (MAR) scenarios on the test statistics performance under small sample sizes, see the Supporting Information for the explicit definition of the missing mechanisms.

The paper is organized as follows: First we introduce the statistical model and the hypotheses of interest in the next section. In Section 3, we introduce the test statistics and analyze their asymptotic behavior. In Section 4, the proposed wild bootstrap technique is explained. Section 5 displays the results from our extensive simulation study and two real data examples from a fluvoxamine study and a skin disorder clinical trial are analyzed in Section 6. All proofs as well as additional simulation results can be found in the Supporting Information.

To facilitate the presentation, we introduce the following notations: Let $I_{d}$ denote the *d*‐dimensional unit matrix, $J_{d}$ denote the d×d matrix of 1s that is, $J_{d}=1_{d}1_{d}^{T}$, where $1_{d}={\left(1,\text{…},1\right)}_{d\times 1}^{T}$ denotes the *d*‐dimensional column vector and $P_{d}=I_{d}-\frac{1}{d}J_{d}$ is the so‐called *d*‐dimensional centering matrix. Finally, by A⊗B we denote the Kronecker product of the matrices A and B.

## 2 Statistical Model and Nonparametric Hypotheses

We consider a nonparametric repeated measures model with a independent and potentially unbalanced treatment groups and d different time points given by independent random vectors

(2.1) $$\begin{array}{c} X_{ik}={\left(X_{i1k},\text{…},X_{idk}\right)}^{T},\;i=1,\text{…},a,\;k=1,\text{…},n_{i}, \end{array}$$

where $X_{ijk}∼F_{ij}\left(x\right)=\frac{1}{2}\left[F_{ij}^{+}\left(x\right)+F_{ij}^{-}\left(x\right)\right],i=1,\text{…},a,j=1,\text{…},d,k=1,\text{…},n_{i}$. Here, $F_{ij}^{+}\left(x\right)=P\left(X_{ijk}\leq x\right)$ is the right continuous version and $F_{ij}^{-}\left(x\right)=P\left(X_{ijk}<x\right)$ is the left continuous version of the distribution function. Using the normalized version $F_{ij}\left(x\right)$ is useful for handling ties and including continuous as well as discontinuous distribution functions (Brunner, Bathke, and Konietschke 2018). To include the case of missing values, we follow the notation of Brunner, Munzel, and Puri (1999) and let

(2.2) $$\begin{array}{ccc} \lambda_{ijk} & = & \left\{\begin{array}{c} 1,\;\text{if}\;X_{ijk}\;\text{is observed}\\ 0,\;\text{if}\;X_{ijk}\;\text{is nonobserved} \end{array}\right.\\ & & i=1,\text{…},a,j=1,\text{…},d,k=1,\text{…},n_{i}. \end{array}$$

Moreover, let $n=\sum_{i=1}^{a}n_{i}$ denote the total number of subjects and let

$N=\sum_{i=1}^{a}\sum_{j=1}^{d}\sum_{k=1}^{n_{i}}\lambda_{ijk}$ denote the total number of observations.

To formulate the null hypothesis in this nonparametric setup, let $F={\left(F_{11},\text{…},F_{ad}\right)}^{T}$ denote the vector of the distribution functions $F_{ij},i=1,\text{…},a,j=1,\text{…},d$. and C denote a contrast matrix, that is, C1=0 where $1={\left(1,\text{…},1\right)}^{T}$ and $0={\left(0,\text{…},0\right)}^{T}$. Then, the null hypotheses are formulated by $H_{0}:\left\{CF=0\right\}$. This framework covers different factorial repeated measures designs. For example, the hypothesis of no treatment group effect, that is, $H_{0}^{G}:\left\{{\bar{F}}_{1.}=\cdots ={\bar{F}}_{a.}\right\}$, is equivalently written in matrix notation as $H_{0}^{G}:\left\{\left(P_{a}⊗\frac{1}{d}1_{d}^{T}\right)F=0\right\}$. Similarly, the hypothesis of no time effect, that is, $H_{0}^{T}:\left\{{\bar{F}}_{.1}=\cdots ={\bar{F}}_{.d}\right\}$ is equivalently written as $H_{0}^{T}:\left\{\left(\frac{1}{a}1_{a}^{T}⊗P_{d}\right)F=0\right\}$, and the hypothesis of no interaction effect between treatment and time is written as $H_{0}^{GT}:\left\{\left(P_{a}⊗P_{d}\right)F=0\right\}$.

We note that more complex factorial structures on the repeated measures (e.g., in case of different interventions over time as in Sattler and Pauly 2018 or the groups (in case of two or more grouping factors) are also covered by our approach by simply splitting up the indices i (for a factorial group structure) or j (for a factorial time structure). For ease of presentation, we will focus on the above hypotheses.

To entail a parameter for describing differences between distributions, Brunner, Munzel, and Puri (1999) and Domhof, Brunner, and Osgood (2002) considered the relative marginal effects

(2.3) $$\begin{array}{c} p_{ij}=\int H\left(x\right)dF_{ij}\left(x\right), \end{array}$$

where $H\left(x\right)=N^{-1}\sum_{i=1}^{a}\sum_{j=1}^{d}\sum_{k=1}^{n_{i}}\lambda_{ijk}F_{ij}\left(x\right)$ is the weighted average of all distribution functions in the experiment. Estimators thereof are given by plugging‐in the empirical versions of $F_{ij}\left(x\right)$ and H(x)

(2.4) $$\begin{array}{cc} {\hat{F}}_{ij}\left(x\right) & =\frac{1}{\lambda_{ij.}}\sum_{k=1}^{n_{i}}\lambda_{ijk}{\hat{F}}_{ijk}\left(x\right) \end{array}$$

(2.5) $$\begin{array}{cc} & =\frac{1}{\lambda_{ij.}}\sum_{k=1}^{n_{i}}\lambda_{ijk}c\left(x-X_{ijk}\right),\;\lambda_{ij.}=\sum_{k=1}^{n_{i}}\lambda_{ijk}, \end{array}$$

(2.6) $$\begin{array}{cc} \hat{H}\left(x\right) & =\frac{1}{N}\sum_{i=1}^{a}\sum_{j=1}^{d}\sum_{k=1}^{n_{i}}c\left(x-X_{ijk}\right), \end{array}$$

where c(u) is the normalized version of the counting function, that is, c(u)=0,1/2 or 1 according as u<0,u=0 or u>0 and $c(x-X_{ijk})$ is assumed to equal 0 if the observation $X_{ijk}$ is missing. Note that ${\hat{F}}_{ij}\left(x\right)=0$ in case of $\lambda_{ij.}=0$. Thus, the relative marginal effects $p_{ij}$ are estimated by

(2.7) $$\begin{array}{cc} {\hat{p}}_{ij} & =\int \hat{H}d{\hat{F}}_{ij}=\frac{1}{\lambda_{ij.}}\sum_{k=1}^{n_{i}}\lambda_{ijk}\hat{H}\left(X_{ijk}\right) \end{array}$$

(2.8) $$\begin{array}{cc} & =\frac{1}{\lambda_{ij.}}\sum_{k=1}^{n_{i}}\lambda_{ijk}\frac{1}{N}\left(R_{ijk}-\frac{1}{2}\right), \end{array}$$

where $R_{ijk}$ is the mid‐rank of $X_{ijk}$ among all N dependent and independent observations. For convenience, the relative marginal effect estimators are collected in the vector $\hat{p}={\left({\hat{p}}_{11},\text{…},{\hat{p}}_{ad}\right)}^{T}$.

In the sequel, we derive asymptotic theory under the following sample size assumption and missing values:

Assumption 1 $\frac{\lambda_{ij.}}{n}\to \kappa_{i}\in \left[0,1\right]\;i=1,\text{…},a,\;j=1,\text{…},d$, as $n_{0}\equiv min\left\{\lambda_{ij.}\right\}\to \infty$.

This ensures that there are “enough” subjects without missing values in each group.

### 2.1 Asymptotic Distribution

Brunner, Munzel, and Puri (1999) derived the asymptotic distribution of $\sqrt{n}C\hat{p}$ under the null hypothesis $H_{0}:CF=0$. Setting ${\hat{Y}}_{ijk}=\hat{H}\left(X_{ijk}\right)$ and $Y_{ijk}=H\left(X_{ijk}\right)$, they proved that under $H_{0}$, $\sqrt{n}C\hat{p}$ and $\sqrt{n}C{\bar{Y}}_{.}$ are asymptotically equivalent. Accordingly, they showed that $\sqrt{n}C\hat{p}$ follows under $H_{0}$, asymptotically, a multivariate normal distribution with expectation 0 and covariance matrix $CV_{n}C^{T}$. Here

(2.9) $$\begin{array}{c} V_{n}=\overset{d}{\underset{i=1}{⨁}}\frac{n}{n_{i}}V_{i}=\overset{d}{\underset{i=1}{⨁}}\frac{n}{n_{i}^{2}}\sum_{k=1}^{n_{i}}\Lambda_{ik}V_{ik}\Lambda_{ik}, \end{array}$$

where $\Lambda_{ik}=n_{i}diag\left\{\frac{\lambda_{i1k}}{\lambda_{i1.}},\text{…},\frac{\lambda_{idk}}{\lambda_{id.}}\right\}$ and $V_{ik}=Cov\left(Y_{ik}\right)$ denotes the covariance matrix of the random vectors $Y_{ik}={\left(H\left(X_{i1k}\right),\text{…},H\left(X_{idk}\right)\right)}^{T}$.

Brunner, Munzel, and Puri (1999) propose to estimate the covariance matrix $V_{n}$ by

(2.10) $$\begin{array}{c} {\hat{V}}_{n}=\overset{r}{\underset{i=1}{⨁}}\frac{n}{n_{i}}{\hat{V}}_{i}, \end{array}$$

where ${\hat{V}}_{i}=\left[{\hat{v}}_{i}\left(j,j^{\prime }\right)\right]$ with diagonal and off‐diagonal elements ${\hat{v}}_{i}\left(j,j\right)$ and ${\hat{v}}_{i}\left(j,j^{\prime }\right)$, respectively, defined as

(2.11) $$\begin{array}{c} {\hat{v}}_{i}\left(j,j\right)=\frac{n_{i}\sum_{k=1}^{n_{i}}\lambda_{ijk}{\left[R_{ijk}-{\bar{R}}_{ij.}\right]}^{2}}{\left(N^{2}\right)\lambda_{ij.}\left(\lambda_{ij.}-1\right)}, \end{array}$$

(2.12) $$\begin{array}{c} {\hat{v}}_{i}\left(j,j^{\prime }\right)=\frac{n_{i}\sum_{k=1}^{n_{i}}\lambda_{ijk}\lambda_{ij^{\prime }k}\left[\left(R_{ijk}-{\bar{R}}_{ij.}\right)\left(R_{ij^{\prime }k}-{\bar{R}}_{ij^{\prime }.}\right)\right]}{\left(N^{2}\right)\left(\left(\lambda_{ij.}-1\right)\left(\lambda_{ij^{\prime }.}-1\right)+\Delta_{i,jj^{\prime }}-1\right)}. \end{array}$$

Here, ${\bar{R}}_{ij.}=\frac{1}{\lambda_{ij.}}\sum_{k=1}^{n_{i}}\lambda_{ijk}\left(R_{ijk}\right)$ and $\Delta_{i,jj^{\prime }}=\sum_{k=1}^{n_{i}}\lambda_{ijk}\lambda_{ij^{\prime }k}$. Under Assumption 1, Brunner, Munzel, and Puri (1999) have shown that ${\hat{V}}_{i}$ is a consistent estimator of $V_{i}$.

## 3 Statistics and Asymptotics

In this section, we propose three different quadratic forms for testing the null hypothesis: a WTS, an ATS as suggested in Brunner, Munzel, and Puri (1999) and Domhof, Brunner, and Osgood (2002) as well as a modified ANOVA‐type statistic (MATS). For mean‐based analyses, the latter was proposed by Friedrich and Pauly (2018).

The rank version of the WTS is defined as

(3.1) $$\begin{array}{c} T_{W}=n{\hat{p}}^{T}C^{T}{\left[C{\hat{V}}_{n}C^{T}\right]}^{+}C\hat{p}, \end{array}$$

where ${\left[B\right]}^{+}$ denotes the Moore‐penrose inverse of a matrix B. Its asymptotic null distribution is summarized below.

Theorem 3.1 Under Assumption (1) and $V_{n}\to V>0$ as $n_{0}\to \infty$, the statistic $T_{W}$ has under the null hypothesis $H_{0}:CF=0$, asymptotically, as $n_{0}\to \infty$, a central $\chi_{f}^{2}$‐distribution with f=rank(C) degrees of freedom.

WTSs of similar form are used in many different situations, for example, in heteroscedastic mean‐based analyses (Krishnamoorthy and Lu 2010; Xu et al. 2013; Konietschke et al. 2015; Friedrich and Pauly 2018; Amro, Pauly, and Ramosaj 2019) and even more complex regression models in survival analyses (Martinussen and Scheike 2007; Dobler, Pauly, and Scheike 2019). However, the convergence of the WTS to its limiting $\chi^{2}$‐distribution is usually slow and large sample sizes are required to obtain adequate results (Vallejo, Fernández, and Livacic‐Rojas 2010; Konietschke et al. 2015; Pauly, Brunner, and Konietschke 2015; Smaga 2017). Thus, Brunner, Munzel, and Puri (1999) and Domhof, Brunner, and Osgood (2002) proposed an alternative quadratic form by deleting the variance ${\hat{V}}_{n}$ involved in the computation of the WTS, resulting in the ATS defined as

(3.2) $$\begin{array}{c} T_{A}=\frac{1}{tr(T{\hat{V}}_{n})}n{\hat{p}}^{T}T\hat{p}, \end{array}$$

where $T=C^{T}{\left[CC^{T}\right]}^{+}C$ is a projection matrix. Note that $H_{0}:TF=0\Leftrightarrow CF=0$ because $C^{T}{\left[CC^{T}\right]}^{+}$ is a generalized inverse of C.

It is also worthy to note that different to $T_{W}$, the asymptotic distribution of $T_{A}$ can also be derived if V is singular.

Theorem 3.2 Under Assumption (1) and under the null hypothesis $H_{0}:CF=0$, the test statistic $T_{A}$ has asymptotically, as $n_{0}\to \infty$, the same distribution as the random variable

(3.3) $$\begin{array}{c} A=\sum_{i=1}^{a}\sum_{j=1}^{d}\zeta_{ij}B_{ij}/tr\left(TV_{n}\right), \end{array}$$

where $B_{ij}\overset{\text{i.i.d}}{∼}\chi_{1}^{2}$ and the weights $\zeta_{ij}$ are the eigenvalues of $TV_{n}$.

The limiting distribution of A is approximated by a scaled $g\chi_{f}^{2}$ distribution, where g is a constant such that the first two moments coincide. Brunner, Munzel, and Puri (1999) proposed a Box (1954)–type approximation such that the first two moments of $T_{A}$ and $g\chi_{f}^{2}$ approximately coincide where f can be estimated by $\hat{f}=\frac{{\left(tr\left(T{\hat{V}}_{n}\right)\right)}^{2}}{tr(T{\hat{V}}_{n}T{\hat{V}}_{n})}$. Thus, the distribution of $T_{A}$ is approximated by a central $F(\hat{f},\infty )$‐distribution. The corresponding ANOVA‐type test $\phi_{A}=1\left\{T_{A}>F_{\alpha }\left(\hat{f},\infty \right)\right\}$, where $F_{\alpha }\left(\hat{f},\infty \right)$ denotes the (1−α)‐quantile of the $F(\hat{f},\infty )$‐distribution.

Despite its advantage of being applicable in case of singular covariance matrices, the ATS has the drawback of being an approximative test and thus, even its asymptotic exactness cannot be guaranteed.

Another possible test statistic is the modified version of the ATS (MATS) that was developed by Friedrich and Pauly (2018) for mean‐based MANOVA models. Here, we transfer it to the ranked‐based set‐up, where it is given by

(3.4) $$\begin{array}{c} T_{M}=n{\hat{p}}^{T}C^{T}{\left[C{\hat{D}}_{n}C^{T}\right]}^{+}C\hat{p}, \end{array}$$

where ${\hat{D}}_{n}=diag\left(\frac{n}{n_{i}}{\hat{v}}_{i}\left(j,j\right)\right),i=1,\text{…},a,j=1,\text{…},d$. In this way, it is a compromise between the WTS and the ATS as it only uses the diagonal entries of ${\hat{V}}_{n}$ for the multivariate studentization. In fact, the nonsingularity assumption of V is not needed to derive its asymptotics. It is replaced by the weaker requirement that $D=diag(\frac{n}{n_{i}}v_{i}\left(j,j\right))>0,i=1,\text{…},a,j=1,\text{…},d$, which is fulfilled when all the diagonal elements $v_{i}\left(j,j\right)$ of $V_{i}$ are positive. This is the same as $Var(H\left(X_{ij1}\right))>0$ for all i∈{1,…,a},j∈{1,…,d}. It is supposed to be met in a wide range of applicable settings, except only cases where, for example, at least one time point of any of the treatment groups is a discrete variable with very few distinct values.

Theorem 3.3 Under Assumption (1) and assuming that $v_{i}\left(j,j\right)>0$ for all i∈{1,…,a},j∈{1,…,d}, the test statistic $T_{M}$, has under the null hypothesis $H_{0}:CF=0$, asymptotically, as $n_{0}\to \infty$, the same distribution as the random variable

$$M=\sum_{i=1}^{a}\sum_{j=1}^{d}{\tilde{\zeta }}_{ij}{\tilde{B}}_{ij},$$

where ${\tilde{B}}_{ij}\overset{\text{i.i.d}}{∼}\chi_{1}^{2}$ and the weights ${\tilde{\zeta }}_{ij}$ are the eigenvalues of $V_{n}^{1/2}C^{T}{\left[CDC^{T}\right]}^{+}CV_{n}^{1/2}$ and $D=diag(\frac{n}{n_{i}}v_{i}\left(j,j\right))$.

Since, the limit distributions of both, the ATS and the MATS, depend on unknown weights $\zeta_{i}$ and ${\tilde{\zeta }}_{i}$, we cannot directly calculate critical values. In addition, the $\chi_{f}^{2}$‐approximation to $T_{W}$ is rather slow. To this end, we develop asymptotically correct testing procedures based on bootstrap versions of $T_{W},T_{A}$, and $T_{M}$ in the subsequent section.

## 4 Wild Bootstrap Approach

We consider a wild bootstrap approach to derive new asymptotically valid testing procedures with good finite sample properties. To this end, let $Z_{ik}=\left(R_{ik}-{\bar{R}}_{i.}\right)$ denote the centered rank vectors, where $R_{ik}={\left(R_{i1k},\text{…},R_{idk}\right)}^{T}$, ${\bar{R}}_{i.}={\left({\bar{R}}_{i1.},\text{…},{\bar{R}}_{id.}\right)}^{T}$, and ${\bar{R}}_{ij.}=\frac{1}{\lambda_{ij.}}\sum_{k=1}^{n_{i}}\lambda_{ijk}\left(R_{ijk}\right)$. Moreover, let $W_{ik}$ denote independent and identically distributed random weights with $E(W_{ik})=0$ and $Var(W_{ik})=1$. Although, there are different possible choices for these random weights (Mammen 1993; Davidson and Flachaire 2008), some particular choices have become popular. Following the investigations in Friedrich, Konietschke, and Pauly (2017) for the corresponding complete case scenario, and our simulation results for other common wild bootstrap weights in Section 5.4.1 below, we use Rademacher random variables, which are defined by $P\left(W_{ik}=-1\right)=P\left(W_{ik}=1\right)=1/2$. Then, a wild bootstrap sample is defined as

(4.1) $$\begin{array}{c} Z_{ik}^{*}=W_{ik}.Z_{ik},\;i=1,\text{…},a,\;k=1,\text{…},n_{i}. \end{array}$$

In other words, $Z_{ik}^{*}$ is a symmetrization of the rank vector $Z_{ik}$. Now, we can define the bootstrap version of the relative effect estimator ${\hat{p}}_{ij}$

(4.2) $$\begin{array}{c} {\hat{p}}_{ij}^{*}=\frac{1}{\lambda_{ij.}}\sum_{k=1}^{n_{i}}\lambda_{ijk}\frac{1}{N}\left(Z_{ijk}^{*}\right)=\frac{1}{\lambda_{ij.}}\sum_{k=1}^{n_{i}}\lambda_{ijk}\frac{1}{N}\left(W_{ik}\left(R_{ijk}-{\bar{R}}_{ij.}\right)\right). \end{array}$$

We pool them into the vector ${\hat{p}}^{*}={\left({\hat{p}}_{11}^{*},\text{…},{\hat{p}}_{ad}^{*}\right)}^{T}$ to obtain the bootstrap counterpart of $\hat{p}$.

In the same way, the bootstrap covariance matrix estimator ${\hat{V}}_{i}^{*}=\left[{\hat{v}}_{i}^{*}\left(j,j^{\prime }\right)\right]$ with diagonal and off‐diagonal elements ${\hat{v}}_{i}^{*}\left(j,j\right)$ and ${\hat{v}}_{i}^{*}\left(j,j^{\prime }\right)$, respectively, is given by

(4.3) $$\begin{array}{c} {\hat{v}}_{i}^{*}\left(j,j\right)=\frac{n_{i}\sum_{k=1}^{n_{i}}\lambda_{ijk}{\left[Z_{ijk}^{*}-{\bar{Z}}_{ij.}^{*}\right]}^{2}}{\left(N^{2}\right)\lambda_{ij.}\left(\lambda_{ij.}-1\right)}, \end{array}$$

(4.4) $$\begin{array}{c} {\hat{v}}_{i}^{*}\left(j,j^{\prime }\right)=\frac{n_{i}\sum_{k=1}^{n_{i}}\lambda_{ijk}\lambda_{ij^{\prime }k}\left[\left(Z_{ijk}^{*}-{\bar{Z}}_{ij.}^{*}\right)\left(Z_{ij^{\prime }k}^{*}-{\bar{Z}}_{ij^{\prime }.}^{*}\right)\right]}{\left(N^{2}\right)\left(\left(\lambda_{ij.}-1\right)\left(\lambda_{ij^{\prime }.}-1\right)+\Delta_{i,jj^{\prime }}-1\right)}. \end{array}$$

From this, the bootstrapped versions of the quadratic forms, that is, the WTS $T_{W}^{*}$, the ATS $T_{A}^{*}$, and the MATS $T_{M}^{*}$ are computed as

(4.5) $$\begin{array}{c} T_{W}^{*}=n{\hat{p}}^{*T}C^{T}{\left[C{\hat{V}}_{n}^{*}C^{T}\right]}^{+}C{\hat{p}}^{*}, \end{array}$$

(4.6) $$\begin{array}{c} T_{A}^{*}=\frac{1}{tr(T{\hat{V}}_{n}^{*})}n{\hat{p}}^{*T}T{\hat{p}}^{*}, \end{array}$$

(4.7) $$\begin{array}{c} T_{M}^{*}=n{\hat{p}}^{*T}C^{T}{\left[C{\hat{D}}_{n}^{*}C^{T}\right]}^{+}C{\hat{p}}^{*}, \end{array}$$

where ${\hat{V}}_{n}^{*}=\overset{a}{\underset{i=1}{⨁}}\frac{n}{n_{i}}{\hat{V}}_{i}^{*}$ and ${\hat{D}}_{n}^{*}=diag\left(\frac{n}{n_{i}}{\hat{v}}_{i}^{*}\left(j,j\right)\right),i=1,\text{…},a,j=1,\text{…},d$. To get an asymptotically valid bootstrap test, we have to assure that the conditional distribution of the Wald, ANOVA‐type, and MATS‐type bootstrap statistics $T_{W}^{*}$, $T_{A}^{*}$, and $T_{M}^{*}$ approximate the null distribution of $T_{W}$, $T_{A}$, and $T_{M}$, respectively.

Theorem 4.1 Under Assumption (1), the following results hold:

(1) For i=1,…,a, the conditional distribution of $\sqrt{n}{\hat{p}}_{i}^{*}$, given the data X, converges weakly to the multivariate $N(0,\kappa_{i}^{-1}V_{i})$‐distribution in probability.
(2) The conditional distribution of $\sqrt{n}{\hat{p}}^{*}$, given the data X, converges weakly to the multivariate $N(0,\overset{a}{\underset{i=1}{⨁}}\kappa_{i}^{-1}V_{i})$‐distribution in probability.

Thus, the distributions of $\sqrt{n}C{\hat{p}}^{*}$ and $\sqrt{n}C\left(\hat{p}-p\right)$ asymptotically coincide under the null hypothesis $H_{0}$.

Theorem 4.2 Under Assumption (1), for any choice [−]∈{A,M,W}, the conditional distribution of $T_{\left[-\right]}^{*}$ converges weakly to the null distribution of $T_{\left[-\right]}$ in probability for any choice of $p\in R^{ad}$. In particular, we have that

$$\begin{array}{c} \underset{x\in R}{\sup}\left|P_{p}\left(T_{\left[-\right]}^{*}\leq x|X\right)-P_{H_{0}}\left(T_{\left[-\right]}\leq x\right)\right|\overset{p}{\to }0 \end{array}$$

holds, provided that V>0 or $v_{i}\left(j,j\right)>0$ for all i∈{1,…,a},j∈{1,…,d} is fulfilled in case of $T_{W}$ or $T_{M}$, respectively.

Therefore, the corresponding wild bootstrap tests are given by $\phi_{W}^{*}=1\left\{T_{W}>c_{w}^{*}\right\}$, $\phi_{A}^{*}=1\left\{T_{A}>c_{A}^{*}\right\}$, and $\phi_{M}^{*}=1\left\{T_{M}>c_{M}^{*}\right\}$, where $c_{w}^{*}$, $c_{A}^{*}$, and $c_{M}^{*}$ denote the (1−α) quantiles of the conditional bootstrap distributions of $T_{W}^{*}$, $T_{A}^{*}$, and $T_{M}^{*}$ given the data, respectively.

Theorem 4.2 implies that the wild bootstrap tests are of asymptotic level α under the null hypothesis and consistent for any fixed alternative $H_{1}:CF\neq 0$, that is, they have asymptotically power 1. In addition, it follows from Jansen et al. (2003) that they have the same local power under contiguous alternatives as their original tests.

## 5 Monte Carlo Simulations

The above results are valid for large sample sizes. To analyze the finite sample behavior of the asymptotic quadratic tests and their wild bootstrap counterparts described in Sections 3 and 4, we conduct extensive simulations. As an assessment criteria, all procedures were studied with respect to their

(i) type‐I error rate control at level α=5% and their
(ii) power to detect deviations from the null hypothesis.

All simulations were operated by means of the R computing environment, version 3.2.3 (R Core Team 2013) and each setting was based on 10,000 simulation runs and B=999 bootstrap runs. The algorithm for the computation of the p‐value of the wild bootstrap tests in any of the test statistics $T\in \{T_{A},T_{W},T_{M}\}$ is as follows:

(1) For the given incomplete multifactor data, calculate the observed test statistic, say T.
(2) Compute the rank values $Z_{ik}$.
(3) Using i.i.d Rademacher weights $W_{i1},\text{…},W_{in_{i}}$, generate bootstrapped rank values $Z_{ik}^{*}=W_{ik}.Z_{ik}$.
(4) Calculate the value of the test statistic for the bootstrapped sample $T^{*}$.
(5) Repeat the Steps 3 and 4 independently B=999 times and collect the observed test statistic values in $T_{b}^{*},b=1,\text{…}..,B$.
(6) Finally, estimate the wild bootstrap p‐value as p‐value $=\frac{\sum_{b=1}^{B}I\left(T_{b}^{*}>=T\right)}{B}$.

We considered a two‐way layout design with a=2 independent groups and two different time points d∈{4,8} underlying discrete and continuous distributions. As in Konietschke et al. (2015) and Bathke et al. (2018) for the case of complete observations, we investigated balanced situations with sample size vectors $\left(n_{1},n_{2}\right)\in \left\{\left(5,5\right),\left(10,10\right),\left(20,20\right)\right\}$, and an unbalanced situation with sample size vector $\left(n_{1},n_{2}\right)=\left(10,20\right)$. Since the empirical type‐I error rates of the tests in sample sizes (10,20) and (20,10) are very similar, we omit (20,10). In particular, we studied three different kinds of hypotheses: the hypothesis of no group effect $H_{0}^{G}:\left(P_{a}⊗\frac{1}{d}1_{d}^{T}\right)$, no time effect $H_{0}^{T}:\left(\frac{1}{a}1_{a}^{T}⊗P_{d}\right)$, as well as no interaction effect $H_{0}^{GT}:\left(P_{a}⊗P_{d}\right)$.

For each scenario, we generated missingness within MCAR as well as MAR frameworks as described below: For the MCAR mechanism, we simulated $X_{ik}=\left(\delta_{i1k}X_{i1k},\text{…},\delta_{idk}X_{idk}\right),\;k=1,\text{…},n_{i}$, for independent Bernoulli distributed $\delta_{i1k}∼B\left(r\right)$ and a zero entry was interpreted as a missing observation. The missing probability was chosen from r∈{10%,30%}.

For the *MAR mechanism*, we considered several pairs of features $\left\{X_{obs},X_{miss}\right\}$. For each pair, there is a determining feature $X_{obs}$ that determines the missing pattern of its corresponding $X_{miss}$ (Santos et al. 2019). Thus, for d=4, we define the pairs $\left\{X_{i1k},X_{i2k}\right\}$ and $\left\{X_{i3k},X_{i4k}\right\}$. Whereas, for d=8, we define the following pairs $\left\{X_{i1k},X_{i2k}\right\},\left\{X_{i1k},X_{i3k}\right\},\left\{X_{i6k},X_{i7k}\right\}$, and $\left\{X_{i6k},X_{i8k}\right\}$. Two different MAR scenarios are investigated—MAR1 and MAR2. In MAR1, for each pair, we divided X into three groups based on their $X_{obs}$ values: The first group is given by $\left\{X_{ik}:X_{obs}\in \left(-\infty ,-2{\hat{\sigma }}_{1}\right),k=1,..,n_{i}\right\}$, the second by $\left\{X_{ik}:X_{obs}\in \left(-2{\hat{\sigma }}_{1},2{\hat{\sigma }}_{1}\right),k=1,..,n_{i}\right\}$, and the last group by $\left\{X_{ik}:X_{obs}\in \left(2{\hat{\sigma }}_{1},\infty \right),k=1,..,n_{i}\right\}$, where ${\hat{\sigma }}_{1}$ is the estimated standard deviation of $X_{obs}$. Then, we randomly inserted missing values on $X_{miss}$ based on the following missing percentages: 15% for group 1 and three and 30% for the second group.

For generating MAR2 scenario, we considered the median of each $X_{obs}$ to define the missing pattern of $X_{miss}$ (Zhu, He, and Liatsis 2012; Pan et al. 2015). For each pair from above, two groups were defined: The first one is $\left\{X_{ik}:X_{obs}\in \left(-\infty ,median\left(X_{obs}\right)\right],k=1,..,n_{i}\right\}$, and the second is $\left\{X_{ik}:X_{obs}\in \left(median\left(X_{obs}\right),\infty \right),k=1,..,n_{i}\right\}$. Then, missing values were inserted on $X_{miss}$ as follows: 10% for group one and 30% for the second group.

### 5.1 Continuous Data

To investigate the type‐I error control of the suggested methods, data samples were generated from the model

$$X_{ik}∼F_{i}\left(\mu_{0},\Sigma_{i}\right),\;i=1,2,\;k=1,\text{…},n_{i},$$

where $F_{i}\left(\mu_{0},\Sigma_{i}\right)$ represents a multivariate distribution with expectation vector $\mu_{0}$ and covariance matrix $\Sigma_{i}$. Marginal data distributions were generated from different symmetric distributions (normal, double exponential) as well as skewed distributions (lognormal, $\chi_{15}^{2}$) (similar to Pauly, Brunner, and Konietschke 2015). We used normal copulas to generate the dependency structures of the repeated measurements using the R package **copula** (Yan et al. 2007). For the covariance matrix $\Sigma_{i}$, we investigated the three following covariance structures

(AR) Setting 1: $\Sigma_{i}={\left(\rho^{\left|l-j\right|}\right)}_{l,j\leq d},\rho =0.6$ for i=1,2,
(CS) Setting 2: $\Sigma_{i}=I_{t}$, for i=1,2,
(TP) Setting 3: $\Sigma_{i}={\left(d-|l-j|\right)}_{l,j\leq d}$, for i=1,2,

representing an autoregressive structure (Setting 1), compound symmetry pattern (Setting 2), and a linear Toeplitz covariance structure (Setting 3). These covariance settings were inspired by the simulations studies in Konietschke et al. (2015), Friedrich, Konietschke, and Pauly (2017), and Umlauft et al. (2019).

**FIGURE 1:**
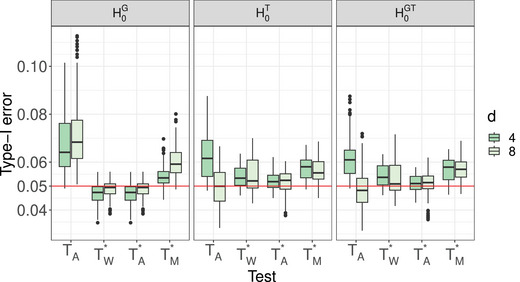
Type‐I error of the asymptotic ANOVA‐type test $T_{A}$ and the bootstrapped tests $T_{W}^{*}$, $T_{A}^{*}$, and $T_{M}^{*}$ based on several hypotheses of interest for varying time points d∈{4,8}. Each boxplot summarizes the type‐I error results from 96 different simulation scenarios for this hypothesis. For all individual simulations, see the tables in the Supporting Information.

**FIGURE 2:**
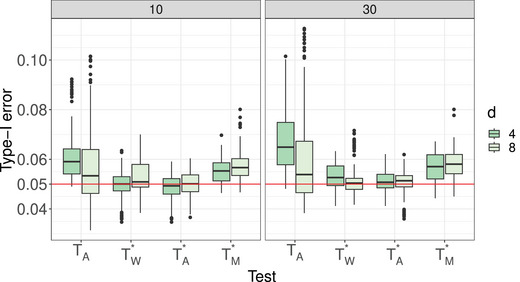
Type‐I error of the asymptotic ANOVA‐type test $T_{A}$ and the bootstrapped tests $T_{W}^{*}$, $T_{A}^{*}$, and $T_{M}^{*}$ based on missing rates r∈{10,30} for varying time points d∈{4,8}. Each boxplot summarizes the type‐I error results from 144 different simulation scenarios for this missing rate. For all individual simulations, see the tables in the Supporting Information.

**Type‐I Error Results**. The type‐I error simulation results of the studied procedures for testing the hypotheses of no time effect $H_{0}^{T}$, no group × time interaction $H_{0}^{GT}$, and no group effect $H_{0}^{G}$ under the MCAR and MAR frameworks are shown in Tables S.1 and S.12 (MCAR framework) and Tables S.13–S.24 (MAR framework) in the Supporting Information. It can be readily seen that the suggested bootstrap approaches based on $T_{W}^{*},T_{A}^{*}$, and $T_{M}^{*}$ tend to result in quite accurate type‐I error rate control for most hypotheses under symmetric as well as skewed distributions and under MCAR and MAR mechanisms. The type‐I error control is surprisingly not affected by less stringent missing mechanisms and the bootstrap tests are robust under fairly large amounts of missing observations. In addition, the data dependency structures do not affect the quality of the approximations. The sample size allocations (balanced vs. unbalanced) slightly impact the type‐I error of the tests. On the other hand, the asymptotic ANOVA‐type test $T_{A}$ also shows a quite accurate type‐I error control for large sample sizes. However, under small sample sizes, $T_{A}$ tends to be sensitive to the missing rates. In particular, it exhibits a liberal behavior for larger missing rates. In contrast, the asymptotic Wald test $T_{W}$ shows an extremely liberal behavior in all considered situations and under all investigated missing mechanisms. A closer look at the type‐I error simulation results for all considered settings under the MCAR framework is provided. Compact boxplots factorized in terms of hypotheses of interest or missing rates are given in Figures 1 and 2, respectively. They are based on different sample size settings (4) × different distributions (4) × different missing rates (2) × different covariance structures (3) × different time points (2) × different hypotheses (3). Due to the extreme liberal behavior of the asymptotic Wald test, it has been removed from those plots in order to get a better view of the remaining tests behavior. It can be clearly seen that the asymptotic ANOVA‐type test has the less accurate control among all other considered tests. The hypothesis of interest and number of time points obviously impact the quality of the approximations of the asymptotic ANOVA‐type test. Furthermore, the type‐I error control behavior of the bootstrapped MATS depends on the hypothesis of interest. Consequently, the bootstrapped Wald and ANOVA‐type tests are recommended over all considered methods.

**FIGURE 3:**
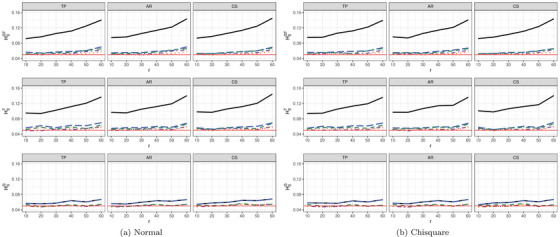
Type‐I error simulation results (α=0.05) of the tests $T_{W}$ (−−−−−−), $T_{A}$ 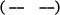, $T_{W}^{*}$ 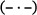, $T_{A}^{*}$ 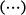, and $T_{M}^{*}$ 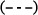 under different covariance structures with sample sizes $\left(n_{1},n_{2}\right)=\left(15,15\right)$ and d=4 for varying percentages of MCAR data r∈{10%,20%,30%,40%,50%,60%} with observations generated from (a) a normal and (b) a $\chi_{15}^{2}$ distribution, respectively.

In order to cover the *effect of increasing missing rates*, we additionally studied type‐I error control for a=2 groups, d=4 time points, $\left(n_{1}=15,n_{2}=15\right)$ sample sizes with r∈{10%,20%,30%,40%,50%,60%} covering missingness in observations ranging from 10% to 60%. Figure 3 and Figures S.1 and S.2 in the Supporting Information summarize type‐I error rate control for these settings under symmetric and asymmetric distributions. The results indicate that the asymptotic Wald test $T_{W}$ tends to be liberal in all considered situations. In particular, it is extremely liberal when testing the hypotheses of no time effect ($H_{0}^{T}$) and no interaction effect ($H_{0}^{GT}$). In contrast, the asymptotic ANOVA‐type test $T_{A}$ tends to be sensitive to missing rates and hypothesis type. In particular, it exhibits an accurate or liberal behavior for small or large missing rates, respectively. Moreover, it shows a quite constant liberal behavior when testing the hypothesis of no group effect ($H_{0}^{G}$). Its behavior appears to be independent of the covariance pattern. In contrast, the suggested bootstrap approaches tend to control type‐I error rate more accurate over the range of missing rates r for almost all settings.

Further, it was also interesting to discover the type‐I error rate control of the tests under *similar attributes to the data sets of the fluvoxamine and skin disorder clinical trials*. The data sets reflect large sample sizes and a small or moderate amount of missing values from either a one‐sample repeated measurements design or a two‐way layout design. Simulation results for the type‐I error rate of the studied procedures for (n=299,d=3) and ($n_{1}=88,n_{2}=84,d=3$) sample sizes are presented in Table S.25 and Table S.26 in the Supporting Information, respectively. It can be seen that most tests are robust under both settings and control type‐I error rate accurately. Only the Wald‐type tests exhibit a liberal behavior for some of the lognormal settings.

In addition, we investigated the *impact of increasing the number of groups* on type‐I error rate control. Starting with two groups, we systematically expanded the number of groups up to 12. Our observations were generated from a lognormal distribution, with sample sizes set in an unbalanced manner as follows: $n=\left(n_{1},\text{…},n_{12}\right)=\left(10,10,15,20,35,25,15,30,20,35,20,15\right)$. We considered two time points (d∈{4,8}), an autoregressive covariance structure (Setting 1), chosen for its similarity to other structures, and a missing rate of 30%. This simulation setup was inspired by a previous study on repeated measures designs with a potentially large number of groups by Sattler (2021). Figure 5 and Figure S.3 in the Supporting Information, corresponding to hypotheses $H_{0}^{G}$ and $H_{0}^{GT}$, respectively, illustrate that the asymptotic Wald test $T_{W}$ consistently demonstrates a liberal behavior across all scenarios. However, the asymptotic ANOVA‐type test $T_{A}$ shows sensitivity to the number of time points d, displaying more accurate behavior for a small number of time points (d=4). In contrast, the suggested bootstrap ANOVA‐type test $T_{A}^{*}$ tends to provide more accurate control of the type‐I error rate across almost all settings and among all considered tests. Moreover, it is noteworthy that the simulation results reveal a consistent pattern: When considering scenarios under the hypothesis $H_{0}^{G}$, the behavior of the WTS $T_{W}$ and its respective wild bootstrap version $T_{W}^{*}$ deteriorates with an increasing number of groups, while the opposite is observed for the two ANOVA‐type approaches.

**FIGURE 4:**
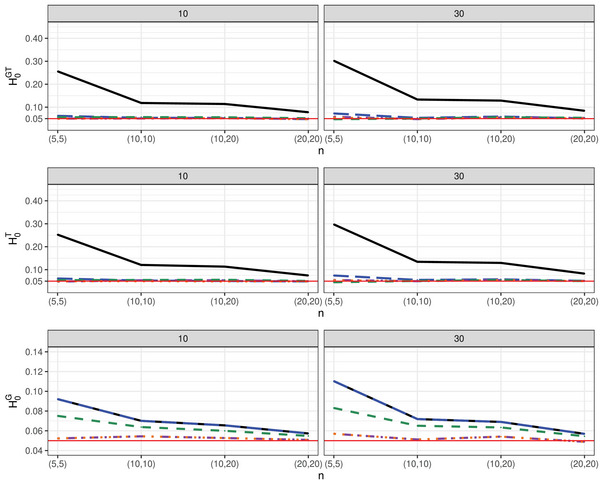
Type‐I error simulation results (α=0.05) of the tests $T_{W}$ (−−−−−−), $T_{A}$ 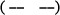, $T_{W}^{*}$ 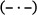, $T_{A}^{*}$ 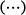, and $T_{M}^{*}$ 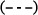 for ordinal data under MCAR framework with sample sizes $\left(n_{1},n_{2}\right)=\left\{\left(5,5\right),\left(10,10\right),\left(10,20\right),\left(20,20\right)\right\}$, and d=4.

**FIGURE 5:**
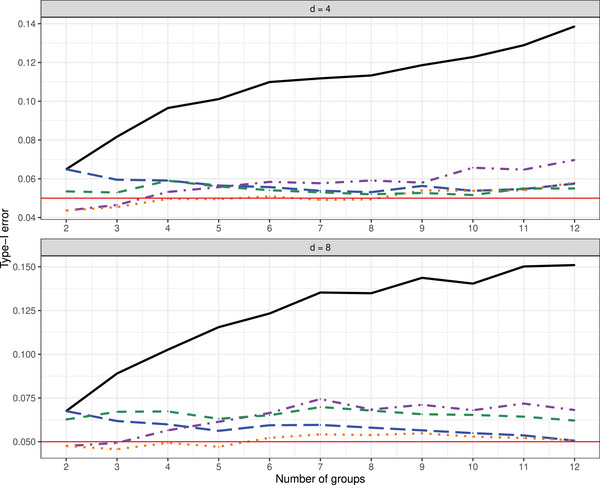
Type‐I error simulation results (α=0.05) of the tests $T_{W}$ (−−−−−−), $T_{A}$ 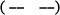, $T_{W}^{*}$ 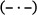, $T_{A}^{*}$ 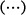, and $T_{M}^{*}$ 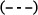 for increasing number of groups under the MCAR framework with sample sizes $n=\left(n_{1},\cdots ,n_{12}\right)=\left(10,10,15,20,35,25,15,30,20,35,20,15\right)$, d∈{4,8} with observations generated from a lognormal distribution and under the hypothesis $H_{0}^{G}$.

### 5.2 Ordinal Data

In order to address all the goals outlined above, we simulated ordinal data. The observations were simulated similar to Brunner and Langer (2000) as follows:

$$\begin{array}{ccc} X_{ijk} & = & int\left(4\frac{cZ_{ik}+Y_{ijk}}{c+1}\right)+1,\;i=1,\text{…},a,\;k=1,\text{…},n_{i},\\ & & j=1,\text{…},d, \end{array}$$

where $Z_{ik}$ and $Y_{ijk}$ are independently uniformly distributed in the interval [0,1], c>0 is a constant, and int(x) indicates the integer part of x. The elements of $X_{ijk}$ take values between 1 and 4. The correlation between $X_{ij^{`}k}$ and $X_{ijk}$ is determined by the choice of the constant c. In our simulation study, we considered c=1 assuring a compound symmetric covariance structure. We considered a=2 groups and d∈{4,8} dimensions, and the same sample sizes as in the continuous data settings.

The type‐I error results of the considered methods under MCAR and MAR frameworks are summarized in Figure 4 and Figures S.4–S.6 in the Supporting Information. It can be seen that the asymptotic Wald test is too liberal in most situations and under all considered hypotheses. The ANOVA‐type test of Brunner, Munzel, and Puri (1999) shows much better behavior. In contrast, the type‐I error control for the bootstrap‐based procedures is the best, particularly for the bootstrapped Wald and ANOVA‐type tests, which are also less affected by an increased missing rate or strict MAR assumptions.

### 5.3 Power

In order to assess the empirical power of all studied methods, we considered a one‐sample repeated measures design with d=4 repeated measures, sample size n=15, and covariance structures as given in Settings 1−3 for various distributions. Data were generated by

$$\begin{array}{c} \left[X_{1k}∼F_{1}\left(\mu_{0},\Sigma_{1}\right)\right]+\mu_{1},\;k=1,\text{…},15, \end{array}$$

where, we were interested in detecting two specific alternatives

- Alternative 1: $\mu_{1}=\left(0,0,\zeta ,\zeta \right)$,
- Alternative 2: $\mu_{1}=\left(0,0,0,\zeta \right)$,

for varying shift parameter ζ∈{0,0.5,1,1.5,2,2.5,3}.

**FIGURE 6:**
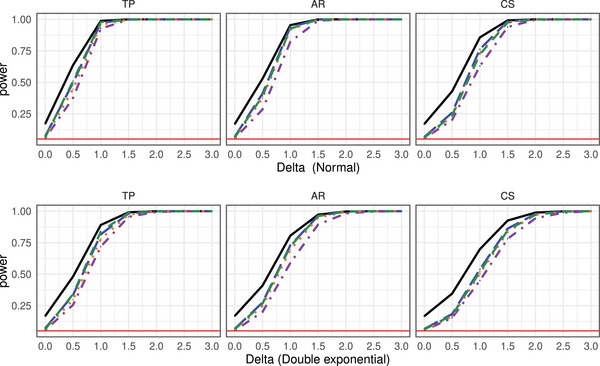
Power simulation results of the tests $T_{W}$ (−−−−−−), $T_{A}$ 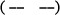, $T_{W}^{*}$ 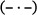, $T_{A}^{*}$ 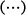, and $T_{M}^{*}$ 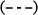 under different covariance structures with sample size n=15 and d=4 under alternative 1, under the MCAR framework with missing rate r=30% with observations generated from a normal (upper row) and a double exponential (bottom row) distribution, respectively.

**FIGURE 7:**
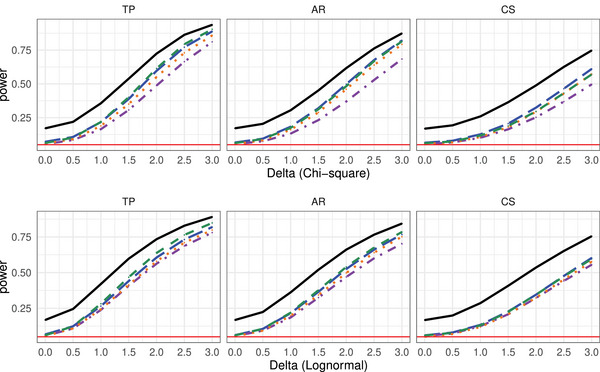
Power simulation results of the tests $T_{W}$ (−−−−−−), $T_{A}$ 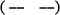, $T_{W}^{*}$ 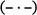, $T_{A}^{*}$ 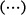, and $T_{M}^{*}$ 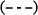 under different covariance structures with sample size n=15 and d=4 under alternative 1, under the MCAR framework with missing rate r=30% with observations generated from a $\chi_{15}^{2}$ (upper row) and a lognormal (bottom row) distribution, respectively.

The power analysis results of the considered methods under the MCAR framework for several distributions, involving various covariance settings for detecting Alternative 1 are summarized in Figures 6 and 7 (missing rate r=30%) and Figures S.7 and S.8 in the Supporting Information (r=10%). The simulation results for investigating Alternative 2 are displayed in Figures S.9 and S.12 in the Supporting Information. The power analysis results of the considered methods under the respective MAR framework are summarized in Figures S.13–S.16 (MAR1 scenario) and Figures S.17–S.20 (MAR2 scenario).

As the asymptotic Wald test is too liberal compared to the other studied methods, its power function is larger. Moreover, the bootstrapped Wald test exhibits the lowest power behavior, while the bootstrapped MATS and ANOVA‐type have a quite similar power behavior as the Brunner, Munzel, and Puri (1999) ANOVA‐type test. The differences between the procedures is less pronounced under the MAR framework.

To sum up, we recommend the bootstrap ATS. It exhibits the overall best type‐I error control combined with a good power behavior and needs the less stringent assumptions for application.

### 5.4 Additional Comparative Simulation Results

In this section, we present additional comparative simulation results for other wild bootstrap weights (5.4.1) and a comparison with recent procedures that infer hypotheses in terms of nonparametric effects (5.4.2).

#### 5.4.1 Considering Different Weights in the Wild Bootstrap Method

Here, we investigate the impact of different wild bootstrap weights $W_{ik}$ on the performance of our methods, aiming to identify any potentially “optimal” weights that may enhance our tests' effectiveness. We examine the following weight distributions as previously proposed in the literature in other contexts:

- Rademacher weights as above: $P\left(W_{ik}=-1\right)=P\left(W_{ik}=1\right)=1/2$, see also Liu (1988) and Friedrich, Konietschke, and Pauly (2017).
- Mammen weights: $P\left(W_{ik}=-\left(\sqrt{5}-1\right)/2\right)=\left(\sqrt{5}+1\right)/\left(2\sqrt{5}\right),P\left(W_{ik}=\left(\sqrt{5}+1\right)/2\right)=1-\left(\sqrt{5}+1\right)/\left(2\sqrt{5}\right)$, see Mammen (1993).
- Normal weights: $W_{ik}∼N\left(0,1\right)$, see Lin (1997).
- Centered Poisson weights: $W_{ik}∼Po\left(1\right)-1$, see Beyersmann, Termini, and Pauly (2013).

**FIGURE 8:**
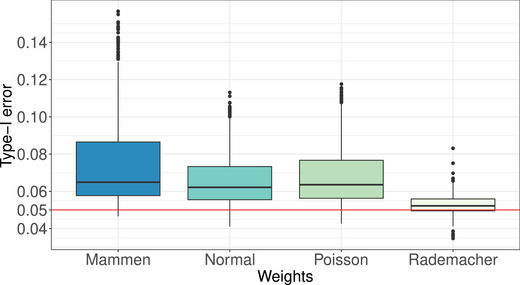
Type‐I error for three different wild bootstrap versions of our bootstrapped tests $T_{W}^{*}$, $T_{A}^{*}$, and $T_{M}^{*}$. Each boxplot summarizes the type‐I error results from 288 different simulation scenarios.

Normal and centered Poisson weights have been recommended in the survival setting (the above references) while Mammen weights were proposed in the context of high‐dimensional linear models (Mammen 1993). We compare the performance of these weight distributions with our suggested wild bootstrap tests and summarize the results in Figure 8. Notably, we find that Rademacher weights exhibit the best type‐I error control among the four different approaches. Consequently, we recommend the adoption of Rademacher weights due to their superior performance compared to the other three weight distributions examined.

#### 5.4.2 Comparison With Further Alternative Approaches

This section provides a comparative analysis between our proposed methods and recent approaches based on asymptotics rather than resampling techniques (Rubarth, Pauly, and Konietschke 2022; Rubarth et al. 2022). These alternative approaches target specific scenarios: The former paper addresses repeated measures designs, which can be regarded as a special case of our design, while the latter deals with clustered data. Both our methods and the methods proposed by Rubarth, Pauly, and Konietschke (2022) and Rubarth et al. (2022) use all‐available data and are valid under the MCAR mechanism. However, they differ in their scope: While Rubarth, Pauly, and Konietschke (2022) and Rubarth et al. (2022) concentrate on hypotheses formulated in terms of relative marginal effects, our proposed procedures are constrained to testing null hypotheses formulated in terms of the equality of the distribution functions. Consequently, the two approaches are not directly comparable. However, one can show that our null hypothesis implies the null hypothesis formulated in terms of relative marginal effects (Brunner, Bathke, and Konietschke 2018). This enables a rough comparison between the behaviors of these approaches. Given the distinct setups studied in the aforementioned papers, we conducted two separate simulation studies aligning with the statistical model designs outlined in each paper.

**FIGURE 9:**
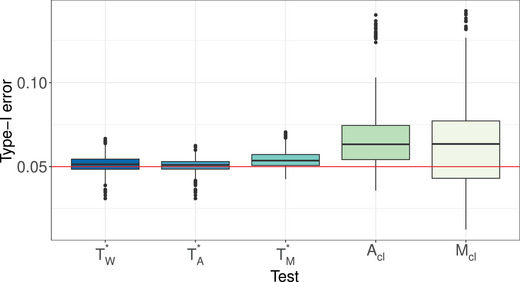
Type‐I error for three different wild bootstrap versions of our bootstrapped tests $T_{W}^{*}$, $T_{A}^{*}$, and $T_{M}^{*}$ and the two asymptotic tests of Rubarth et al. (2022) $A_{cl}$ and $M_{cl}$. Each boxplot summarizes the type‐I error results from 432 different simulation scenarios.

(1) **Alternative methods developed for repeated measures designs:**
  We investigate the approaches proposed by Rubarth, Pauly, and Konietschke (2022), which introduce nonparametric methods for analyzing repeated measures designs with missing data. They developed various test procedures, including global and multiple contrast tests, to test the null hypothesis $H_{0}^{p^{*}}:Cp^{*}=0$, where the unweighted relative marginal effect are collected in the vector $p^{*}={\left(p_{1}^{*},\text{…},p_{d}^{*}\right)}^{T}$. Here, $p_{i}^{*}=\int G\left(x\right)dF_{i}\left(x\right)$ denotes the unweighted relative effects, while $G\left(x\right)=d^{-1}\sum_{i=1}^{d}F_{i}\left(x\right)$ is the unweighted average of all distribution functions in the experiment. They developed generalized versions of the quadratic form tests proposed by Domhof, Brunner, and Osgood (2002) to test the less stringent hypothesis $H_{0}^{p^{*}}:\left\{Cp^{*}=0\right\}$. We note that $H_{0}^{p^{*}}$ implies our null hypothesis $H_{0}:CF=0$.
  Based on their simulation study and recommendations, we compare our approaches with their generalized Domhof, Brunner, and Osgood (2002) statistic (A1), their newly introduced ATS (A2) with its distribution approximated via the Greenhouse–Gaisser (Box 1954) method, as well as their maximum‐type statistic (M). Since their paper focuses on testing the hypotheses of no time effect, we solely investigated the hypothesis $H_{0}^{T}$. Motivated from above, we considered a one‐sample repeated measures design with two different time points d∈{4,8}, sample sizes n∈{10,15,20,30}, and covariance structures as specified in Settings 1–3 for various distributions. The simulation results are provided in Tables S.27–S.30 in the Supporting Information. A glimpse of the analysis results for the specific choice of normally distributed data is presented in Table 1. The results reveal that our resampling methods are more favorable with respect to type‐I error control. In fact, the maximum‐type testing procedure (M) tends to be liberal across nearly all considered scenarios, while in most scenarios with smaller sample sizes, the other two approaches (A1 and A2) also tend to be liberal. In contrast, our newly proposed methods perform well. A likely reason is that their methods were developed for testing the more complex null hypothesis formulated in terms of effect sizes. They need to estimate way more parameters and therefore they are more liberal in small samples.
(2) **Alternative methods developed for factorial clustered data designs:**
  Here, we investigate the methods proposed by Rubarth et al. (2022), which addresses factorial repeated measures designs with clustered data, extending aforementioned research by Rubarth, Pauly, and Konietschke (2022). Similar to Rubarth, Pauly, and Konietschke (2022), they propose quadratic‐ and maximum‐type testing procedures for testing the null hypothesis $H_{0}^{p}$. Rubarth et al. (2022) introduce a WTS, an ATS, and a maximum‐type statistic. Given the liberal behavior of the WTS in small or moderate sample size scenarios, they recommend the other two, denoted as $A_{cl}$ and $M_{cl}$, respectively, in our simulation results. As their model can handle clustered data structure, our model is contained within theirs as a special case. To allow comparison between their methods and ours, we set the number of possibly dependent replicates of any subject in any group at any time to be $m_{ijk}=1$. We examined similar simulation scenarios to those in our study. Specifically, we adopted a two‐way layout design with a=2 independent groups and d=3. We explored both balanced and unbalanced situations with sample size vectors $\left(n_{1},n_{2}\right)\in \left\{\left(5,5\right),\left(10,10\right),\left(20,20\right),\left(10,20\right),\left(15,15\right),\left(30,30\right)\right\}$, alongside the three hypotheses: $H_{0}^{G}$, $H_{0}^{T}$, and $H_{0}^{GT}$ under the MCAR framework. Our findings, presented in Figure 9 and Tables S.31–S.38 in the Supporting Information, indicate that the methods $A_{cl}$ and $M_{cl}$, developed by Rubarth et al. (2022), have problems in keeping the type‐I error rate in our situation. In particular, they exhibit a liberal behavior across various scenarios. In contrast, our proposed methods, especially the bootstrapped version of the ANOVA type‐test $T_{A}^{*}$, demonstrate much more favorable behavior. Again, a likely reason is that their methods have been developed for a more complex setting.

**TABLE 1:** Simulation results for type‐I error level (α=0.05) for normal distribution and different percentages r in case of the MCAR mechanism.

			r=10						r=30					
*d*	Cov	*n*	WBtstrp			Alternatives			WBtstrp			Alternatives		
			$T_{W}^{*}$	$T_{A}^{*}$	$T_{M}^{*}$	A1	A2	M	$T_{W}^{*}$	$T_{A}^{*}$	$T_{M}^{*}$	A1	A2	M
4	AR	10	5	5.2	5.7	7.5	5	10.3	6.2	5.9	7.4	9.6	7.1	15
		15	5.4	5.3	5.8	6.8	5.3	8.7	5.8	5.7	6.3	8.1	6.4	11.1
		20	4.9	4.9	5.2	5.9	4.9	7.1	4.6	5	5.3	6.7	5.5	8.9
		30	5.6	5.6	5.7	6.2	5.6	6.7	5.5	5.6	5.8	6.7	6	7.8
	CS	10	5	5	5.9	7.3	4.7	10.6	6.3	5.8	6.9	9.3	6.3	14.7
		15	5.1	5	5.3	6	4.5	8.1	5.3	5	5.7	7.2	5.5	10.4
		20	5.4	5.5	5.6	6.3	5.2	7.2	5.4	5.8	6	7.3	6	9.5
		30	5.4	5.3	5.6	5.7	5.1	6.7	5.4	5.3	5.8	6.3	5.5	8
	TP	10	5.1	6.1	6.7	8.9	6.5	11	5.9	6	7.2	9.8	7.2	14.3
		15	5.3	5.6	5.8	7.5	6.2	8.5	5.3	5.4	5.8	8.1	6.6	10.6
		20	4.9	5.8	5.8	7.1	6.2	7.5	5.8	6	6.4	7.9	6.9	9.4
		30	4.8	5.2	5.4	6.3	5.5	6.3	5.4	5.4	5.6	6.4	5.8	7.6

## 6 Application to Empirical Data

### 6.1 The Fluvoxamine Trial

In this section, we re‐examine a clinical fluvoxamine study. It has already been analyzed by Molenberghs and Lesaffre (1994), Molenberghs, Kenward, and Lesaffre (1997), Van Steen et al. (2001), Jansen et al. (2003), Molenberghs and Verbeke (2004), and Molenberghs and Kenward (2007). The study has been performed to establish the profile of fluvoxamine in ambulatory clinical psychiatric operations. Hereby, 315 patients who suffer from depression, panic disorder, and/or obsessive‐compulsive disorder were scored every 2 weeks over 6 weeks of treatment (*d* = 3). At each clinical visit, scores for both *side effect* and *therapeutic effect* scales, which are based on about 20 psychiatric symptoms were recorded. The side effect scale ranges from 1 to 4. The lower the score, the better the clinical record. For example, score 1 stands for “no side effect” while score 4 indicates that “the side effect surpasses the therapeutic effect.” Similarly, the therapy effect is a four‐category ordinal scale: (1) “no improvement or worsening”; (2) “minimal improvement, not changing functionality”; (3) “moderate improvement, partial disappearance of symptoms”; and (4) “important improvement, almost disappearance of symptoms.” The higher the score, the better the patients health.

**FIGURE 10:**
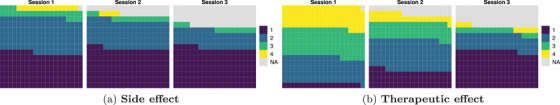
Frequencies of the side effect and therapeutic effect scores observed in the fluvoxamine trial.

Several patients missed the recording of their measurements in some sessions, which leaded to a large amount of missing values. A closer look to our data shows that, from the total of 315 initially recruited patients, 14 patients dropped off, and two were excluded from the analysis due to their nonmonotone missing pattern. This leaves us with a total of 299 patients who have at least one measurement. Among them, 242 patients have complete observations, 31 were scored only on the first session, and 44 were scored on both Session 1 and Session 3. Waffle plots representing the distributions of the side effect and therapeutic effect among the three sessions are shown in Figure 10. We aim to test the hypotheses whether side effect or therapeutic effect scores are significantly different between the three sessions for patients with psychiatric disorder. To this end, we applied all considered testing methods; asymptotic Wald and ANOVA‐type tests ($T_{W},T_{A}$), and the bootstrap procedures ($T_{W}^{*}$, $T_{A}^{*}$, $T_{M}^{*}$) to detect the null hypothesis $H_{0}^{T}:\left\{CF=0\right\}$. The results are summarized in Table 2. It can be seen that all tests indicate a significant difference between the therapeutic effect scores as well as the side effect scores of the three sessions (two‐sided *p*‐value <0.00001). Moreover, we conclude that the clinical outcome of the patients significantly improves after three sessions. These findings coincide with that in Molenberghs and Kenward (2007).

**TABLE 2:** *p*‐Values of the fluvoxamine study.

Effect	$T_{W}$	$T_{A}$	$T_{W}^{*}$	$T_{A}^{*}$	$T_{M}^{*}$
Therapeutic effect	$2.3e^{-79}$	$2.2e^{-86}$	0	0	0
Side effect	$2.3e^{-10}$	$4.1e^{-12}$	0	0	0

### 6.2 The Skin Disorder Trial

**FIGURE 11:**
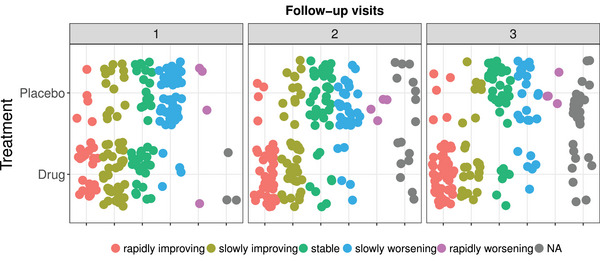
Frequencies of observed patients treatment outcome in the skin disorder trial.

Here, we study data from a randomized, multicenter, parallel group study for treating a skin condition. Treating skin conditions can be tough, thus the goal of the study was to assess the severe rate of the skin condition over time and to compare the efficiency and safety of two continuous therapy treatments drug and placebo. Patients were randomly assigned to drug or placebo therapy treatment. Prior to treatment, patients were assessed to determine the initial severity of the skin condition (moderate or severe). At three follow‐up visits, the treatment outcome was measured according to a five‐point ordinal response scale that assess the extent of improvement (1 = rapidly improving, 2 = slowly improving, 3 = stable, 4 = slowly worsening, 5 = rapidly worsening). The study consists of 88 and 84 subjects allocated to the active treatment group and the placebo group, respectively. And, the proportion of missing observations is around 30%. The distribution of patients improvement across the treatment groups and the follow‐up visits is displayed in Figure 11. The study is described in full detail in Landis et al. (1988) and is published in Davis (2002). There is not enough information available from the data source regarding the missingness mechanism. It is likely that the missingness is not at random, considering the nature of the study. Nonetheless, for the sake of illustration, we continue with our analysis.

Similar to the fluvoxamine study above, we applied all asymptotic and bootstrap procedures to infer the following null hypotheses: “no group effect,” “no time effect,” and “no group × time effect.” The results are summarized in Table 3: All approaches reject the null hypothesis of no group effect, the null hypothesis of no time effect, and the null hypothesis of no treatment group × time interaction. This implies that the clinical outcome of the patients significantly improves with time and this progression is significantly different between the two treatment groups, drug and placebo. Therefore, the data are further analyzed and split by the factor initial severity and the analysis is replicated separately for each baseline severity level (moderate or severe). The results are provided in Table 4.

**TABLE 3:** *p*‐Values of the skin disorder trial.

Hypothesis	$T_{W}$	$T_{A}$	$T_{W}^{*}$	$T_{A}^{*}$	$T_{M}^{*}$
Group	0	0	0	0	0
Time	0	0	0	0	0
Group × Time	0.032	0.016	0.026	0.01	0.009

**TABLE 4:** *p*‐Values of the split skin disorder trial based on initial severity.

	Initial severity = Moderate					Initial severity = Severe				
Hypothesis	$T_{W}$	$T_{A}$	$T_{W}^{*}$	$T_{A}^{*}$	$T_{M}^{*}$	$T_{W}$	$T_{A}$	$T_{W}^{*}$	$T_{A}^{*}$	$T_{M}^{*}$
Group	0	0	0	0	0	0	0	0	0	0
Time	0.003	0.004	0.003	0.002	0.002	0	0	0.001	0	0
Group × Time	0.041	0.032	0.043	0.027	0.029	0.423	0.318	0.447	0.338	0.335

It can be seen from Table 4 that all approaches detect a significant group effect as well as a significant time effect in both moderate and severe groups. In contrast, a significant group × time interaction effect arises only in the moderate severity group. This indicates that the change in clinical outcomes of patients of moderate severity varies over time depending on treatment group membership. All five approaches share the previous findings.

## 7 Summary

Multigroup repeated measures design with ordinal or skewed observations are quite common. If the observations are additionally subject to missing values existing methods for testing null hypotheses in terms of distribution function may be either liberal (WTSs) or run (asymptotically) on a wrong type‐I error level (ATS; Domhof, Brunner, and Osgood 2002). To this end, we investigated three alternatives based on resampling. We proved their asymptotic validity and analyzed their small sample behavior regarding type‐I error control and power in extensive simulations. Under all of the five considered methods, an ATS with critical values calculated by means of a Wild bootstrap approach exhibits the best behavior and is recommended.

In the future, we will include the present methodology into the R package nparLD (Noguchi et al. 2012). Moreover, we plan to extend our investigations to general MANOVA settings, for example, extending the results of Dobler, Friedrich, and Pauly (2020) to the situation with missing values.

## Conflicts of Interest

The authors have declared no conflicts of interest.

### Open Research Badges

This article has earned an Open Data badge for making publicly available the digitally‐shareable data necessary to reproduce the reported results. The data is available in the Supporting Information section.

This article has earned an open data badge “**Reproducible Research**” for making publicly available the code necessary to reproduce the reported results. The results reported in this article were reproduced partially due to computational complexity.

## Supporting information

Supporting Information

Supporting Information

## Data Availability

The data that support the findings of this study are available in the Supporting Information of this article.
