# Supplementary material for: Incompletely Observed Nonparametric Factorial Designs With Repeated Measurements: A Wild Bootstrap Approach
Source: Biom J. 2024 Nov 23;66(8):e70008. doi: 10.1002/bimj.70008 (PMC11585227; doi:10.1002/bimj.70008)
Supplement: Supplementary file 2 — Supporting Information [file BIMJ-66-e70008-s002.pdf]

Supplementary material to: Incompletely observed nonparametric factorial designs with repeated measurements: A wild bootstrap approach

Lubna Amro[1*], Frank Konietschke[2,3] and Markus Pauly[1,4]

In this supplementary material, we present the proofs of all theorems from the paper. Furthermore, we recall the definition of the different missing mechanisms and present additional type-I error and power simulation results of the asymptotic quadratic form tests and their wild bootstrap counterparts as described in Sections 3 and 4 of the paper.

1. Proofs

Proof of Theorem 3.1:
The proof is stated in Brunner et al. (1999), we will explain it here shortly: first, we know that we have convergence in distribution $\sqrt{n}\boldsymbol{C}\hat{\boldsymbol{p}} \xrightarrow{\mathrm{d}} N(\boldsymbol{0}, \boldsymbol{CVC^T})$ as $n_0 \to \infty$ under H_0. Hence, using the CMT, the quadratic form $\tilde{T}_W = n\hat{\boldsymbol{p}}^T\boldsymbol{C}^T[\boldsymbol{CVC}^T]^+\boldsymbol{C}\hat{\boldsymbol{p}}$ has asymptotically a central χ^2_f distribution with $f = rank(\boldsymbol{C})$ degrees of freedom since $\boldsymbol{V} > 0$. Moreover, as $\hat{\boldsymbol{V}}_n$ is a consistent estimator for $\boldsymbol{V}$, the result follows from Slutzky's theorem noting that the probability of the set $\{\hat{\boldsymbol{V}}_n > 0\}$ converges to 1.

Proof of Theorem 3.2:
Applying again the CMT, it follows that $tr(\boldsymbol{TV}) \cdot T_A = n\hat{\boldsymbol{p}}^T\boldsymbol{T}\hat{\boldsymbol{p}}$ has asymptotically the same distribution as $\sum_{i=1}^{a}\sum_{j=1}^{d}\zeta_{ij}B_{ij}$ (Graybill, 1976, Brunner and Puri, 2001). Then, the result follows from the invariance of the multivariate standard normal distribution under orthogonal transformations (as $\boldsymbol{C}$) and the consistency of $\hat{\boldsymbol{V}}_n$ and thus of $tr(\boldsymbol{T}\hat{\boldsymbol{V}}_n)$ by using Slutzky theorem.

Proof of Theorem 3.3:
Following similar arguments as prescribed in Friedrich and Pauly (2018), we can obtain that $(\boldsymbol{C}\hat{\mathbf{D}}_n\boldsymbol{C}^\top)^+ \xrightarrow{\mathrm{p}} (\boldsymbol{C}\mathbf{D}\boldsymbol{C}^\top)^+$ as $\hat{\boldsymbol{D}}_n = diag(\hat{\boldsymbol{V}}_n)_{ii} \xrightarrow{p} diag(\boldsymbol{V})_{ii} = \boldsymbol{D}$ where D is of full rank by assumption. Thus, the result follows from the representation theorem of quadratic forms (Rao et al., 1972).

Proof of Theorem 4.1:
First, we prove (i). We show that given the data, the expression $\sqrt{n}\hat{\boldsymbol{p}}_i^*$ is asymptotically multivariate $N(0, \kappa_i^{-1}\hat{\boldsymbol{V}}_i)$ distributed.

The results follows by applying the conditional CLT for the wild bootstrap given in Theorem

[1]Department of Statistics, TU Dortmund University, Dortmund, Germany
[2]Institute of Biometry and Clinical Epidemiology, Charitè—Universitätsmedizin Berlin, Berlin, Germany
[3]Berlin Institute of Health (BIH), Berlin, Germany
[4]UA Ruhr, Research Center Trustworthy Data Science and Security, Dortmund, Germany

Corresponding author:
Lubna Amro, Department of Statistics, TU Dortmund University, Dortmund, Germany.
Email: lubna.amro@tu-dortmund.de

A.1. in Beyersmann et al. (2013). In order to show the conditional asymptotic normality of $\sqrt{n}\hat{\boldsymbol{p}}_i^*$, we need to recall that $\hat{Y}_{ijk} = \hat{H}(X_{ijk}) = \frac{1}{N}(R_{ijk} - \frac{1}{2}), \bar{\hat{Y}}_{ij.} = \frac{1}{\lambda_{ij.}}\sum_{k=1}^{n_i}\lambda_{ijk}\hat{Y}_{ijk}$, $\hat{\boldsymbol{Y}}_{ik} = (Y_{i1k}, ..., Y_{idk})^T$, $\bar{\hat{\boldsymbol{Y}}}_{i.} = (\bar{\hat{Y}}_{i1.}, ..., \bar{\hat{Y}}_{id.})$, and $\boldsymbol{\Lambda}_{ik} = n_i diag\{\frac{\lambda_{i1k}}{\lambda_{i1.}}, ..., \frac{\lambda_{idk}}{\lambda_{id.}}\}$.

Now, it remains to show that all conditions of their Theorem A.1 are fulfilled:

A) As $1 \leq n_i/\lambda_{ij.} \leq n_i$ is bounded, $|\hat{Y}_{ijk}| \leq 1$, and $n/n_i^2 \to 0$ it follows that

$$\max_{1\leq i\leq a} \frac{\sqrt{n}||\Lambda_{ik}(\hat{\boldsymbol{Y}}_{ik} - \bar{\hat{\boldsymbol{Y}}}_{i.})||}{n_i} \xrightarrow{n\to\infty} 0 \quad \text{in probability.}$$

B) We need to study the convergence of

$$\begin{aligned} &\frac{n}{n_i^2}\sum_{k=1}^{n_i}[\Lambda_{ik}(\hat{\boldsymbol{Y}}_{ik} - \bar{\hat{\boldsymbol{Y}}}_{i.})][\Lambda_{ik}(\hat{\boldsymbol{Y}}_{ik} - \bar{\hat{\boldsymbol{Y}}}_{i.})]^T \\ &= \frac{n}{n_i}\frac{1}{n_i}\sum_{k=1}^{n_i}\Lambda_{ik}(\hat{\boldsymbol{Y}}_{ik} - \bar{\hat{\boldsymbol{Y}}}_{i.})(\hat{\boldsymbol{Y}}_{ik} - \bar{\hat{\boldsymbol{Y}}}_{i.})^T\Lambda_{ik}. \end{aligned}$$

First, we consider the diagonal elements which are given by

$$\begin{aligned} &\frac{n}{n_i}\frac{1}{n_i}\sum_{k=1}^{n_i} n_i^2\frac{\lambda_{ijk}^2}{\lambda_{ij.}^2}(\hat{Y}_{ijk} - \bar{\hat{Y}}_{ij.})^2 \\ &= \frac{n}{n_i}\frac{n_i}{\lambda_{ij.}^2}\sum_{k=1}^{n_i}\lambda_{ijk}(\hat{Y}_{ijk} - \bar{\hat{Y}}_{ij.})^2 \quad \text{(since } \lambda_{ijk}^2 = \lambda_{ijk} \text{ by definition)} \\ &= \frac{n(\lambda_{ij.} - 1)}{n_i\lambda_{ij.}}\left[\frac{n_i}{\lambda_{ij.}(\lambda_{ij.} - 1)}\sum_{k=1}^{n_i}\lambda_{ijk}(\hat{Y}_{ijk} - \bar{\hat{Y}}_{ij.})^2\right] \\ &= \frac{n(\lambda_{ij.} - 1)}{n_i\lambda_{ij.}}\left[\frac{n_i}{\lambda_{ij.}(\lambda_{ij.} - 1)}\sum_{k=1}^{n_i}\lambda_{ijk}\left(\frac{1}{N}(R_{ijk} - \frac{1}{2}) - \frac{1}{N}(\bar{R}_{ij.} - \frac{1}{2})\right)^2\right] \\ &= \frac{n(\lambda_{ij.} - 1)}{n_i\lambda_{ij.}}\left[\frac{n_i}{(N^2)\lambda_{ij.}(\lambda_{ij.} - 1)}\sum_{k=1}^{n_i}\lambda_{ijk}(R_{ijk} - \bar{R}_{ij.})^2\right] \\ &= \frac{n(\lambda_{ij.} - 1)}{n_i\lambda_{ij.}}\hat{v}_i(j,j). \end{aligned}$$

Therefore, we get (recalling that $\lambda_{ij.} \leq n_i$, $\frac{\lambda_{ij.}}{n} \to \kappa_i$, and $\hat{v}_i(j,j)$ is consistent for $v_i(j,j)$)

$$\frac{n}{n_i}\frac{1}{n_i}\sum_{k=1}^{n_i} n_i^2\frac{\lambda_{ijk}^2}{\lambda_{ij.}^2}(\hat{Y}_{ijk} - \bar{\hat{Y}}_{ij.})^2 \xrightarrow{n_0\to\infty} \frac{1}{\kappa_i}v_i(j,j) \text{ in probability.}$$

Similarly, we show it for the off-diagonal elements.

$$\frac{n}{n_i}\frac{1}{n_i}\sum_{k=1}^{n_i} n_i^2 \frac{\lambda_{ijk}\lambda_{ij'k}}{\lambda_{ij.}\lambda_{ij'.}}(\hat{Y}_{ijk}-\bar{\hat{Y}}_{ij.})(\hat{Y}_{ij'k}-\bar{\hat{Y}}_{ij'.})$$

$$= \frac{n}{n_i}\frac{n_i}{\lambda_{ij.}\lambda_{ij'.}}\sum_{k=1}^{n_i}\lambda_{ijk}\lambda_{ij'k}(\hat{Y}_{ijk}-\bar{\hat{Y}}_{ij.})(\hat{Y}_{ij'k}-\bar{\hat{Y}}_{ij'.})$$

$$= \frac{n[(\lambda_{ij.}-1)(\lambda_{ij'.}-1)+\Delta_{i,jj'}-1]}{n_i\lambda_{ij.}\lambda_{ij'.}}\left[\frac{n_i\sum_{k=1}^{n_i}\lambda_{ijk}\lambda_{ij'k}(\hat{Y}_{ijk}-\bar{\hat{Y}}_{ij.})(\hat{Y}_{ij'k}-\bar{\hat{Y}}_{ij'.})}{[(\lambda_{ij.}-1)(\lambda_{ij'.}-1)+\Delta_{i,jj'}-1]}\right]$$

$$= \frac{n[(\lambda_{ij.}-1)(\lambda_{ij'.}-1)+\Delta_{i,jj'}-1]}{n_i\lambda_{ij.}\lambda_{ij'.}}$$

$$\times\left[\frac{n_i\sum_{k=1}^{n_i}\lambda_{ijk}\lambda_{ij'k}(\frac{1}{N}(R_{ijk}-\frac{1}{2})-\frac{1}{N}(\bar{R}_{ij.}-\frac{1}{2}))(\frac{1}{N}(R_{ij'k}-\frac{1}{2})-\frac{1}{N}(\bar{R}_{ij'.}-\frac{1}{2}))}{[(\lambda_{ij.}-1)(\lambda_{ij'.}-1)+\Delta_{i,jj'}-1]}\right]$$

$$= \frac{n[(\lambda_{ij.}-1)(\lambda_{ij'.}-1)+\Delta_{i,jj'}-1]}{n_i\lambda_{ij.}\lambda_{ij'.}}\left[\frac{n_i\sum_{k=1}^{n_i}\lambda_{ijk}\lambda_{ij'k}(R_{ijk}-\bar{R}_{ij.})(R_{ij'k}-\bar{R}_{ij'.})}{(N^2)[(\lambda_{ij.}-1)(\lambda_{ij'.}-1)+\Delta_{i,jj'}-1]}\right]$$

$$= \frac{n[(\lambda_{ij.}-1)(\lambda_{ij'.}-1)+\Delta_{i,jj'}-1]}{n_i\lambda_{ij.}\lambda_{ij'.}}\hat{v}_i(j,j').$$

As $\Delta_{i,jj'} \le n_i, \lambda_{ij.} \le n_i$, $\frac{\lambda_{ij.}}{n} \to \kappa_i$, and $\hat{v}_i(j,j')$ is consistent for $v_i(j,j')$ it follows that

$$\frac{n}{n_i}\frac{1}{n_i}\sum_{k=1}^{n_i} n_i^2 \frac{\lambda_{ijk}\lambda_{ij'k}}{\lambda_{ij.}\lambda_{ij'.}}(\hat{Y}_{ijk}-\bar{\hat{Y}}_{ij.})(\hat{Y}_{ij'k}-\bar{\hat{Y}}_{ij'.}) \xrightarrow{n_0\to\infty} \frac{1}{\kappa_i}v_i(j,j') \text{ in probability.}$$

So, summing up, we get that

$$\frac{n}{n_i}\frac{1}{n_i}\sum_{k=1}^{n_i}\Lambda_{ik}(\hat{\boldsymbol{Y}}_{ik}-\bar{\hat{\boldsymbol{Y}}}_{i.})(\hat{\boldsymbol{Y}}_{ik}-\bar{\hat{\boldsymbol{Y}}}_{i.})^T\Lambda_{ik} \xrightarrow{n_0\to\infty} \frac{1}{\kappa_i}\boldsymbol{V}_i \text{ in probability.}$$

Thus, we have conditional weak convergence given the data $\boldsymbol{X}$

$$\frac{\sqrt{n}}{n_i}\sum_{k=1}^{n_i} W_{ik}\Lambda_{ik}(\hat{\boldsymbol{Y}}_{ik}-\bar{\hat{\boldsymbol{Y}}}_{i.}) \xrightarrow{d} N(0,\frac{1}{\kappa_i}\boldsymbol{V}_i),$$

in probability and therefore $\sqrt{n}\hat{\boldsymbol{p}}_i^*$ is asymptotically multivariate normally distributed with expectation 0 and asymptotic covariance matrix $\frac{1}{\kappa_i}\boldsymbol{V}_i$. Finally, a point-wise application of Slutzky shows that $\sqrt{n}\hat{\boldsymbol{p}}^*$ converges in distribution to $N(0,\bigoplus_{i=1}^{a}\kappa_i^{-1}\boldsymbol{V}_i)$ in probability given the data.

Proof of Theorem 4.2:

It has already been shown that $\hat{\boldsymbol{V}}_n - \boldsymbol{V} \xrightarrow{p} 0$ (Brunner et al., 1999, Theorem 3.5). So, it suffices to show that $\hat{\boldsymbol{V}}_n^* - \hat{\boldsymbol{V}} \xrightarrow{p} 0$. Thus, consider

$$\hat{p}_{ij}^* = \frac{1}{N}\bar{Z}_{ij.}^* = \frac{1}{\lambda_{ij.}}\sum_{k=1}^{n_i}\lambda_{ijk}\frac{1}{N}(W_{ik}Z_{ijk}) = \frac{1}{\lambda_{ij.}}\sum_{k=1}^{n_i}\lambda_{ijk}\frac{1}{N}(W_{ik}(R_{ijk}-\bar{R}_{ij.})).$$

Then,

$$\mathbb{E}\Big(\hat{p}^*_{ij}|\boldsymbol{X}\Big) = \mathbb{E}\Big(\frac{1}{\lambda_{ij.}}\sum_{k=1}^{n_i} W_{ik}\lambda_{ijk}\frac{1}{N}Z_{ijk}|\boldsymbol{X}\Big) = \frac{1}{N\lambda_{ij.}}\sum_{k=1}^{n_i}\mathbb{E}\Big(W_{ik}\Big).\lambda_{ijk}Z_{ijk} = 0.$$

And, as $\lambda_{ij.}/n \to \kappa_i$

$$\begin{aligned} Var\Big(\hat{p}^*_{ij}|\boldsymbol{X}\Big) &= \frac{1}{(N\lambda_{ij.})^2}\sum_{k=1}^{n_i} Var\Big(W_{ik}\Big)\Big(\lambda_{ijk}Z_{ijk}\Big)^2 \quad (W_{ik}Z_{ijk} \text{ are conditionally independent given } \boldsymbol{X}.) \\ &= \frac{1}{(N\lambda_{ij.})^2}\sum_{k=1}^{n_i}\lambda_{ijk}\Big(Z_{ijk}\Big)^2 \\ &\leq \frac{1}{N^2\lambda^2_{ij.}} n_i(N-1)^2 \leq \frac{n}{\lambda^2_{ij.}}(\frac{N-1}{N})^2 \xrightarrow{p} 0. \end{aligned}$$

Consequently, by using Chebyshev's inequality, we get $\hat{p}^*_{ij} \to 0$ in probability for all $i = 1, ..., a$. Now, we show by tedious calculations that the diagonal elements

$$\begin{aligned} \hat{v}_i(j,j) - \hat{v}^*_i(j,j) &= \frac{n_i\sum_{k=1}^{n_i}\lambda_{ijk}[R_{ijk}-\bar{R}_{ij.}]^2}{(N^2)\lambda_{ij.}(\lambda_{ij.}-1)} - \frac{n_i\sum_{k=1}^{n_i}\lambda_{ijk}[Z^*_{ijk}-\bar{Z}^*_{ij.}]^2}{(N^2)\lambda_{ij.}(\lambda_{ij.}-1)} \\ &= \frac{n_i}{(N^2)\lambda_{ij.}(\lambda_{ij.}-1)}\sum_{k=1}^{n_i}\lambda_{ijk}\Big[Z^2_{ijk} - [Z^{*2}_{ijk} - 2Z^*_{ijk}\bar{Z}^*_{ij.} + \bar{Z}^{*2}_{ij.}]\Big] \\ &= \frac{n_i}{(N^2)\lambda_{ij.}(\lambda_{ij.}-1)}\sum_{k=1}^{n_i}\lambda_{ijk}\Big[2Z^*_{ijk}\bar{Z}^*_{ij.} - \bar{Z}^{*2}_{ij.}\Big] \quad (\text{since } W^2_{ik}=1) \\ &= \frac{n_i}{(N^2)\lambda_{ij.}(\lambda_{ij.}-1)}\Big[2\bar{Z}^*_{ij.}\sum_{k=1}^{n_i}\lambda_{ijk}Z^*_{ijk} - \lambda_{ij.}\bar{Z}^{*2}_{ij.}\Big] \\ &= \frac{n_i}{(N^2)\lambda_{ij.}(\lambda_{ij.}-1)}\lambda_{ij.}\bar{Z}^{*2}_{ij.} \\ &= \frac{n_i}{\lambda_{ij.}-1}\frac{\bar{Z}^*_{ij.}}{N}\frac{\bar{Z}^*_{ij.}}{N} \\ &= \frac{n_i}{\lambda_{ij.}-1}\hat{p}^*_{ij}\hat{p}^*_{ij} \\ &\xrightarrow{n_0\to\infty} 0 \text{ in probability} \quad (\hat{p}^*_{ij} \xrightarrow{p} 0,\ \frac{\lambda_{ij.}}{n} \to \kappa_i,\ n_i \leq n). \end{aligned}$$

Furthermore, using similar arguments as above, we can show that an analogous result holds for the off-diagonal elements. And, thus $\hat{\boldsymbol{V}}^*_n - \hat{\boldsymbol{V}} \xrightarrow{p} 0$. Following the same steps as in the proofs of Theorems 3.1-3.3 , this concludes the proof.

2. Missing Data Mechanism

Rubin (1976) identified three missing mechanisms for the data based on the relationship between the missing values and observed values. Let $\boldsymbol{X}$ denote a data set which can be decomposed into observed and unobserved portions $\boldsymbol{X} = (\boldsymbol{X}_{obs}, \boldsymbol{X}_{mis})$. Let $\boldsymbol{\lambda}$ be a binary matrix whose components indicate whether $\boldsymbol{X}$ is observed or missing. The three missing data mechanisms are:

(1) **Missing completely at random (MCAR):**
The probability of an observation being missing does not depend on the values of any observed or unobserved data, i.e., $P(\boldsymbol{\lambda}|\boldsymbol{X}_{obs}, \boldsymbol{X}_{mis}) = P(\boldsymbol{\lambda})$. This implies that the conditional and marginal distributions can always be accurately estimated from the observed data.

(2) **Missing at random (MAR):**
The probability of the missingness can depend on the observed data but not on the unobserved data, i.e. $P(\boldsymbol{\lambda}|\boldsymbol{X}_{obs}, \boldsymbol{X}_{mis}) = P(\boldsymbol{\lambda}|\boldsymbol{X}_{obs})$. Thus, the missing data is due to an external effect, not the variable itself. Note that MCAR is a special case of MAR.

(3) **Missing not at random (MNAR):**
The probability of the missingness can depend on the unobserved data, i.e. $P(\boldsymbol{\lambda}|\boldsymbol{X}_{obs}, \boldsymbol{X}_{mis}) \neq P(\boldsymbol{\lambda}|\boldsymbol{X}_{obs})$. MNAR is also known as the non-ignorable case (Little and Rubin, 2019) since the missing observation is dependent on the outcome result.

For additional information on the various missing mechanisms, we refer to Little and Rubin (2019).

3. Type-I Error and Power Results

In the sequel, we present some additional type-I error and power results of the Monte Carlo simulation study, that is described in detail in Section 5 of the paper, for testing the hypothesis of no group effect H_0^G, no time effect H_0^T, as well as no interaction effect H_0^{GT} for incomplete nonparametric factorial designs with repeated measurements under the MCAR, and MAR schemes. The type-I error results under MCAR scheme, $d = 4$ time points for the hypotheses H_0^T and H_0^{GT} are presented in Tables 1, 2, 3, and 4 for the normal, double exponential, lognormal and chi-square distribution, respectively. The results for $d = 8$ time points are in Tables 5, 6, 7, and 8 for the normal, double exponential, lognormal and chi-square distribution, respectively. The results for the hypothesis H_0^G are presented in Tables 9 - 12. Moreover, the type-I error results under MAR scheme, for the hypotheses H_0^T and H_0^{GT} for symmetric and skewed distributions are presented in Tables 13 - 16 ($d = 4$ time points) and Tables 17 - 20 ($d = 8$). The results for the hypothesis H_0^G are in Tables 21, 22, 23, and 24 for the normal, double exponential, chi-square and lognormal distribution, respectively.

Simulation results for the type-I error rate under similar attributes to the fluvoxamine trial data and the skin disorder trial data for sample sizes ($n = 99; d = 3$) and ($n_1 = 88, n_2 = 84; d = 3$) are presented in Tables 25 and 26, respectively.

Further, type-I error control results for detecting the effect of increasing missing rates under MCAR covering missingness in observations ranging from 10% to 60% are summarized in Figures 1 and 2 for the double exponential and lognormal distribution, respectively.

Furthermore, type-I error results for increasing the number of groups for the hypothesis H_0^{GT} are presented in Figure 3.

Moreover, the type-I error results for ordinal data under MCAR framework for $d = 8$ time points are presented in Figure 4. And, the results for ordinal data under MAR framework are summarized in Figure 5 ($d = 4$ time points) and Figure 6 ($d = 8$).

Power analysis results of the considered methods under the MCAR framework for several distributions, involving various covariance settings for detecting Alternative 1 (as stated in the paper) are summarized in Figures 7 - 8 (missing rate $r = 10\%$). The power results for investigating Alternative 2 are displayed in Figures 9 - 10 (missing rate $r = 10\%$) and Figures 11 - 12 ($r = 30\%$). The power analysis results under the MAR framework for detecting Alternative 1

and 2 are summarized in Figures 13 - 16 (MAR1 scenario) and Figures 17 - 20 (MAR2 scenario).

Lastly, additional simulation results comparing our methods with alternative approaches proposed by Rubarth et al. (2022a) are presented in Tables 27 - 30. Additionally, simulation results comparing our methods with those of Rubarth et al. (2022b) are provided in Tables 31 - 38.

Table 1. Simulation results for type-I error level ($\alpha = 0.05$) for normal distribution, $d = 4$ and different percentages r in case of the MCAR mechanism.

			$r = 10$					$r = 30$				
Hyp	Cov	n	Classic		WBtstrp			Classic		WBtstrp		
			T_W	T_A	T_W^W	T_A^W	T_M^W	T_W	T_A	T_W^W	T_A^W	T_M^W
H_0^T	AR	(5,5)	26	7.4	5	5.2	5.9	32.8	8.2	5.7	5.1	6.3
		(10,10)	12.2	6	5.2	5.5	5.8	14.5	6.6	5.3	5.6	6.1
		(10,20)	12.2	6.2	5.8	5.6	6	14	6.6	6	5.8	6.4
		(20,20)	8	5	4.9	4.9	5.1	8.4	5.3	4.8	4.9	4.9
	CS	(5,5)	26.3	7	5	4.9	6.2	33	8	5.6	4.8	5.8
		(10,10)	12.6	5.7	5.3	5.3	5.5	14.8	6.1	5.4	5.1	5.7
		(10,20)	12.4	5.8	5.9	5.3	5.7	13.8	6.1	6	5.4	6.2
		(20,20)	8	4.9	5	4.9	5	8.4	4.8	5	4.6	5
	TP	(5,5)	26.1	7.7	5	5.3	6.1	32.2	8.8	5.7	5.3	6.3
		(10,10)	11.9	6.2	5.3	5.4	5.7	14.7	6.9	5.2	5.8	6.3
		(10,20)	11.9	6.5	5.5	5.7	6	13.7	6.9	6	6	6.7
		(20,20)	7.8	5.1	4.9	4.9	5	8.4	5.4	4.8	5	5
H_0^{GT}	AR	(5,5)	25.6	6.4	5.2	4.6	5.8	32.8	7.9	6.1	4.9	6.2
		(10,10)	12	5.6	4.9	5.1	5.2	15.2	6.5	5.7	5.6	6.2
		(10,20)	12.1	6.1	5.8	5.5	5.9	14.5	6.2	6.2	5.4	6.3
		(20,20)	7.8	4.9	4.9	4.8	4.6	9.1	5.4	5.6	5	5.5
	CS	(5,5)	25.5	6.4	5	4.3	5.9	33.5	8	5.7	4.7	6.2
		(10,10)	11.8	5.3	4.9	4.9	4.9	15	6.7	5.8	5.6	6.4
		(10,20)	12.3	5.8	6	5.5	6	14.4	6.5	6.3	5.6	6.5
		(20,20)	7.9	4.9	5	4.8	4.8	8.7	5.5	5.2	5.2	5.2
	TP	(5,5)	26	7	5.2	4.9	5.6	32.9	8.6	5.9	5.4	6.3
		(10,10)	12.1	5.8	4.9	5.2	5.4	15.3	6.6	5.4	5.4	6
		(10,20)	12.2	6.5	6	5.7	6	14.4	6.6	6.3	5.7	6.2
		(20,20)	7.8	5	4.9	4.9	4.8	9	5.7	5.7	5.2	5.5

Table 2. Simulation results for type-I error level ($\alpha = 0.05$) for double exponential distribution, $d = 4$ and different percentages r in case of the MCAR mechanism.

			$r = 10$					$r = 30$				
Hyp	Cov	n	Classic		WBtstrp			Classic		WBtstrp		
			T_W	T_A	T_W^W	T_A^W	T_M^W	T_W	T_A	T_W^W	T_A^W	T_M^W
H_0^T	AR	(5,5)	25.6	7.3	5	5	5.9	32.7	8.3	5.8	5.1	6.2
		(10,10)	12.1	6	5.3	5.4	5.8	14.5	6.5	5.2	5.7	6
		(10,20)	11.9	5.9	5.6	5.4	5.8	14	6.6	6	5.8	6.5
		(20,20)	7.9	5.1	5	5	5.1	8.4	5.1	4.9	4.9	5
	CS	(5,5)	26.1	6.8	4.6	5	6	33.1	8.1	5.8	4.8	6
		(10,10)	12.3	5.5	5.2	5.2	5.5	14.5	6.4	5.2	5.2	5.6
		(10,20)	12.3	5.9	5.9	5.2	5.8	13.5	6.1	5.8	5.3	6
		(20,20)	7.9	5	4.8	4.9	5.1	8.4	5	4.9	4.7	4.9
	TP	(5,5)	25.7	7.7	5	5.2	5.9	32.4	8.5	5.8	5.4	6.2
		(10,10)	11.9	6.1	5.4	5.3	5.7	14.5	6.9	5.3	5.9	6.2
		(10,20)	11.6	6.1	5.3	5.5	5.8	13.9	6.9	6.1	6	6.7
		(20,20)	7.6	5.2	5	5.1	5.2	8.3	5.1	4.9	4.8	5.1
H_0^{GT}	AR	(5,5)	25.1	6.6	5	4.6	5.8	32.5	8	6.1	4.9	6.2
		(10,10)	11.8	5.5	5	5.1	5.1	15.2	6.4	5.5	5.4	6
		(10,20)	11.9	5.8	5.9	5.4	5.8	14.3	6.1	6	5.4	6.4
		(20,20)	8.1	5.1	5.1	4.9	5	8.8	5.6	5.3	5.1	5.5
	CS	(5,5)	25.6	6.3	5.2	4.4	5.6	33.2	7.8	6.1	4.6	6.2
		(10,10)	11.9	5.2	5	4.8	4.9	15	6.7	5.8	5.7	6.3
		(10,20)	12.1	5.8	5.9	5.3	6	14	6.1	6.2	5.4	6.5
		(20,20)	8.1	4.9	5	4.8	4.9	8.7	5.2	5.2	4.8	5.2
	TP	(5,5)	25.7	7	5.2	4.9	5.8	32.3	8.8	6.1	5.4	6.5
		(10,10)	11.7	5.8	4.9	5.2	5.3	15.1	6.6	5.4	5.4	5.9
		(10,20)	12	6.3	5.9	5.8	6	14.2	6.4	6.2	5.6	6.3
		(20,20)	8	5.1	4.9	4.9	4.9	8.8	5.6	5.4	5.2	5.4

Table 3. Simulation results for type-I error level ($\alpha = 0.05$) for chi-square distribution, $d = 4$ and different percentages r in case of the MCAR mechanism.

			$r = 10$					$r = 30$				
Hyp	Cov	n	Classic		WBtstrp			Classic		WBtstrp		
			T_W	T_A	T_W^W	T_A^W	T_M^W	T_W	T_A	T_W^W	T_A^W	T_M^W
H_0^T	AR	(5,5)	26.2	7.1	5	5.1	6	32.4	8.2	6	4.9	6.2
		(10,10)	12.3	5.8	5.3	5.3	5.5	14.4	6.6	5.3	5.6	6.2
		(10,20)	12.1	6.2	5.8	5.7	6	13.7	6.6	6	5.8	6.3
		(20,20)	7.9	5	5	4.8	4.9	8.5	5.2	4.9	4.9	4.9
	CS	(5,5)	26.2	7	5	5.1	6	33.2	8	5.6	4.6	6
		(10,10)	12.4	5.6	5.2	5.2	5.5	14.6	6.1	5.5	5.1	5.8
		(10,20)	12.5	5.8	6	5.4	5.8	13.5	6	6	5.3	6
		(20,20)	8.1	5	5	4.9	5	8.6	5	5.1	4.7	5.1
	TP	(5,5)	25.7	7.4	5	5.3	6.2	31.9	8.6	5.6	5.3	6.3
		(10,10)	12.2	6.2	5.3	5.4	5.7	14.5	6.8	5.2	5.7	6.2
		(10,20)	12	6.3	5.7	5.7	6	13.8	6.9	6	6.2	6.7
		(20,20)	7.5	5	4.7	4.9	4.9	8.2	5.2	4.9	4.9	5.1
H_0^{GT}	AR	(5,5)	25.7	6.4	5.3	4.5	5.7	32.4	8	6.1	4.9	6.2
		(10,10)	11.7	5.6	5	5.1	5	14.6	6.4	5.5	5.5	6.1
		(10,20)	11.8	6.2	6.1	5.6	6.1	14	6.2	6	5.3	6.2
		(20,20)	8.2	4.9	5.2	4.8	4.9	8.7	5.5	5.3	5.2	5.4
	CS	(5,5)	25.4	6.3	5.2	4.4	5.7	33	8.1	5.8	4.7	6.3
		(10,10)	12.1	5.3	5	4.8	5.1	14.7	6.4	5.7	5.6	6
		(10,20)	12.2	6	5.9	5.6	5.8	14.1	6.3	6.2	5.7	6.4
		(20,20)	7.9	5	5.1	4.9	5	8.7	5.5	5.2	5.1	5.3
	TP	(5,5)	25.7	6.9	5.4	4.7	5.5	31.9	8.5	6	5.3	6.1
		(10,10)	11.8	5.9	5	5.2	5.2	14.9	6.6	5.4	5.6	6
		(10,20)	12	6.3	6	5.7	6	14	6.3	6.1	5.5	6.1
		(20,20)	8.1	5.2	4.9	5	4.8	8.9	5.6	5.2	5.2	5.3

Table 4. Simulation results for type-I error level ($\alpha = 0.05$) for lognormal distribution, $d = 4$ and different percentages r of MCAR data .

Hyp	Cov	n	$r = 10$					$r = 30$				
			Classic		WBtstrp			Classic		WBtstrp		
			T_W	T_A	T_W^W	T_A^W	T_M^W	T_W	T_A	T_W^W	T_A^W	T_M^W
H_0^T	AR	(5,5)	26.5	6.6	5	4.8	5.6	31.6	8.3	5.5	5.3	6.1
		(10,10)	12.7	5.5	5.6	5.1	5.5	14.8	6.2	5.3	5.3	6
		(10,20)	12.4	6.2	6	5.5	6	13.5	6.2	5.8	5.5	6.1
		(20,20)	8.2	5	5.1	5	5.1	8.9	5	5.2	4.6	5
	CS	(5,5)	26.6	6.6	5.4	4.6	5.8	31.8	7.8	5.6	4.5	5.4
		(10,10)	12.7	5.4	5.6	4.8	5.4	14.9	6	5.5	5.2	5.7
		(10,20)	12.7	5.7	6.3	5.3	6	13.6	5.7	5.7	5.1	5.7
		(20,20)	8.3	5.2	5.3	5.1	5.3	8.9	5	5.4	4.8	5.2
	TP	(5,5)	26.5	7	5.2	5.3	5.8	31.2	8.4	5.6	5.6	6.3
		(10,10)	13	5.8	5.8	5.1	5.5	15	6.5	5.4	5.6	6.2
		(10,20)	12.4	6.3	6.3	5.9	6.1	14.1	6.6	6.1	5.8	6.7
		(20,20)	8.3	5.1	5.4	5	5.1	9.2	5.2	5.2	5	5.1
H_0^{GT}	AR	(5,5)	25.6	6.3	4.7	4.5	5.4	31.9	7.9	5.4	4.8	6.2
		(10,10)	11.8	5.1	4.8	4.5	4.9	14.6	6.1	5.1	5.2	5.8
		(10,20)	11.8	5.8	5.8	5.4	5.9	13.1	5.7	5.4	5	5.7
		(20,20)	8.2	5.2	5.1	5	5.1	8.6	5.2	5	5	5.3
	CS	(5,5)	25.4	6.3	4.8	4.5	5.7	32.5	8	5.4	4.7	6
		(10,10)	12	5.2	4.8	4.8	5	14.6	6.2	5.2	5	5.7
		(10,20)	11.8	5.7	5.6	5.2	5.9	13.2	5.7	5.8	5.1	6
		(20,20)	8	5.3	5	5.2	5.3	8.6	5.4	5.1	5.2	5.3
	TP	(5,5)	25.1	6.5	4.9	4.5	5.5	31.4	8.2	5.7	5.1	6.2
		(10,10)	11.7	5.6	4.6	5.1	5.2	14.2	6.4	5.1	5.3	6
		(10,20)	11.6	5.8	5.8	5.3	5.8	13.2	5.8	5.7	5.3	6
		(20,20)	8	5.2	5.2	5.1	5.1	8.6	5.3	4.9	5	5.1

Table 5. Simulation results for type-I error level ($\alpha = 0.05$) for normal distribution, $d = 8$ and different percentages r in case of the MCAR mechanism.

			$r = 10$					$r = 30$				
Hyp	Cov	n	Classic		WBtstrp			Classic		WBtstrp		
			T_W	T_A	T_W^W	T_A^W	T_M^W	T_W	T_A	T_W^W	T_A^W	T_M^W
H_0^T	AR	(5,5)	45.7	5	6.2	4.7	6	36	5.2	4.9	4.5	5.5
		(10,10)	34.7	5	4.9	5.2	5.4	42.6	5.1	4.9	5.4	6.3
		(10,20)	30.6	5.3	6	5.7	6	35.6	5.1	6.8	5.4	6.6
		(20,20)	15.6	4.8	5.3	5	5	16.9	4.9	5.1	5.2	5.1
	CS	(5,5)	50.4	3.7	7	4.2	5.3	37.4	4.1	5.1	3.8	5.4
		(10,10)	34.3	3.9	5.1	4.7	5.2	41.8	4.6	5	5.3	6.1
		(10,20)	30.3	4.5	6.3	5.3	5.9	35.7	4.5	6.7	5.4	6.4
		(20,20)	15.5	4.3	5.2	4.9	4.8	17.1	4.4	5.1	5	5.4
	TP	(5,5)	42.2	6.7	5.8	5.6	6.1	34.2	6.6	4.4	5.3	5.8
		(10,10)	34.5	5.8	4.9	5.6	5.6	43.2	5.9	4.5	5.8	6.1
		(10,20)	29.7	6.2	6.1	5.9	6	36	5.7	6.7	5.7	6.4
		(20,20)	15.2	5.4	5.1	5.4	5.3	16.8	5.3	4.9	5.3	5.2
H_0^{GT}	AR	(5,5)	46.6	5.2	6.4	5	6.2	36.3	5.3	5.1	4.5	6
		(10,10)	33.7	5.2	5.1	5.4	5.8	42	4.9	4.3	5.2	5.8
		(10,20)	30	4.7	6.1	5.2	5.5	35.4	5.1	6.8	5.6	6.5
		(20,20)	14.6	4.8	4.9	4.9	5.1	16.6	4.8	5.1	5.1	5.2
	CS	(5,5)	50.6	3.5	6.6	4	5.6	37.6	4	5.3	3.6	5.8
		(10,10)	33.7	4.3	5.3	5.2	5.6	40.9	4.3	4.6	5	5.9
		(10,20)	30.1	4	6	5.1	5.7	35	4.4	7.2	5.2	6.4
		(20,20)	14.9	4.2	5	4.8	4.7	17.1	4.4	4.9	4.9	4.8
	TP	(5,5)	42.1	7.2	5.9	6	6.4	34	6.8	5	5.3	6.1
		(10,10)	34.5	5.8	4.9	5.4	5.6	43.4	6.2	4.6	5.9	6
		(10,20)	29.6	5.9	5.9	5.5	5.6	36	6.1	6.8	6	6.9
		(20,20)	14.7	5.1	5	4.9	5	17.1	5.3	5.2	5.4	5.3

Table 6. Simulation results for type-I error level ($\alpha = 0.05$) for double exponential distribution, $d = 8$ and different percentages r in case of the MCAR mechanism.

			$r=10$					$r=30$				
Hyp	Cov	n	Classic		WBtstrp			Classic		WBtstrp		
			T_W	T_A	T_W^W	T_A^W	T_M^W	T_W	T_A	T_W^W	T_A^W	T_M^W
H_0^T	AR	(5,5)	45	5	5.7	4.8	5.8	35.2	5.2	4.8	4.6	5.5
		(10,10)	34.7	4.8	4.8	5.2	5.3	42.9	5.2	4.6	5.4	6
		(10,20)	30.1	5.3	6	5.7	6	35.4	5	6.7	5.4	6.3
		(20,20)	15.5	4.9	5.2	5	5.1	16.8	5	5.2	5.2	5.3
	CS	(5,5)	49.7	3.5	6.5	4.1	5.2	36.9	4.2	5.1	3.9	5.5
		(10,10)	34.2	3.9	4.9	4.7	5.1	41.9	4.4	4.9	5.2	6
		(10,20)	30.2	4.4	6.2	5.4	5.7	35.4	4.4	6.7	5.2	6.6
		(20,20)	15.2	4.3	5.2	4.9	4.9	16.8	4.3	5.1	5	5.3
	TP	(5,5)	42.6	6.5	5.9	5.4	6	34	6.3	4.8	5.2	5.7
		(10,10)	34.4	5.8	4.9	5.7	5.6	43.2	5.8	4.6	5.6	6.1
		(10,20)	29.6	6.2	6.1	6	6.2	35.9	5.7	6.8	5.6	6.5
		(20,20)	15.3	5.6	5.4	5.4	5.4	16.7	5.2	5.2	5.1	5.3
H_0^{GT}	AR	(5,5)	45.3	5.2	6	5	5.9	35	5.2	5	4.5	5.8
		(10,10)	33.7	5.2	4.7	5.5	5.8	42.2	5	4.2	5.3	5.9
		(10,20)	29.4	4.8	6.1	5.2	5.5	35.3	5.2	6.6	5.8	6.6
		(20,20)	14.8	4.8	5	4.9	5	16.6	4.8	5.1	5.2	5.1
	CS	(5,5)	49.9	3.4	6.8	4	5.7	37.7	4.1	5.3	3.8	5.8
		(10,10)	33.7	4.3	5.1	5.2	5.7	40.7	4.2	4.5	5.1	5.7
		(10,20)	30.1	4	6	5	5.6	34.9	4.4	7	5	6.7
		(20,20)	14.9	4.2	4.8	4.7	4.7	16.9	4.4	4.8	4.8	5
	TP	(5,5)	42.1	7.1	5.7	5.8	6.5	33.3	6.6	4.9	5.1	6.2
		(10,10)	34.4	5.9	5	5.5	5.7	43	6.1	4.5	5.9	6.1
		(10,20)	29.1	5.8	5.9	5.6	5.6	35.8	6.1	6.5	6.2	6.9
		(20,20)	14.6	5	5.1	4.8	5	17	5.3	5.2	5.3	5.4

Table 7. Simulation results for type-I error level ($\alpha = 0.05$) for chi-square distribution, $d = 8$ and different percentages r in case of the MCAR mechanism.

			$r = 10$					$r = 30$				
Hyp	Cov	n	Classic		WBtstrp			Classic		WBtstrp		
			T_W	T_A	T_W^W	T_A^W	T_M^W	T_W	T_A	T_W^W	T_A^W	T_M^W
H_0^T	AR	(5,5)	45.5	4.8	6.1	4.6	6	35.9	5.1	4.8	4.4	5.5
		(10,10)	34.9	4.9	5	5.2	5.3	42.3	5.1	4.9	5.4	6.2
		(10,20)	30	5.3	6.1	5.7	6	35.6	5.2	6.8	5.5	6.7
		(20,20)	15.2	4.8	5.3	5.2	5.1	17	4.9	5	5.3	5.2
	CS	(5,5)	49.8	3.7	6.7	4.3	5.6	37	4.1	4.9	3.9	5.1
		(10,10)	34.4	3.9	5	4.7	5.3	41.5	4.4	5.1	5.2	5.9
		(10,20)	30	4.4	6	5.5	5.8	35.7	4.5	6.8	5.5	6.9
		(20,20)	14.9	4.2	5.2	4.8	4.9	16.7	4.4	5.1	5	5.3
	TP	(5,5)	41.8	6.5	5.6	5.6	6.2	33.3	6.4	4.5	5.2	5.5
		(10,10)	35	5.8	4.9	5.6	5.6	43.4	5.8	4.4	5.7	6
		(10,20)	29.3	6.2	6.2	6	6.1	35.5	5.7	6.5	5.7	6.5
		(20,20)	15.1	5.5	5.3	5.3	5.3	17	5.2	5.1	5.2	5.4
H_0^{GT}	AR	(5,5)	45.7	5	6.1	4.7	6.2	35.9	5	5.2	4.3	5.9
		(10,10)	33.8	5.2	4.9	5.5	5.7	42.3	4.9	4.4	5.2	5.9
		(10,20)	29.6	4.8	5.8	5.2	5.6	35.1	4.9	6.6	5.4	6.3
		(20,20)	14.4	4.7	5	4.9	5	16.6	4.8	5.2	5.2	5.1
	CS	(5,5)	50.1	3.3	6.7	3.8	5.9	37.5	4.1	5	3.7	5.8
		(10,10)	34.1	4.3	5.2	5.2	5.7	41.1	4.3	4.5	4.9	5.7
		(10,20)	30	4.1	6	5	5.8	34.7	4.4	7	5.2	6.4
		(20,20)	14.7	4.1	4.9	4.8	4.9	16.9	4.3	5.1	4.8	5
	TP	(5,5)	41.1	7.1	5.8	5.9	6.6	33.9	6.8	4.4	5.3	6
		(10,10)	34.4	5.9	4.7	5.5	5.7	43.3	5.9	4.4	6	6.2
		(10,20)	29.1	5.6	5.7	5.5	5.8	35.5	5.9	6.4	5.9	6.5
		(20,20)	14.6	5.1	5.1	5	5.2	16.7	5.2	5.2	5.4	5.3

Table 8. Simulation results for type-I error level ($\alpha = 0.05$) for lognormal distribution, $d = 8$ and different percentages r in case of the MCAR mechanism.

Hyp	Cov	n	$r = 10$					$r = 30$				
			Classic		WBtstrp			Classic		WBtstrp		
			T_W	T_A	T_W^W	T_A^W	T_M^W	T_W	T_A	T_W^W	T_A^W	T_M^W
H_0^T	AR	(5,5)	44.3	4.3	5.8	4.4	5.5	35.2	4.5	4.6	4	5.1
		(10,10)	35.1	4.4	4.9	4.9	5	42.6	4.8	4.7	5.3	6.1
		(10,20)	28.8	4.7	5.9	5.4	5.7	34.2	5.1	6.2	5.6	6.5
		(20,20)	14.7	4.6	5.3	5	5	16.7	4.7	5.3	5.1	5.3
	CS	(5,5)	47.4	3.2	6.4	4	5.1	34.8	3.9	5	3.8	4.5
		(10,10)	34.2	3.7	4.8	4.5	4.8	42.1	4	4.9	4.9	5.5
		(10,20)	29.2	3.7	5.9	4.8	5.4	34.7	4.1	6.5	5.1	6.5
		(20,20)	14.9	4.3	5.3	5.2	5.3	16.6	4	5.2	4.6	5
	TP	(5,5)	41.6	6	5.6	5.3	5.9	33	5.8	4.3	4.8	5.4
		(10,10)	36.5	5.6	5.2	5.6	5.5	44.1	5.5	4.9	5.4	5.8
		(10,20)	30.1	5.9	6.6	5.9	6	35	5.6	6.3	5.8	6.5
		(20,20)	16.4	5.6	6.1	5.5	5.6	17.6	5.2	5.8	5.4	5.5
H_0^{GT}	AR	(5,5)	43.5	4.7	5.6	4.7	5.9	35.4	4.5	4.4	3.9	5.4
		(10,10)	34	4.7	4.4	5.3	5.5	41.6	4.8	4.5	5.3	5.7
		(10,20)	28.1	4.6	5.3	5.1	5.5	33.9	4.4	5.8	4.9	6
		(20,20)	14.4	4.6	4.9	5	5	16.3	4.6	4.9	5	5.1
	CS	(5,5)	47.7	3.1	6.1	3.7	5.5	35.4	3.8	4.6	3.7	5.1
		(10,10)	33.2	4	4.9	5.1	5.6	40.6	4.2	4.6	4.9	5.5
		(10,20)	27.9	3.8	5.6	4.9	5.6	33.9	4	6.2	5	6.2
		(20,20)	14.8	3.9	4.7	4.6	4.7	16.3	4.1	4.8	4.7	4.7
	TP	(5,5)	40.4	6.6	4.8	5.7	6.4	33.4	6	4.6	4.8	5.8
		(10,10)	34.3	5.4	4.2	5.4	5.5	42.9	5.6	4.4	5.8	6.3
		(10,20)	27.9	5.3	5.4	5.3	5.6	33.8	5.5	5.5	5.7	6.4
		(20,20)	14.2	5.1	4.7	5.2	5.3	16.6	5.3	5.1	5.6	5.5

Table 9. *Simulation results for type-I error level ($\alpha = 0.05$) for normal distribution, testing the hypothesis H_0^G, $d \in \{4, 8\}$ and different percentages r in case of the MCAR mechanism.*

d	Cov	n	$r = 10$					$r = 30$				
			Classic		WBtstrp			Classic		WBtstrp		
			T_W	T_A	T_W^W	T_A^W	T_M^W	T_W	T_A	T_W^W	T_A^W	T_M^W
4	AR	(5,5)	8.6	8.6	3.7	3.7	5.1	9.7	9.7	4.5	4.5	4.8
		(10,10)	6	6	4.3	4.3	5.1	7.2	7.2	5	5	5.2
		(10,20)	5.8	5.8	4.6	4.6	5.2	6.4	6.4	4.9	4.9	5.3
		(20,20)	5.3	5.3	5	5	5	5.4	5.4	4.7	4.7	4.8
	CS	(5,5)	8.7	8.7	3.6	3.6	5.2	9.6	9.6	4.4	4.4	5
		(10,10)	5.8	5.8	4.2	4.2	4.9	7	7	4.9	4.9	5.3
		(10,20)	5.8	5.8	4.5	4.5	5.1	6.2	6.2	4.7	4.7	5.1
		(20,20)	5.2	5.2	4.7	4.7	5	5.6	5.6	5	5	5
	TP	(5,5)	8.3	8.3	3.5	3.5	5.1	9.3	9.3	4.1	4.1	4.4
		(10,10)	5.9	5.9	4.6	4.6	5.2	6.9	6.9	5	5	5.2
		(10,20)	5.6	5.6	4.5	4.5	4.9	6.4	6.4	5	5	5.1
		(20,20)	5.4	5.4	5	5	5.2	5.4	5.4	4.9	4.9	4.9
8	AR	(5,5)	10.1	10.1	4.2	4.2	6.8	10.4	10.4	5	5	5.6
		(10,10)	6.4	6.4	4.7	4.7	5.5	7	7	4.7	4.7	5.5
		(10,20)	6.6	6.6	5	5	6	7.3	7.3	5.3	5.3	6
		(20,20)	5.4	5.4	4.9	4.9	5.2	5.9	5.9	5.3	5.3	5.6
	CS	(5,5)	10	10	4.3	4.3	6.6	10.9	10.9	5.3	5.3	5.9
		(10,10)	6.2	6.2	4.5	4.5	5.3	6.9	6.9	5	5	5.5
		(10,20)	6.6	6.6	5.1	5.1	6	7.1	7.1	5	5	5.7
		(20,20)	5.2	5.2	4.6	4.6	4.9	5.9	5.9	5.2	5.2	5.4
	TP	(5,5)	9.1	9.1	4	4	6	9.7	9.7	4.3	4.3	5.3
		(10,10)	6.7	6.7	4.7	4.7	5.8	6.9	6.9	4.8	4.8	5.1
		(10,20)	6.9	6.9	5.4	5.4	6.2	7	7	5.1	5.1	5.7
		(20,20)	5.7	5.7	5.1	5.1	5.5	5.6	5.6	5.2	5.2	5.3

Table 10. Simulation results for type-I error level ($\alpha = 0.05$) for double exponential distribution, testing the hypothesis H_0^G, $d \in \{4, 8\}$ and different percentages r in case of the MCAR mechanism.

			$r = 10$					$r = 30$				
d	Cov	n	Classic		WBtstrp			Classic		WBtstrp		
			T_W	T_A	T_W^W	T_A^W	T_M^W	T_W	T_A	T_W^W	T_A^W	T_M^W
4	AR	(5,5)	8.8	8.8	3.6	3.6	5.7	9.8	9.8	4.4	4.4	5.2
		(10,10)	6	6	4.5	4.5	5.3	7	7	5.1	5.1	5.5
		(10,20)	6	6	4.8	4.8	5.5	6.6	6.6	4.8	4.8	5.2
		(20,20)	4.9	4.9	4.5	4.5	4.8	5.8	5.8	5.2	5.2	5.3
	CS	(5,5)	9	9	3.8	3.8	6.1	9.8	9.8	4.8	4.8	5.4
		(10,10)	6	6	4.4	4.4	5.6	7.4	7.4	5.4	5.4	5.8
		(10,20)	5.9	5.9	4.6	4.6	5.4	6.4	6.4	4.8	4.8	5.2
		(20,20)	5.3	5.3	4.7	4.7	5.1	5.7	5.7	5	5	5.3
	TP	(5,5)	8.5	8.5	3.6	3.6	5.6	9.8	9.8	4.3	4.3	4.8
		(10,10)	5.8	5.8	4.4	4.4	5	6.8	6.8	5	5	5.3
		(10,20)	5.8	5.8	4.6	4.6	5.3	6.4	6.4	4.9	4.9	5.3
		(20,20)	5.1	5.1	4.7	4.7	5	5.6	5.6	4.9	4.9	5
8	AR	(5,5)	10	10	4.2	4.2	6.9	10.7	10.7	4.9	4.9	6.1
		(10,10)	6.3	6.3	4.4	4.4	5.7	7.1	7.1	4.9	4.9	5.9
		(10,20)	6.3	6.3	4.9	4.9	6	7	7	5.1	5.1	5.8
		(20,20)	5.5	5.5	4.9	4.9	5.7	5.7	5.7	5.1	5.1	5.3
	CS	(5,5)	9.7	9.7	4	4	7.4	11.3	11.3	5.2	5.2	6.6
		(10,10)	6.6	6.6	4.7	4.7	6	7.4	7.4	5.2	5.2	5.9
		(10,20)	6.4	6.4	4.9	4.9	6.1	6.9	6.9	5.2	5.2	6
		(20,20)	5.1	5.1	4.5	4.5	5.1	5.6	5.6	5.1	5.1	5.4
	TP	(5,5)	9.4	9.4	3.8	3.8	6.4	10.2	10.2	4.6	4.6	5.7
		(10,10)	6.3	6.3	4.6	4.6	5.5	6.9	6.9	4.8	4.8	5.6
		(10,20)	6.5	6.5	5.2	5.2	6.2	7	7	5.1	5.1	5.8
		(20,20)	5.5	5.5	4.9	4.9	5.5	5.5	5.5	4.9	4.9	5.2

Table 11. Simulation results for type-I error level ($\alpha = 0.05$) for chi-square distribution, testing the hypothesis H_0^G, $d \in \{4, 8\}$ and different percentages r in case of the MCAR mechanism.

			$r = 10$					$r = 30$				
d	Cov	n	Classic		WBtstrp			Classic		WBtstrp		
			T_W	T_A	T_W^W	T_A^W	T_M^W	T_W	T_A	T_W^W	T_A^W	T_M^W
4	AR	(5,5)	8.7	8.7	3.8	3.8	5.7	9.9	9.9	4.3	4.3	5
		(10,10)	6	6	4.4	4.4	5.3	6.6	6.6	4.6	4.6	5.1
		(10,20)	6.1	6.1	4.8	4.8	5.5	6.5	6.5	4.9	4.9	5.4
		(20,20)	5.2	5.2	4.8	4.8	5.2	5.6	5.6	5	5	5.2
	CS	(5,5)	9.1	9.1	3.9	3.9	6.2	10	10	4.7	4.7	5.2
		(10,10)	6.3	6.3	4.6	4.6	5.6	6.8	6.8	5	5	5.4
		(10,20)	6.5	6.5	5.2	5.2	5.9	6.3	6.3	4.8	4.8	5.1
		(20,20)	5.5	5.5	5.1	5.1	5.4	5.6	5.6	5	5	5.1
	TP	(5,5)	9.1	9.1	3.7	3.7	5.7	9.5	9.5	4.2	4.2	4.8
		(10,10)	6	6	4.5	4.5	5.4	6.5	6.5	4.6	4.6	4.9
		(10,20)	6	6	4.8	4.8	5.4	6.5	6.5	4.9	4.9	5.4
		(20,20)	5.5	5.5	5	5	5.4	5.5	5.5	5	5	5.2
8	AR	(5,5)	10	10	4.1	4.1	6.8	10.7	10.7	5.1	5.1	6.3
		(10,10)	6.9	6.9	5.2	5.2	6.3	7.1	7.1	4.7	4.7	5.7
		(10,20)	6.6	6.6	5.3	5.3	6.2	7.2	7.2	5.1	5.1	5.9
		(20,20)	5.5	5.5	5	5	5.4	5.8	5.8	5.2	5.2	5.6
	CS	(5,5)	10.2	10.2	4.6	4.6	7.3	11.1	11.1	5.2	5.2	6.5
		(10,10)	6.7	6.7	4.8	4.8	6	7.2	7.2	4.9	4.9	5.8
		(10,20)	6.5	6.5	5.1	5.1	6.2	7.1	7.1	5.4	5.4	6
		(20,20)	5.2	5.2	4.7	4.7	5.2	6.1	6.1	5.4	5.4	5.7
	TP	(5,5)	9.2	9.2	4	4	6.4	10.1	10.1	4.6	4.6	5.9
		(10,10)	6.6	6.6	4.9	4.9	6.1	6.8	6.8	4.8	4.8	5.5
		(10,20)	6.4	6.4	5.1	5.1	5.9	6.7	6.7	5	5	5.6
		(20,20)	5.3	5.3	4.9	4.9	5.3	5.6	5.6	5.1	5.1	5.4

Table 12. Simulation results for type-I error level ($\alpha = 0.05$) for lognormal distribution, testing the hypothesis H_0^G, $d \in \{4, 8\}$ and different percentages r in case of the MCAR mechanism.

			$r = 10$					$r = 30$				
d	Cov	n	Classic		WBtstrp			Classic		WBtstrp		
			T_W	T_A	T_W^W	T_A^W	T_M^W	T_W	T_A	T_W^W	T_A^W	T_M^W
4	AR	(5,5)	8.7	8.7	3.6	3.6	6.5	9.6	9.6	4.4	4.4	5.6
		(10,10)	6.2	6.2	4.6	4.6	6	6.5	6.5	4.4	4.4	5.3
		(10,20)	6.6	6.6	5.5	5.5	6.4	7.1	7.1	5.2	5.2	5.9
		(20,20)	5.5	5.5	4.8	4.8	5.4	5.8	5.8	5.2	5.2	5.6
	CS	(5,5)	8.9	8.9	3.9	3.9	7	10.2	10.2	4.8	4.8	6.6
		(10,10)	6.4	6.4	4.6	4.6	6.1	6.6	6.6	4.7	4.7	5.7
		(10,20)	6.8	6.8	5.6	5.6	6.6	7	7	5.5	5.5	6
		(20,20)	5.8	5.8	5.2	5.2	5.8	5.8	5.8	5.2	5.2	5.6
	TP	(5,5)	9.2	9.2	3.6	3.6	6.6	9.6	9.6	4.2	4.2	5.5
		(10,10)	6.1	6.1	4.4	4.4	5.7	6.6	6.6	4.4	4.4	5.4
		(10,20)	6.7	6.7	5.3	5.3	6.3	6.7	6.7	5.2	5.2	5.7
		(20,20)	5.5	5.5	5	5	5.5	5.8	5.8	5.2	5.2	5.4
8	AR	(5,5)	9	9	4	4	7.7	11.2	11.2	5	5	7.8
		(10,10)	6.7	6.7	4.7	4.7	6.7	6.8	6.8	4.8	4.8	6.3
		(10,20)	7	7	5.6	5.6	6.8	6.8	6.8	5.2	5.2	6.4
		(20,20)	5.7	5.7	5.1	5.1	5.9	5.4	5.4	4.7	4.7	5.4
	CS	(5,5)	8.8	8.8	4	4	8	10.8	10.8	4.7	4.7	8
		(10,10)	6.3	6.3	4.7	4.7	6.4	7.2	7.2	4.8	4.8	6.9
		(10,20)	6.8	6.8	5.5	5.5	6.8	7	7	5.4	5.4	6.7
		(20,20)	5.6	5.6	5	5	5.9	5.8	5.8	4.8	4.8	5.7
	TP	(5,5)	9	9	3.9	3.9	7.4	10.5	10.5	4.9	4.9	6.8
		(10,10)	6.9	6.9	4.9	4.9	6.8	7.3	7.3	4.9	4.9	6.4
		(10,20)	6.7	6.7	5.4	5.4	6.4	6.6	6.6	5	5	5.7
		(20,20)	5.6	5.6	5	5	5.7	5.8	5.8	5	5	5.7

Table 13. Simulation results for type-I error level ($\alpha = 0.05$) for normal distribution, $d = 4$ and two different MAR mechanisms.

			$MAR1$					$MAR2$				
Hyp	Cov	n	Classic		WBtstrp			Classic		WBtstrp		
			T_W	T_A	T_W^W	T_A^W	T_M^W	T_W	T_A	T_W^W	T_A^W	T_M^W
H_0^T	AR	(5,5)	29.8	7.9	5.3	5.6	6.5	25.2	6.9	5	5	6
		(10,10)	13.7	6.1	5.3	5.3	5.6	11.4	5.4	4.5	4.8	5
		(10,20)	12.9	6	6	5.4	6.1	12.1	6	5.6	5.4	5.9
		(20,20)	7.8	5.1	4.8	4.9	5	8.4	5.6	5.3	5.4	5.5
	CS	(5,5)	29	7.5	5.9	5	6.5	26	6.7	5	4.8	6.2
		(10,10)	13.3	5.7	5.1	5	5.3	11.7	5.1	4.7	4.5	4.9
		(10,20)	12.8	5.7	6	5.3	5.9	12.2	5.5	5.8	5.1	5.8
		(20,20)	8.6	5.5	5.3	5.2	5.4	8.4	5.7	5.3	5.3	5.4
	TP	(5,5)	29.1	7.9	5.5	5.5	6.4	25.4	7.2	5	5.2	5.8
		(10,10)	12.6	5.9	4.7	5.2	5.6	10.9	5.8	4.4	5.2	5
		(10,20)	12.8	6.3	6	5.6	6	11.8	6	5.2	5.5	5.7
		(20,20)	7.8	5.2	4.9	4.9	5	8.1	5.4	5.2	5.2	5.3
H_0^{GT}	AR	(5,5)	30.2	7.7	6	5.4	6.8	26.7	7.1	5.2	5	6.2
		(10,10)	13.2	6.2	5.2	5.4	5.7	12.2	5.5	4.8	5	5.1
		(10,20)	13.1	6.2	6.2	5.6	6	12	5.4	5.7	4.9	5.4
		(20,20)	8.4	5.3	5.2	5.1	5.1	8.6	5.9	5.3	5.7	5.6
	CS	(5,5)	29.7	7.5	6.1	5	6.8	25.7	6.1	5.2	4.3	6
		(10,10)	13.3	5.8	5.2	5	5.3	12.3	5.2	4.8	4.7	5
		(10,20)	12.9	5.9	6.1	5.4	6	12	5.4	5.6	5	5.6
		(20,20)	8.2	5.5	5.1	5.3	5.3	8.2	5.4	5.2	5.2	5.3
	TP	(5,5)	29.3	8	5.8	5.5	6.3	27	7.7	5.5	5.5	6.2
		(10,10)	13.4	6.4	5.4	5.6	5.6	12.4	5.9	5	5.2	5.3
		(10,20)	12.8	6.2	6.4	5.7	6.2	11.8	5.7	5.4	5.2	5.5
		(20,20)	8.1	5.4	5	5	5.2	8	5.8	5.1	5.4	5.4

Table 14. Simulation results for type-I error level ($\alpha = 0.05$) for double exponential distribution, $d = 4$ and two different MAR mechanisms.

Hyp	Cov	n	$MAR1$					$MAR2$				
			Classic		WBtstrp			Classic		WBtstrp		
			T_W	T_A	T_W^W	T_A^W	T_M^W	T_W	T_A	T_W^W	T_A^W	T_M^W
H_0^T	AR	(5,5)	29.6	7.3	5.8	5	6.2	25.8	6.6	5.1	4.8	5.6
		(10,10)	12.6	5.9	4.9	5.1	5.3	11.6	5.3	4.6	4.8	4.9
		(10,20)	12.2	5.7	5.6	5.1	5.8	11.7	5.8	5.6	5.2	5.6
		(20,20)	9	5.9	5.5	5.7	5.7	8.4	5.6	5.5	5.4	5.4
	CS	(5,5)	29.6	7.2	5.9	5.2	6.2	25.6	6.8	4.8	4.8	5.8
		(10,10)	13.2	5.7	5.1	5	5.7	11.9	5	4.4	4.6	5
		(10,20)	12.9	5.7	5.8	5.2	6	11.8	5.4	5.6	5	5.8
		(20,20)	8.2	5.2	5.2	5	5.3	8.4	5.7	5.3	5.4	5.4
	TP	(5,5)	28.9	8	5.6	5.5	6.4	25.4	7	5.1	5.1	5.6
		(10,10)	12.9	6.2	4.8	5.5	5.4	11.4	5.7	4.4	5	5
		(10,20)	12.4	6.3	6.2	5.6	6	11.6	6	5.4	5.4	5.9
		(20,20)	8.1	5.5	4.8	5.3	5.2	8.4	5.6	5.4	5.3	5.2
H_0^{GT}	AR	(5,5)	30.2	7.7	6.2	5.1	6.6	27	6.9	5.4	4.9	6.1
		(10,10)	13.5	6.1	5.2	5.5	5.8	11.9	5.6	5	4.9	5.1
		(10,20)	13.3	6.2	6.3	5.8	6.3	11.6	5.4	5.4	4.8	5.3
		(20,20)	8.2	5.8	5.2	5.6	5.4	8.4	5.8	5.3	5.6	5.4
	CS	(5,5)	29.7	7.6	6	5.1	7	26.1	6.1	5.3	4.4	5.9
		(10,10)	13.7	5.7	5.6	5.1	5.5	12.3	5.2	5.1	4.9	5.2
		(10,20)	12.5	5.6	5.6	5.1	5.7	11.5	5.3	5.7	4.9	5.5
		(20,20)	8.3	5.3	5.2	5.1	5.3	8.2	5.4	5	5.1	5.1
	TP	(5,5)	30.2	8.3	6.1	5.8	7	27.2	7.7	5.7	5.2	6.2
		(10,10)	13	6.1	5.1	5.3	5.4	12.2	5.9	4.9	5.1	5.2
		(10,20)	13.6	6	6.4	5.4	6.1	11.7	5.6	5.3	5.1	5.3
		(20,20)	8.2	5.4	5.2	5.3	5.4	8.1	5.9	5	5.6	5.5

Table 15. Simulation results for type-I error level ($\alpha = 0.05$) for chi-square distribution, $d = 4$ and two different MAR mechanisms.

Hyp	Cov	n	$MAR1$					$MAR2$				
			Classic		WBtstrp			Classic		WBtstrp		
			T_W	T_A	T_W^W	T_A^W	T_M^W	T_W	T_A	T_W^W	T_A^W	T_M^W
H_0^T	AR	(5,5)	30.5	7.7	5.7	5.4	6.2	25	6.7	5.1	4.9	5.8
		(10,10)	12.9	6.1	5.4	5.3	5.9	11.5	5.5	4.5	5	5
		(10,20)	12.4	6.1	5.7	5.5	5.9	11.8	5.9	5.5	5.4	6
		(20,20)	8.2	5.3	4.8	5.2	5.3	8.1	5.6	5.2	5.4	5.4
	CS	(5,5)	28.2	7.1	5.6	4.9	6.5	25.9	6.6	4.9	4.7	6.1
		(10,10)	13.4	6	5	5.4	5.6	11.6	5	4.7	4.5	4.8
		(10,20)	12.7	5.4	5.7	5	5.7	11.9	5.7	5.7	5.1	5.7
		(20,20)	8.2	5.1	4.7	4.9	5	8.5	5.4	5.1	5.2	5.3
	TP	(5,5)	29.7	8.2	6.2	5.9	6.5	25.5	7.3	4.9	5.4	6
		(10,10)	12.8	5.8	5.1	5.1	5.3	11.3	5.7	4.3	5	4.8
		(10,20)	12.8	6.7	6.2	6.1	6.5	11.5	6.2	5.5	5.7	5.8
		(20,20)	7.8	5.3	4.8	5.2	5.3	7.9	5.4	5.1	5.3	5.4
H_0^{GT}	AR	(5,5)	29.9	7.4	5.7	4.9	6.3	26.3	6.9	5.3	5	6.2
		(10,10)	13	5.8	4.9	5.1	5.4	12.4	5.6	5	5.1	5.4
		(10,20)	13.1	6.3	6.3	5.5	6.3	11.7	5.6	5.8	4.9	5.6
		(20,20)	8.6	5.4	5.2	5.2	5.2	8.5	5.8	5.4	5.8	5.7
	CS	(5,5)	29.8	7.6	5.5	5.2	6.7	26.1	6.1	5.2	4.4	6.1
		(10,10)	13	5.6	5.1	5	5.2	12.7	5.4	5	4.8	5.1
		(10,20)	12.7	5.7	5.9	5.4	5.8	12	5.5	5.7	5.2	5.5
		(20,20)	8.8	5.3	5.3	5.1	5.4	8.1	5.3	5	5	5.1
	TP	(5,5)	30.2	8.3	5.8	5.7	6.6	26.8	7.7	5.1	5.3	6.4
		(10,10)	12.6	6.1	4.8	5.4	5.5	11.8	5.7	4.7	5.1	5.3
		(10,20)	13.2	6.3	6.3	5.6	6.2	11.9	5.6	5.4	4.9	5.6
		(20,20)	8.5	5.6	5.4	5.3	5.4	8.4	5.9	5.4	5.6	5.6

Table 16. Simulation results for type-I error level ($\alpha = 0.05$) for lognormal distribution, $d = 4$ and two different MAR mechanisms.

Hyp	Cov	n	$MAR1$					$MAR2$				
			Classic		WBtstrp			Classic		WBtstrp		
			T_W	T_A	T_W^W	T_A^W	T_M^W	T_W	T_A	T_W^W	T_A^W	T_M^W
H_0^T	AR	(5,5)	30.1	7.2	5.5	5	6	26.2	6.7	5.1	4.6	5.8
		(10,10)	13.4	5.6	5.4	4.9	5.6	11.9	5.3	4.9	4.7	4.9
		(10,20)	12.8	5.9	6.1	5.4	5.9	12.1	5.6	5.7	5.1	5.7
		(20,20)	8.6	5.5	5.3	5.2	5.3	8.1	5.3	5.5	5.3	5.5
	CS	(5,5)	30.1	7	5.8	4.7	5.9	26.4	5.9	5	4.4	5.2
		(10,10)	13.2	5.4	5.5	4.9	5.3	11.9	4.7	4.9	4.2	4.7
		(10,20)	12.8	5.5	6	5	5.8	12.1	5.3	5.8	4.9	5.5
		(20,20)	8.6	5.5	5.4	5.3	5.3	8.3	5.1	5.4	5	5.2
	TP	(5,5)	30.1	7.9	5.7	5.4	6.2	26.7	7.1	5.3	5.2	6
		(10,10)	13.7	6.1	5.8	5.4	5.7	11.9	5.5	4.7	5	5.2
		(10,20)	12.4	6.5	6.3	6	6.3	12.2	5.7	5.8	5.4	5.6
		(20,20)	8.5	5.5	5.5	5.2	5.2	8.7	5.5	5.7	5.3	5.4
H_0^{GT}	AR	(5,5)	28.4	7.1	5.2	4.9	6.1	26.2	6.6	4.8	4.6	5.9
		(10,10)	13.2	5.3	4.9	5	5.3	12.3	5.4	4.8	4.8	5.4
		(10,20)	12.4	6.1	5.9	5.6	6	11.8	5.6	5.6	5.1	5.7
		(20,20)	8.4	5.5	5.4	5.3	5.5	8.4	5.7	5.4	5.5	5.4
	CS	(5,5)	29.2	6.6	5.1	4.4	5.7	25.9	6.2	4.9	4.5	6.2
		(10,10)	13.2	5.6	5	5.1	5.4	12.3	5.1	4.9	4.8	5.1
		(10,20)	13.1	5.9	6.1	5.4	5.9	11.9	5.6	5.4	5.1	5.6
		(20,20)	8.1	5.1	5	4.9	5.2	8.3	5.4	5.2	5.3	5.3
	TP	(5,5)	29.4	7.7	5.5	5.3	6.6	26.6	7.4	5	5.2	6
		(10,10)	13	6.3	5.2	5.7	5.8	11.9	5.5	4.9	5	5.2
		(10,20)	12.8	6.7	5.8	6.1	6.2	11.6	5.5	5.4	5.1	5.5
		(20,20)	8.8	5.7	5.4	5.6	5.8	8	5.8	5	5.5	5.4

Table 17. Simulation results for type-I error level ($\alpha = 0.05$) for normal distribution, $d = 8$ and two different MAR mechanisms.

Hyp	Cov	n	$MAR1$					$MAR2$				
			Classic		WBtstrp			Classic		WBtstrp		
			T_W	T_A	T_W^W	T_A^W	T_M^W	T_W	T_A	T_W^W	T_A^W	T_M^W
H_0^T	AR	(5,5)	40.4	5.5	5.5	5.1	6.5	44.9	5.2	6.2	4.9	6.4
		(10,10)	36.4	5.3	4.7	5.6	5.6	34.3	5.1	5	5.4	5.7
		(10,20)	30.7	5.1	6.6	5.4	6.1	29.8	5	6.6	5.1	5.7
		(20,20)	15.4	5	5.2	5.3	5.4	15.8	5.1	4.9	5.3	5.3
	CS	(5,5)	41.1	4.2	5.5	4.2	6.3	48.4	3.5	6.7	3.8	5.6
		(10,10)	36	4.3	5	5.2	5.5	33.8	4.1	5.2	5	5.3
		(10,20)	31.7	4.7	6.2	5.4	5.9	30	4.2	6.4	5	5.9
		(20,20)	16	4.2	5.2	4.8	5	15.7	4.4	4.9	5.1	5.2
	TP	(5,5)	37.3	7	5.5	6	6.6	41.9	6.9	5.9	5.8	6.7
		(10,10)	36.7	5.9	4.8	5.6	5.6	34.2	6	5	5.7	5.7
		(10,20)	31.7	6.3	6.3	6.1	6.4	28.9	5.8	5.6	5.7	5.8
		(20,20)	15.7	5.5	5	5.5	5.3	15.8	5.3	5.1	5.2	5.2
H_0^{GT}	AR	(5,5)	39.6	5.3	5.5	4.8	6.1	44.3	4.9	6.3	4.7	6.3
		(10,10)	36.6	4.9	4.8	5.1	5.5	34.1	5.2	4.8	5.4	5.6
		(10,20)	32.1	5.6	6.6	5.9	6.4	30.6	5.3	6.6	5.6	6.2
		(20,20)	15.9	5	5.3	5.2	5.2	15.4	5	5.2	5.3	5.6
	CS	(5,5)	41.2	4	5.4	4	5.9	48.6	3.6	6.8	4.1	6
		(10,10)	36	4.3	5.1	5.2	5.8	34.4	4.1	4.9	5	5.5
		(10,20)	32	4.2	6.7	5	5.9	30.7	4.6	6.6	5.6	6.2
		(20,20)	16	4.5	5.1	5.2	5.2	15	4.6	5.1	5.2	5.5
	TP	(5,5)	37.8	7.2	5.3	5.8	6.6	40.5	6.6	5.7	5.6	6
		(10,10)	37.2	6	4.5	5.8	5.4	35.4	5.9	4.9	5.6	5.5
		(10,20)	32.1	6.1	6.2	5.8	6.4	31.4	6	6.5	5.7	6.2
		(20,20)	15.9	5.7	5.2	5.6	5.6	15.6	5.7	5	5.7	5.5

Table 18. Simulation results for type-I error level ($\alpha = 0.05$) for double exponential distribution, $d = 8$ and two different MAR mechanisms.

			$MAR1$					$MAR2$				
Hyp	Cov	n	Classic		WBtstrp			Classic		WBtstrp		
			T_W	T_A	T_W^W	T_A^W	T_M^W	T_W	T_A	T_W^W	T_A^W	T_M^W
H_0^T	AR	(5,5)	39	5.5	5.4	5.1	6.5	44.4	5.2	6.4	5	6.1
		(10,10)	36.4	5.5	5	5.7	5.6	33.9	5	5.2	5.3	5.6
		(10,20)	30.8	5.2	6.1	5.5	6.2	29.9	4.9	6.4	5.2	5.7
		(20,20)	16.3	5.1	5.2	5.3	5.2	15.7	5.2	5.3	5.4	5.4
	CS	(5,5)	41	4.1	5.7	4.2	5.6	48.7	3.4	6.8	3.9	5.4
		(10,10)	36	4	5	5	5.3	34.3	4.2	5.2	5.1	5.4
		(10,20)	30.8	4.2	6.2	5.1	5.8	30.3	4.2	6.4	5.1	5.6
		(20,20)	16.1	4.9	5.6	5.4	5.5	15.5	4.3	4.8	5	5.1
	TP	(5,5)	37.4	7.2	5.3	6.1	7	41.2	6.8	6	5.8	6.5
		(10,10)	36.2	5.7	4.3	5.4	5.4	34.1	6	4.6	5.6	5.5
		(10,20)	31.8	6	6.4	5.8	6.2	28.8	5.9	5.6	5.7	5.8
		(20,20)	15.3	5.8	4.9	5.6	5.5	15.9	5.6	5.2	5.3	5.4
H_0^{GT}	AR	(5,5)	39.6	5.6	5.8	5	6.5	44	5.1	6	4.6	6.6
		(10,10)	35.7	5.2	4.9	5.5	5.7	34.1	5.2	5	5.4	5.4
		(10,20)	31.5	5.1	5.9	5.5	6.1	30.2	5.1	6.6	5.4	5.9
		(20,20)	15.5	4.9	5.1	5	5.1	15.4	5.3	5.3	5.5	5.6
	CS	(5,5)	41	4.4	5.7	4.3	6.5	49.1	3.5	7	3.9	5.8
		(10,10)	35.3	4	5.2	4.8	5.4	34.4	3.9	5	4.9	5.2
		(10,20)	30.7	4.2	6.2	5	5.8	30.4	4.5	6.3	5.5	6.4
		(20,20)	16.2	4.8	5.2	5.3	5.3	14.8	4.1	5	4.8	5
	TP	(5,5)	37.9	6.8	4.9	5.6	6.6	40.7	6.7	5.6	5.5	6
		(10,10)	38.2	6.8	5.1	6.4	6.5	35.1	5.9	4.6	5.6	5.7
		(10,20)	31.1	6.2	6.3	5.9	6.3	30.6	5.9	6.7	5.8	6
		(20,20)	15.8	5.6	5.2	5.5	5.6	15.3	5.6	4.9	5.5	5.5

Table 19. Simulation results for type-I error level ($\alpha = 0.05$) for chi-square distribution, $d = 8$ and two different MAR mechanisms.

Hyp	Cov	n	$MAR1$					$MAR2$				
			Classic		WBtstrp			Classic		WBtstrp		
			T_W	T_A	T_W^W	T_A^W	T_M^W	T_W	T_A	T_W^W	T_A^W	T_M^W
H_0^T	AR	(5,5)	39.7	5.2	5.4	4.7	6.4	43.7	5.1	5.8	4.9	6.4
		(10,10)	36	5.1	5	5.5	5.8	34.7	5.2	4.9	5.5	5.5
		(10,20)	31.4	5.2	6.5	5.5	6.1	29.9	5	6.3	5.4	5.7
		(20,20)	15.7	4.9	5	5.1	5.2	15.4	5.1	5.2	5.4	5.4
	CS	(5,5)	41	4	5.2	4.1	5.8	48.5	3.4	6.4	3.8	5.8
		(10,10)	36.8	4.2	5.1	5.1	5.8	34.5	4.2	5.3	5.1	5.4
		(10,20)	31.4	4.2	6	5.2	5.6	29.6	4.2	6.2	5	5.9
		(20,20)	16.3	4.7	5.6	5.3	5.4	15.6	4.4	4.9	5.1	5.2
	TP	(5,5)	38	7	5.1	5.7	6.6	41.2	6.9	6	6	6.6
		(10,10)	37	5.9	4.4	5.6	5.6	34.7	6.2	4.4	5.9	5.9
		(10,20)	31.9	6	6.1	5.6	6	28.1	5.9	5.8	5.6	5.9
		(20,20)	15.4	5.7	5.1	5.8	5.7	15.5	5.4	5.1	5.4	5.4
H_0^{GT}	AR	(5,5)	39	5.2	5.2	4.8	6.5	43.8	5	6.1	4.6	6.1
		(10,10)	35.9	4.7	4.9	5.1	5.3	33.6	4.9	4.8	5.3	5.5
		(10,20)	30.9	5.4	6.7	5.8	6.3	30.2	5.3	6.3	5.7	6.1
		(20,20)	16.2	5.1	5.1	5.4	5.3	14.9	5.3	4.9	5.4	5.6
	CS	(5,5)	41.4	3.5	5.2	3.4	6	48.6	3.5	6.5	3.9	5.9
		(10,10)	35.8	3.9	5	5	5.3	34	4	4.7	4.9	5.2
		(10,20)	31	4.2	6.5	5.1	6	30.9	4.4	6.5	5.3	6
		(20,20)	15.6	4.5	5.3	5	5.2	15.1	4.4	5	5.1	5.3
	TP	(5,5)	37.3	7.2	5	6	6.9	39.8	6.4	5.5	5.5	6
		(10,10)	36.7	6.3	4.3	6	6	35.3	5.8	4.6	5.4	5.4
		(10,20)	32	5.9	6.2	5.8	6.1	30.2	5.9	6.1	5.8	6.1
		(20,20)	15.2	5.6	5	5.6	5.4	15.2	5.7	5	5.7	5.7

Table 20. Simulation results for type-I error level ($\alpha = 0.05$) for lognormal distribution, $d = 8$ and two different MAR mechanisms.

Hyp	Cov	n	$MAR1$					$MAR2$				
			Classic		WBtstrp			Classic		WBtstrp		
			T_W	T_A	T_W^W	T_A^W	T_M^W	T_W	T_A	T_W^W	T_A^W	T_M^W
H_0^T	AR	(5,5)	38.2	5	5.1	4.6	5.7	42.3	4.6	5.7	4.6	5.8
		(10,10)	36.1	4.4	4.6	5.1	5.6	35	5	4.9	5.6	5.9
		(10,20)	30.6	4.8	5.7	5.4	5.8	29.4	4.8	5.9	5.4	5.7
		(20,20)	15.8	4.8	5.6	5.1	5.4	15.3	5	5.4	5.4	5.5
	CS	(5,5)	39.3	3.2	5.3	3.6	5.3	46.1	3.2	6.1	3.5	5.2
		(10,10)	36	4	5	5	5.3	34.8	4	5	5.1	5.2
		(10,20)	30.4	4	6.4	5.1	5.7	29.4	4	6	5	5.7
		(20,20)	15.6	4.4	5.2	5.1	5.1	15.8	4.1	5.2	4.8	4.9
	TP	(5,5)	36.8	6.5	4.5	5.5	6.2	39.1	6.6	5.5	5.8	6.1
		(10,10)	39.2	6.4	5.3	6.3	6.3	35.6	6.1	4.9	5.9	5.9
		(10,20)	31.1	5.5	6.2	5.4	6	28.5	5.5	6.1	5.5	5.7
		(20,20)	17.3	5.6	6.1	5.7	5.6	15.9	5.3	6.1	5.4	5.3
H_0^{GT}	AR	(5,5)	37.7	5	4.8	4.7	5.8	41.2	4.6	5.8	4.4	5.9
		(10,10)	36.3	4.5	4.3	5	5.5	34.1	4.7	4.2	5.2	5.4
		(10,20)	30.3	4.2	5.8	4.7	5.3	29	4.8	5.8	5.4	5.9
		(20,20)	14.9	5.1	5.1	5.6	5.2	14.9	5.2	4.8	5.6	5.8
	CS	(5,5)	39.5	3.8	5.1	4	5.6	45.7	3.1	6	3.6	5.4
		(10,10)	35.5	3.7	4.4	4.7	5.3	33.7	3.6	4.4	4.7	4.8
		(10,20)	30	4.2	6	5.2	5.9	29	4	5.8	5.1	5.8
		(20,20)	15.7	4.7	5	5.4	5.5	14.7	4.1	5.2	5	5
	TP	(5,5)	36.7	6.4	4.7	5.5	6.2	38.4	6.3	5	5.4	6
		(10,10)	37.5	5.5	4	5.6	5.7	34	5.6	3.8	5.4	5.5
		(10,20)	30.3	5.6	5.6	5.5	6.1	29.2	5.8	5.9	5.9	6.2
		(20,20)	15.4	5.4	4.9	5.2	5.3	14.9	5.8	4.8	5.8	5.8

Table 21. Simulation results for type-I error level ($\alpha = 0.05$) for normal distribution, testing the hypothesis H_0^G, $d \in \{4, 8\}$ and two different MAR mechanisms.

			$MAR1$					$MAR2$				
d	Cov	n	Classic		WBtstrp			Classic		WBtstrp		
			T_W	T_A	T_W^W	T_A^W	T_M^W	T_W	T_A	T_W^W	T_A^W	T_M^W
4	AR	(5,5)	8.8	8.8	3.8	3.8	5.1	8.4	8.4	3.3	3.3	5
		(10,10)	6	6	4.2	4.2	4.9	5.8	5.8	4.5	4.5	4.9
		(10,20)	5.5	5.5	4.3	4.3	4.7	5.7	5.7	4.7	4.7	4.9
		(20,20)	5.5	5.5	5.2	5.2	5.2	5.1	5.1	4.9	4.9	4.9
	CS	(5,5)	9	9	4	4	5.5	8	8	3.1	3.1	5.1
		(10,10)	6.2	6.2	4.7	4.7	5.4	6	6	4.4	4.4	5.1
		(10,20)	5.5	5.5	4.3	4.3	4.5	5.5	5.5	4.4	4.4	4.8
		(20,20)	5.3	5.3	4.9	4.9	4.9	5.1	5.1	4.8	4.8	4.9
	TP	(5,5)	8.9	8.9	3.9	3.9	5.2	8.2	8.2	3.3	3.3	5.1
		(10,10)	6.6	6.6	4.8	4.8	5.4	6	6	4.5	4.5	5.1
		(10,20)	5.9	5.9	4.7	4.7	5.1	5.5	5.5	4.4	4.4	4.9
		(20,20)	5	5	4.7	4.7	4.7	5	5	4.9	4.9	4.9
8	AR	(5,5)	9.6	9.6	4.2	4.2	5.7	9	9	3.6	3.6	5.2
		(10,10)	6.6	6.6	4.6	4.6	5.3	6.3	6.3	4.7	4.7	5.4
		(10,20)	6.1	6.1	4.7	4.7	5.4	6.3	6.3	5	5	5.7
		(20,20)	6.2	6.2	5.8	5.8	6	5.5	5.5	5	5	5.3
	CS	(5,5)	9.7	9.7	4.5	4.5	6	9.3	9.3	4	4	6
		(10,10)	6.6	6.6	4.8	4.8	5.5	6.5	6.5	4.8	4.8	5.7
		(10,20)	6.2	6.2	4.7	4.7	5.3	6.2	6.2	4.8	4.8	5.6
		(20,20)	5.8	5.8	5.2	5.2	5.5	5.4	5.4	4.9	4.9	5.2
	TP	(5,5)	9.5	9.5	4.2	4.2	5.6	8.8	8.8	3.4	3.4	5.2
		(10,10)	6.7	6.7	4.7	4.7	5.7	6.5	6.5	4.8	4.8	5.6
		(10,20)	5.8	5.8	4.4	4.4	5	6	6	4.8	4.8	5.4
		(20,20)	5.8	5.8	5.2	5.2	5.4	5.4	5.4	5	5	5.3

Table 22. Simulation results for type-I error level ($\alpha = 0.05$) for double exponential distribution,testing the hypothesis H_0^G, $d \in \{4, 8\}$ and two different MAR mechanisms.

d	Cov	n	$MAR1$					$MAR2$				
			Classic		WBtstrp			Classic		WBtstrp		
			T_W	T_A	T_W^W	T_A^W	T_M^W	T_W	T_A	T_W^W	T_A^W	T_M^W
4	AR	(5,5)	9	9	4.2	4.2	5.9	8.1	8.1	3.3	3.3	5.2
		(10,10)	6.5	6.5	4.6	4.6	5.4	5.9	5.9	4.4	4.4	5.2
		(10,20)	5.9	5.9	4.7	4.7	5.2	5.4	5.4	4.3	4.3	4.8
		(20,20)	5.4	5.4	4.9	4.9	5.2	5.4	5.4	5	5	5.2
	CS	(5,5)	9.6	9.6	4.7	4.7	6.2	8.2	8.2	3.5	3.5	5.7
		(10,10)	6.4	6.4	4.7	4.7	5.6	5.9	5.9	4.6	4.6	5.4
		(10,20)	6.2	6.2	4.9	4.9	5.6	5.6	5.6	4.4	4.4	5
		(20,20)	5.3	5.3	4.8	4.8	5.2	5.2	5.2	4.9	4.9	5.2
	TP	(5,5)	8.6	8.6	3.8	3.8	5.3	8.2	8.2	3.4	3.4	5.1
		(10,10)	5.6	5.6	4.2	4.2	4.8	6.1	6.1	4.8	4.8	5.5
		(10,20)	5.9	5.9	4.8	4.8	5.3	5.5	5.5	4.4	4.4	4.8
		(20,20)	4.9	4.9	4.6	4.6	4.7	5.6	5.6	5.2	5.2	5.4
8	AR	(5,5)	10.2	10.2	4.9	4.9	6.9	9.2	9.2	4.1	4.1	6.4
		(10,10)	6.5	6.5	4.7	4.7	5.9	6.2	6.2	4.7	4.7	5.7
		(10,20)	6.4	6.4	4.7	4.7	5.7	6.3	6.3	5	5	6
		(20,20)	6.1	6.1	5.3	5.3	5.8	5.5	5.5	5	5	5.5
	CS	(5,5)	10.5	10.5	4.9	4.9	7.2	9.7	9.7	4.4	4.4	7.2
		(10,10)	6.6	6.6	4.6	4.6	6.1	6.7	6.7	4.9	4.9	6.1
		(10,20)	6	6	4.7	4.7	5.7	6.2	6.2	5	5	5.8
		(20,20)	5.5	5.5	4.8	4.8	5.4	5.4	5.4	4.8	4.8	5.4
	TP	(5,5)	10.4	10.4	4.5	4.5	6.2	8.5	8.5	3.3	3.3	5.7
		(10,10)	6.6	6.6	4.5	4.5	5.3	6.4	6.4	4.7	4.7	5.7
		(10,20)	6.1	6.1	4.8	4.8	5.6	6.2	6.2	4.9	4.9	5.6
		(20,20)	5.7	5.7	5.2	5.2	5.5	5.4	5.4	4.9	4.9	5.3

Table 23. Simulation results for type-I error level ($\alpha = 0.05$) for chi-square distribution,testing the hypothesis H_0^G, $d \in \{4, 8\}$ and two different MAR mechanisms.

d	Cov	n	$MAR1$					$MAR2$				
			Classic		WBtstrp			Classic		WBtstrp		
			T_W	T_A	T_W^W	T_A^W	T_M^W	T_W	T_A	T_W^W	T_A^W	T_M^W
4	AR	(5,5)	9.4	9.4	4.3	4.3	5.8	8.3	8.3	3.5	3.5	5.5
		(10,10)	6	6	4.4	4.4	5	6.3	6.3	4.8	4.8	5.5
		(10,20)	6	6	4.9	4.9	5.3	6.3	6.3	5.3	5.3	5.8
		(20,20)	5.2	5.2	4.7	4.7	4.8	5.5	5.5	5.2	5.2	5.3
	CS	(5,5)	9.1	9.1	4.4	4.4	6	8.9	8.9	3.6	3.6	5.9
		(10,10)	6.3	6.3	4.7	4.7	5.4	6.2	6.2	4.7	4.7	5.4
		(10,20)	6.5	6.5	5.2	5.2	5.8	5.5	5.5	4.3	4.3	4.9
		(20,20)	5.4	5.4	4.8	4.8	5.1	5.7	5.7	5.3	5.3	5.7
	TP	(5,5)	8.9	8.9	3.8	3.8	5.4	8.4	8.4	3.8	3.8	5.6
		(10,10)	6.1	6.1	4.3	4.3	5	6	6	4.6	4.6	5.3
		(10,20)	5.9	5.9	4.7	4.7	5.1	6	6	4.9	4.9	5.3
		(20,20)	5.5	5.5	5	5	5.2	5.3	5.3	5	5	5.2
8	AR	(5,5)	10.1	10.1	5	5	6.6	9.5	9.5	4	4	6.2
		(10,10)	6.9	6.9	5	5	6.1	6.5	6.5	4.7	4.7	5.8
		(10,20)	6.7	6.7	5.2	5.2	6	6.3	6.3	5	5	5.9
		(20,20)	6	6	5.4	5.4	5.8	5.4	5.4	4.9	4.9	5.3
	CS	(5,5)	10.3	10.3	5.1	5.1	6.9	9.9	9.9	4.1	4.1	7
		(10,10)	6.8	6.8	5.1	5.1	6	6.7	6.7	4.8	4.8	6.1
		(10,20)	6.5	6.5	5.1	5.1	6	6.3	6.3	5.1	5.1	5.9
		(20,20)	5.7	5.7	5	5	5.5	5.8	5.8	5.3	5.3	5.7
	TP	(5,5)	10.2	10.2	4.5	4.5	6	8.9	8.9	3.6	3.6	5.9
		(10,10)	6.9	6.9	5	5	5.8	6.4	6.4	4.5	4.5	5.7
		(10,20)	6.2	6.2	4.7	4.7	5.4	6.6	6.6	5.2	5.2	6
		(20,20)	5.4	5.4	4.8	4.8	5	5.6	5.6	5	5	5.5

Table 24. Simulation results for type-I error level ($\alpha = 0.05$) for lognormal distribution,testing the hypothesis H_0^G, $d \in \{4, 8\}$ and two different MAR mechanisms.

d	Cov	n	$MAR1$					$MAR2$				
			Classic		WBtstrp			Classic		WBtstrp		
			T_W	T_A	T_W^W	T_A^W	T_M^W	T_W	T_A	T_W^W	T_A^W	T_M^W
4	AR	(5,5)	9.7	9.7	4.4	4.4	6.7	8.9	8.9	4	4	6.6
		(10,10)	6.2	6.2	4.4	4.4	5.7	6.7	6.7	4.8	4.8	6.2
		(10,20)	6.8	6.8	5.4	5.4	6.2	6.5	6.5	5.2	5.2	6.2
		(20,20)	5.7	5.7	5	5	5.7	5.7	5.7	5	5	5.7
	CS	(5,5)	9.6	9.6	4.3	4.3	7	9.4	9.4	4.1	4.1	7.4
		(10,10)	6.7	6.7	4.7	4.7	6.4	6.7	6.7	4.8	4.8	6.4
		(10,20)	6.8	6.8	5.4	5.4	6.4	6.7	6.7	5.2	5.2	6.3
		(20,20)	5.8	5.8	5.1	5.1	5.7	5.7	5.7	4.8	4.8	5.4
	TP	(5,5)	9.1	9.1	4.1	4.1	6.1	9.4	9.4	3.8	3.8	6.6
		(10,10)	6.1	6.1	4.2	4.2	5.5	6.2	6.2	4.4	4.4	5.8
		(10,20)	6	6	4.7	4.7	5.5	6.5	6.5	5.3	5.3	6.1
		(20,20)	5.3	5.3	4.7	4.7	5.3	5.5	5.5	4.9	4.9	5.6
8	AR	(5,5)	10.2	10.2	4.6	4.6	8.1	9.5	9.5	4.2	4.2	8.1
		(10,10)	7	7	5	5	6.8	6.3	6.3	4.7	4.7	6.4
		(10,20)	6.8	6.8	5.5	5.5	6.8	6.7	6.7	5.4	5.4	6.6
		(20,20)	5.7	5.7	4.9	4.9	5.8	5.8	5.8	5.1	5.1	5.8
	CS	(5,5)	10.3	10.3	4.8	4.8	8.7	9.2	9.2	4.3	4.3	8.2
		(10,10)	6.9	6.9	4.8	4.8	6.9	6.4	6.4	4.5	4.5	6.5
		(10,20)	7	7	5.8	5.8	6.9	6.9	6.9	5.3	5.3	6.8
		(20,20)	5.8	5.8	5	5	6	5.7	5.7	4.8	4.8	5.8
	TP	(5,5)	10.4	10.4	4.8	4.8	7.4	9.3	9.3	4.2	4.2	7.2
		(10,10)	6.8	6.8	4.8	4.8	6.1	6.6	6.6	4.8	4.8	6.4
		(10,20)	6.7	6.7	5.3	5.3	6.3	6.8	6.8	5.6	5.6	6.6
		(20,20)	5.4	5.4	4.8	4.8	5.5	5.6	5.6	5	5	5.7

Table 25. Simulation results for type-I error level ($\alpha = 0.05$) of the tests for different distributions under varying covariance structures with sample sizes ($n = 299, d = 3$) and missingness inspired from the fluvoxamine study data.

Dist	Cov	Classic		WBtstrp		
		T_W	T_A	T_W^W	T_A^W	T_M^W
Ordinal	–	5.2	4.9	5.2	5.2	5.1
Normal	AR	5	4.8	4.9	4.9	4.9
Normal	CS	5.3	5.2	5.1	5.3	5.2
Normal	TP	4.7	4.6	4.6	4.8	4.7
Double exponential	AR	5	4.8	4.8	4.8	4.9
Double exponential	CS	5.2	5.3	5	5.2	5
Double exponential	TP	4.9	4.6	4.7	4.7	4.7
Chi-square	AR	5.1	4.9	4.9	5	4.9
Chi-square	CS	5.1	5.1	5	5.1	5.2
Chi-square	TP	4.9	4.8	4.8	4.9	5
Lognormal	AR	6.5	5.8	6.3	5.7	5.9
Lognormal	CS	5.3	5	5.2	5	5.1
Lognormal	TP	8.9	7	8.6	6.9	7.4

Table 26. Simulation results for type-I error level ($\alpha = 0.05$) of the tests for different distributions under varying covariance structures with sample sizes ($n_1 = 88, n_2 = 84, d = 3$) and MCAR data with missing rate ($r = 30\%$) inspired from the skin disorder study data.

Hyp	Dist	Cov	Classic		WBtstrp		
			T_W	T_A	T_W^W	T_A^W	T_M^W
H_0^G	Ordinal	-	5.7	5.7	5.5	5.5	5.6
	Normal	AR	4.9	4.9	5	5	4.9
	Normal	CS	5	5	5.1	5.1	5
	Normal	TP	4.9	4.9	5	5	4.9
	Double exponential	AR	5.3	5.3	5.3	5.3	5.3
	Double exponential	CS	5.1	5.1	5.2	5.2	5.1
	Double exponential	TP	5.3	5.3	5.3	5.3	5.3
	Chi-square	AR	4.9	4.9	4.8	4.8	4.8
	Chi-square	CS	4.8	4.8	4.9	4.9	4.9
	Chi-square	TP	4.8	4.8	4.9	4.9	4.9
	Lognormal	AR	5	5	5	5	5
	Lognormal	CS	4.8	4.8	4.8	4.8	4.8
	Lognormal	TP	5	5	5.1	5.1	5.1
H_0^T	Ordinal	TP	5.5	5.1	5.1	5.1	5.2
	Normal	AR	5.5	5.3	5.1	5.3	5.2
	Normal	CS	5.7	5.2	5.2	5.2	5.2
	Normal	TP	5.4	5.3	5	5.2	5.1
	Double exponential	AR	5.8	5.5	5.5	5.5	5.6
	Double exponential	CS	5.8	5.4	5.4	5.2	5.4
	Double exponential	TP	5.7	5.4	5.3	5.4	5.5
	Chi-square	AR	5.7	5.4	5.4	5.3	5.4
	Chi-square	CS	5.7	5.2	5.3	5	5.2
	Chi-square	TP	5.7	5.4	5.4	5.4	5.2
	Lognormal	AR	6.2	5.9	6	5.8	5.9
	Lognormal	CS	5.8	5.2	5.4	5.1	5.3
	Lognormal	TP	7.4	6.3	7	6.2	6.5
H_0^{GT}	Ordinal	TP	5.6	5.3	5.3	5.4	5.4
	Normal	AR	5.2	4.7	4.8	4.7	4.8
	Normal	CS	5.3	4.9	5	4.9	5.1
	Normal	TP	5.3	4.7	4.9	4.8	4.8
	Double exponential	AR	5.3	4.7	4.9	4.7	4.8
	Double exponential	CS	5.2	5	4.9	5	5
	Double exponential	TP	5.2	4.8	4.8	4.8	4.8
	Chi-square	AR	5.3	4.9	4.9	4.9	4.9
	Chi-square	CS	5.3	5	4.9	5	5
	Chi-square	TP	5.3	4.8	5	4.9	5
	Lognormal	AR	5.3	5	4.9	5	4.9
	Lognormal	CS	5.2	5	4.9	5	5
	Lognormal	TP	5.3	4.9	5	4.9	5

Table 27. Simulation results for type-I error level ($\alpha = 0.05$) for normal distribution and different percentages r in case of the MCAR mechanism.

			$r = 10$						$r = 30$					
d	Cov	n	WBtstrp			Alternatives			WBtstrp			Alternatives		
			T_W^*	T_A^*	T_M^*	$A1$	$A2$	M	T_W^*	T_A^*	T_M^*	$A1$	$A2$	M
4	AR	10	5	5.2	5.7	7.5	5	10.3	6.2	5.9	7.4	9.6	7.1	15
		15	5.4	5.3	5.8	6.8	5.3	8.7	5.8	5.7	6.3	8.1	6.4	11.1
		20	4.9	4.9	5.2	5.9	4.9	7.1	4.6	5	5.3	6.7	5.5	8.9
		30	5.6	5.6	5.7	6.2	5.6	6.7	5.5	5.6	5.8	6.7	6	7.8
	CS	10	5	5	5.9	7.3	4.7	10.6	6.3	5.8	6.9	9.3	6.3	14.7
		15	5.1	5	5.3	6	4.5	8.1	5.3	5	5.7	7.2	5.5	10.4
		20	5.4	5.5	5.6	6.3	5.2	7.2	5.4	5.8	6	7.3	6	9.5
		30	5.4	5.3	5.6	5.7	5.1	6.7	5.4	5.3	5.8	6.3	5.5	8
	TP	10	5.1	6.1	6.7	8.9	6.5	11	5.9	6	7.2	9.8	7.2	14.3
		15	5.3	5.6	5.8	7.5	6.2	8.5	5.3	5.4	5.8	8.1	6.6	10.6
		20	4.9	5.8	5.8	7.1	6.2	7.5	5.8	6	6.4	7.9	6.9	9.4
		30	4.8	5.2	5.4	6.3	5.5	6.3	5.4	5.4	5.6	6.4	5.8	7.6
8	AR	10	5.6	5.6	5.9	5.9	4.2	13.2	6.5	5.7	7.6	7.2	5.1	21.7
		15	5.2	5.6	5.8	5.7	4.5	10.1	4.5	5.2	5.8	6	4.8	13.9
		20	5.2	5.7	5.7	5.8	5	8.6	5.6	5.8	6	6.2	5.3	11.8
		30	4.8	5.1	5.1	5.2	4.5	6.6	5.4	5.3	5.4	5.5	5	8.8
	CS	10	5.2	5	6.1	4.2	2.7	13.6	6.4	5	7.1	5.6	3.7	20.4
		15	4.9	5	5.7	4.2	3	9.9	4.3	4.8	5.8	5	3.9	13.6
		20	5.2	5.2	5.5	4.5	3.6	8.7	5.4	5	5.4	4.8	4.1	11
		30	5.5	5.2	5.3	4.7	4.1	6.9	5.2	5.1	5.2	4.9	4.3	8.5
	TP	10	6.3	6.2	6.4	8.2	6.2	13.4	6.5	6.3	7.7	9.2	7	21.6
		15	4.8	5.7	5.8	7.2	5.9	9.7	4.7	5.5	5.9	7.1	6	13.6
		20	4.8	5.3	5.2	6.5	5.4	8.3	4.7	5.4	5.4	6.7	5.7	11.2
		30	5	5.2	5.1	6.1	5.4	7	4.9	5.1	5.2	5.9	5.3	8.7

Table 28. Simulation results for type-I error level ($\alpha = 0.05$) for double exponential distribution and different percentages r in case of the MCAR mechanism.

			$r = 10$						$r = 30$					
d	Cov	n	WBtstrp			Alternatives			WBtstrp			Alternatives		
			T_W^*	T_A^*	T_M^*	$A1$	$A2$	M	T_W^*	T_A^*	T_M^*	$A1$	$A2$	M
4	AR	10	5.1	5.3	6.2	7.8	5.2	10.8	5.7	6	7.4	9.9	7	15
		15	5	5	5.4	6.8	5.2	8.5	5.1	5.1	5.7	7.4	5.7	10.5
		20	4.8	5.2	5.2	6.3	5.1	7.5	5.2	5.2	5.5	6.9	5.8	8.7
		30	5.2	5.3	5.3	5.9	5.2	6.6	4.7	5.1	5	6.2	5.4	7.3
	CS	10	5.1	5.2	5.8	7.3	4.7	10.2	5.6	5.4	6.9	8.9	6.2	14.8
		15	5.5	5.2	5.8	6.4	5	8.5	5.4	5.2	5.8	7.3	5.6	10.9
		20	5	4.8	5	5.6	4.6	7.4	4.9	4.8	5	6.3	5.1	9.1
		30	5.3	5.1	5.2	5.6	4.9	6.3	5.3	5.3	5.6	6.3	5.5	8.1
	TP	10	4.8	5.6	6.1	8	5.9	10.6	6	6	6.9	10.1	7.5	14.4
		15	5.1	5.6	5.8	7.5	6	8.7	5.3	5.6	6.2	8.2	6.7	11.2
		20	4.9	5.4	5.3	6.7	5.7	7.5	5	5.1	5.5	7.1	6	8.7
		30	5	5	5.2	6	5.2	6.3	4.9	5	5.2	6.1	5.6	7.5
8	AR	10	5.3	5.4	5.9	5.7	3.7	13.3	6.2	5.9	7.3	7.4	5	21.1
		15	4.8	5	5.5	5.3	4.2	10.1	4.5	5.4	5.7	5.9	4.6	13.7
		20	5	5.1	5.5	5.3	4.5	8.6	5.3	5.3	5.7	5.9	4.9	11.3
		30	4.6	5.1	5	5.2	4.7	7.1	5.2	5.2	5.1	5.5	4.8	8.4
	CS	10	5.6	4.9	5.8	4.2	2.6	12.8	6.3	5	6.9	5.8	3.6	20.2
		15	4.9	4.7	5.1	3.8	2.8	9.4	4.7	5.3	6.4	5.5	4.3	14.1
		20	5	5.2	5.5	4.5	3.7	8.6	5.6	5.4	5.6	5.2	4.3	11.4
		30	5.3	5.6	5.6	5	4.5	7.2	5.1	5.1	5.1	4.9	4.4	8.3
	TP	10	5.7	6.1	6.4	7.8	6	13.9	6	6.3	7.9	8.9	6.7	22.1
		15	5.3	6.1	6	7.4	6.1	10.9	4.3	5.6	5.8	7.1	6	14.2
		20	5	5.7	5.7	6.8	5.9	8.7	5.3	5.3	5.6	6.6	5.8	11.7
		30	5.1	5.2	5.2	6	5.3	7.2	5.1	5.7	5.6	6.4	6	8.6

Table 29. Simulation results for type-I error level ($\alpha = 0.05$) for chi-Square distribution and different percentages r in case of the MCAR mechanism.

			$r = 10$						$r = 30$					
d	Cov	n	WBtstrp			Alternatives			WBtstrp			Alternatives		
			T_W^*	T_A^*	T_M^*	$A1$	$A2$	M	T_W^*	T_A^*	T_M^*	$A1$	$A2$	M
4	AR	10	5	5.2	5.6	7.5	5.2	11.2	5.6	5.6	6.9	9.4	6.8	14.7
		15	5.1	5.4	5.5	6.9	5.4	8.7	5.4	5.1	5.9	7.6	6.1	11.4
		20	4.9	5.2	5.4	6.1	5.3	7.7	5.3	5.4	6	7.2	6.1	9.5
		30	5.2	5.4	5.3	5.9	5.3	6.5	5.6	5.5	5.7	6.7	6	8
	CS	10	4.8	5	5.8	7	4.6	11.2	5.9	5.4	6.6	9	6.3	15
		15	5.4	5.1	5.6	6.1	4.7	8.9	5.7	5.3	6	7.3	5.7	11.2
		20	5.5	5.3	5.8	6.2	5.2	8.3	5.7	5.6	6	7.2	6.1	9.7
		30	5.1	5.1	5.2	5.5	4.9	6.6	5	5	5.2	6.2	5.4	7.8
	TP	10	5.1	5.8	6.3	9	6.3	11.4	5.7	5.9	6.9	9.9	7.3	14.9
		15	4.8	5.5	5.5	7.2	5.9	8.2	5.5	5.4	6	8.3	6.7	11
		20	5	5.2	5.3	6.6	5.6	7.3	5	5.4	5.7	7.5	6.4	9.5
		30	5.2	5.1	5.1	6	5.3	6.4	5.2	5.2	5.4	6.4	5.8	7.5
8	AR	10	5.7	5.6	6.2	5.8	3.9	14.6	5.8	5.7	7.1	7.2	5.1	21.6
		15	5.4	5.2	5.7	5.6	4.4	10.9	4.4	5.4	6	6.1	5	13.9
		20	5	5.5	5.6	5.6	4.6	9	5.1	5.7	5.7	6	5.1	11.5
		30	5	5.1	5.2	5.1	4.5	7.8	5.3	5.4	5.5	5.8	5.2	9.1
	CS	10	5.4	4.9	5.6	4	2.4	13.7	6.1	5.2	6.9	6	3.9	20.8
		15	5.2	4.9	5.3	4	3	10	4.5	5.3	6	5.5	4.2	14.3
		20	5.1	5.3	5.4	4.4	3.4	8.6	5.2	5.2	5.5	5	4.2	11.4
		30	4.9	5	5.3	4.4	3.8	7.2	4.9	5	5	4.8	4.2	8.1
	TP	10	5.7	6.5	6.9	8.7	6.4	14.7	6.3	6.2	7.8	8.9	6.6	21.7
		15	4.6	5.8	5.7	6.9	5.9	10	4.4	6.1	6.3	7.7	6.4	15
		20	5.5	5.7	5.6	6.8	6	8.8	5.4	5.4	5.5	6.6	5.6	11.1
		30	5.2	5.1	5.1	5.9	5.3	7.1	4.9	5.2	5.3	5.9	5.5	8.8

Table 30. Simulation results for type-I error level ($\alpha = 0.05$) for lognormal distribution and different percentages r in case of the MCAR mechanism.

			$r = 10$						$r = 30$					
d	Cov	n	WBtstrp			Alternatives			WBtstrp			Alternatives		
			T_W^*	T_A^*	T_M^*	$A1$	$A2$	M	T_W^*	T_A^*	T_M^*	$A1$	$A2$	M
4	AR	10	5.5	5	5.6	7.5	5.2	13.3	5.2	5.3	6.1	9.2	6.5	15.5
		15	5.2	5.1	5.4	6.6	4.9	10	5.1	5	5.4	7.4	5.9	11.4
		20	5.9	5.4	5.7	6.6	5.3	8.7	5.1	4.9	5.2	6.5	5.4	9.7
		30	4.7	4.7	5.1	5.4	4.8	6.8	5.1	5.1	5.2	6.1	5.5	8.2
	CS	10	5.4	4.6	5.4	6.8	4.6	12.9	5.6	5.3	6.4	9.2	6.3	15.4
		15	6.1	4.8	5.5	6	4.5	10.6	5.7	5	5.6	7.5	5.6	12
		20	5.9	5	5.3	6	4.9	9	5.8	5.2	5.7	6.8	5.6	10.7
		30	5.3	4.9	5.4	5.5	4.9	8.1	5.6	5.2	5.5	5.9	5.3	8
	TP	10	5.6	5.8	6.1	8.2	6	12.7	5.8	5.8	6.7	9.9	7.5	16.2
		15	5.5	4.8	5	6.3	4.9	9.4	5.7	5.7	6	8.2	6.4	12
		20	5.8	5.2	5.5	6.5	5.5	8.6	5.4	5.1	5.4	7.1	5.8	10.1
		30	5.6	5	5.2	5.8	5.3	7.3	5.4	5.1	5.4	6.4	5.6	8.3
8	AR	10	5.4	5	5.5	5.2	3.6	17.8	5.3	5.8	6.7	7.2	4.9	23.6
		15	5.3	5.2	5.5	5	4	13.1	4.3	5.5	5.8	5.9	4.7	16.6
		20	5.7	5.3	5.6	5.2	4.4	11.3	5	5.4	5.6	5.6	4.9	12.9
		30	5.1	5.2	5.1	4.9	4.4	8.3	4.9	5.2	5.3	5	4.5	9.7
	CS	10	5.5	4.7	5.4	3.8	2.4	16.2	6	5.2	6.5	5.5	3.7	22.1
		15	4.7	4	4.3	3.1	2.3	11.2	4.5	5.1	5.3	4.8	3.8	15.3
		20	5.3	5	5.1	4.1	3.2	10.3	5	4.7	4.9	4.6	3.8	12
		30	5.6	4.8	5	4	3.5	8.2	5.2	5	5	4.6	4	9.6
	TP	10	5.7	5.5	5.6	7	5.1	18	5.6	6.4	7.3	8.5	6.5	24.8
		15	5.1	5.7	5.7	6.7	5.5	13.2	4.7	5.9	6	7.2	6	17.2
		20	5.8	5.7	5.8	6.7	5.7	11	5.2	5.7	5.7	6.7	5.9	13.2
		30	6	5.2	5.2	5.7	5.1	8.9	5.7	5.2	5.2	5.8	5.3	10.3

Table 31. Simulation results for type-I error level ($\alpha = 0.05$) for normal distribution and different percentages r in case of the MCAR mechanism.

			$r = 10$					$r = 30$				
Hyp	Cov	n	WBtstrp			Alter.		WBtstrp			Alter.	
			T_W^*	T_A^*	T_M^*	A_{cl}	M_{cl}	T_W^*	T_A^*	T_M^*	A_{cl}	M_{cl}
H_0^T	AR	(5,5)	5.6	5.4	6.1	9.7	8.4	6	5.7	6.8	12.8	12.1
		(10,10)	5	5	5.3	6.9	6.9	5.3	4.9	5.5	8.4	8.6
		(10,20)	5.9	5.6	5.9	7.5	7	6.5	6.2	6.7	8.7	9
		(15,15)	4.8	4.9	4.9	6	5.9	5.2	5	5.4	7.1	7.7
		(20,20)	4.9	5.2	5.1	5.9	6.1	4.9	5	5.1	6.1	6.5
		(30,30)	5.1	5.4	5.3	5.8	6	5.8	5.6	5.8	6.7	7
	CS	(5,5)	4.9	4.6	5.6	8.9	7.7	6	5.5	7	12.9	12.1
		(10,10)	5.4	5.1	5.5	7	7.2	5.6	5.3	5.8	8.5	8.9
		(10,20)	5.8	5.5	5.9	7.2	7	6.2	5.5	6.3	8.3	8.4
		(15,15)	4.8	4.9	5	6.1	6	5.3	5.2	5.7	7.3	7.7
		(20,20)	5.4	5.3	5.6	6.3	6.5	4.8	5	5.2	6.2	6.5
		(30,30)	5	5.1	5.2	5.7	5.6	5.1	5.3	5.4	6	6.5
	TP	(5,5)	4.8	5.4	5.9	9.9	7.3	5.9	5.8	6.8	13.2	11.7
		(10,10)	4.8	5.3	5.3	7.3	6.6	5.3	5.3	5.9	8.6	8.4
		(10,20)	5.3	5.5	5.7	7.2	6.7	5.9	5.4	6	8.2	8.2
		(15,15)	4.7	5.1	5	6.4	5.9	4.8	4.9	5.2	6.8	6.8
		(20,20)	4.5	5.1	4.9	6.1	5.8	4.8	5	5.1	6.6	6.3
		(30,30)	4.8	4.8	4.8	5.3	5.4	4.9	5.3	5.4	6.2	6.2
H_0^{GT}	AR	(5,5)	6	5.2	6.2	9.3	9.4	6.4	5.4	6.6	12.7	13.4
		(10,10)	5.5	5.3	5.7	7	7	5.4	5.4	6	8.6	8.9
		(10,20)	6	5.6	5.8	7.1	6.8	6.4	6	6.6	9	9
		(15,15)	5.3	5.4	5.5	5.9	6.3	4.9	4.9	5.1	6.6	7.2
		(20,20)	5.2	5	5.2	5.9	6.4	5.2	5	5.3	6.4	6.8
		(30,30)	5.2	5	5	5.1	5.4	5.5	5.4	5.6	6.5	6.5
	CS	(5,5)	6	4.8	6.5	9.2	9.6	6.2	5.2	6.9	12.8	13.7
		(10,10)	4.8	4.6	5.1	5.8	6.1	5.4	5.1	5.9	8.2	8.8
		(10,20)	5.9	5.7	6.1	7.5	7.8	6	5.4	6.1	8.2	8.4
		(15,15)	5.1	5.1	5.2	6.5	6.2	5.2	5.2	5.6	7.2	7.7
		(20,20)	5.5	5.2	5.4	5.7	6.2	5.1	5.1	5.2	6.6	6.8
		(30,30)	5.3	5.3	5.4	5.8	5.8	4.9	5	5.1	5.9	6.1
	TP	(5,5)	5.8	5.5	6.2	9.5	9.4	6	5.7	6.7	13.5	13.2
		(10,10)	5.1	5.3	5.4	6.9	6.5	5.6	5.2	5.8	8.1	8.5
		(10,20)	5.8	5.5	5.7	6.6	6.7	5.6	5.3	5.7	7.9	7.6
		(15,15)	5.3	5	5.3	6.3	6.5	4.9	5.2	5.4	6.6	6.9
		(20,20)	4.8	5.2	5.2	6.4	6.2	5	5	5.2	6.2	6.5
		(30,30)	5	5.2	5.2	5.6	5.5	5.3	5.6	5.6	6.5	6.7

Table 32. Simulation results for type-I error level ($\alpha = 0.05$) for double exponential distribution and different percentages r in case of the MCAR mechanism.

			$r=10$					$r=30$				
Hyp	Cov	n	WBtstrp			Alter.		WBtstrp			Alter.	
			T_W^*	T_A^*	T_M^*	A_{cl}	M_{cl}	T_W^*	T_A^*	T_M^*	A_{cl}	M_{cl}
H_0^T	AR	(5,5)	5.4	5.3	6.2	9.9	8.4	5.6	5.2	6.4	12.8	11.3
		(10,10)	5	5.5	5.5	7.4	7.4	5.5	5.6	6.2	8.5	8.9
		(10,20)	5.6	5.5	5.8	7.1	6.9	6.2	5.7	6.4	8.3	8.4
		(15,15)	4.8	5.4	5.3	6.6	6.2	5.3	5.3	5.5	7.3	7.6
		(20,20)	5	5.3	5.2	6.2	6	5.4	5.3	5.6	6.8	7.4
		(30,30)	5.1	5.2	5.1	5.6	5.8	5.1	5.4	5.4	6.3	6.3
	CS	(5,5)	5.3	5.1	6	9.5	8.6	5.9	5.2	6.5	12.6	12
		(10,10)	5	5.1	5.5	7.1	7.1	5.5	5.3	5.9	8.7	9.4
		(10,20)	5.7	5.6	5.9	7.5	7.3	6.3	5.7	6.3	8.5	8.6
		(15,15)	5.1	5.2	5.2	6.3	6.5	5.4	5.3	5.7	7.1	7.6
		(20,20)	5	5.1	5.1	5.8	5.8	4.7	5	5.1	6.1	6.5
		(30,30)	4.6	4.7	4.7	5.2	5.4	4.9	5.2	5.1	5.8	6.2
	TP	(5,5)	5.1	5.2	5.6	10.3	7.5	5.9	5.9	6.6	13.4	11.7
		(10,10)	5.1	5.5	5.4	7.7	7	5.5	5.5	6	8.5	8.7
		(10,20)	5.2	5.7	5.7	7.3	6.5	5.9	5.7	6.2	8.3	8.1
		(15,15)	4.6	4.9	4.8	5.9	5.8	5	4.8	5.3	6.9	7.2
		(20,20)	5	5.1	5.1	6	6.1	5.2	5.4	5.4	6.9	6.8
		(30,30)	4.9	5.1	5	5.7	5.8	4.8	5	5	5.7	5.9
H_0^{GT}	AR	(5,5)	5.8	5.4	6.3	10.2	9.4	6.3	5.5	6.9	12.9	13.7
		(10,10)	5.1	5	5.2	7.2	7.1	5.3	5.2	5.8	8.3	8.7
		(10,20)	5.4	5.3	5.6	6.7	6.8	6.6	6	6.8	9.2	9.5
		(15,15)	5.1	5.1	5.1	6.1	5.8	5.2	5.2	5.3	7	7
		(20,20)	5.4	5.4	5.5	6.6	6.6	5.2	5.3	5.3	6.2	6.6
		(30,30)	5.6	5.4	5.6	5.7	5.8	5.2	5	5.3	5.7	6.2
	CS	(5,5)	5.6	4.9	6.2	9.9	9.5	6.3	5.5	6.9	13.3	14.1
		(10,10)	5.1	5.2	5.6	6.8	7	5.3	5.3	6	8.5	9.2
		(10,20)	5.4	5.2	5.5	6.5	6.5	6.5	6.2	6.6	8.7	9.2
		(15,15)	5	5	5.1	6.1	6.3	4.9	4.8	5	7	7.3
		(20,20)	5.1	5	5.2	5.9	6.2	5	5.1	5.3	6.3	6.7
		(30,30)	5.1	4.9	5	5.4	5.6	5	5.1	5.2	6.1	6.1
	TP	(5,5)	5.6	5	5.7	9.4	8.6	6.7	5.7	6.7	12.8	13.7
		(10,10)	5.6	5.2	5.4	7.4	7.3	5.4	5.6	6.1	8.6	9.1
		(10,20)	5.6	5	5.4	6.2	6.3	6	5.8	6.4	8.3	8.5
		(15,15)	5.4	5.6	5.5	6.6	6.5	5.2	5.1	5.4	7.2	7.9
		(20,20)	5.6	5.3	5.4	6.4	6.7	5.2	5.7	5.6	7.3	6.8
		(30,30)	5.4	5	5.2	5.7	5.9	5.1	5.2	5.2	5.9	6.3

Table 33. Simulation results for type-I error level ($\alpha = 0.05$) for chi-square distribution and different percentages r in case of the MCAR mechanism.

			$r = 10$					$r = 30$				
Hyp	Cov	n	WBtstrp			Alter.		WBtstrp			Alter.	
			T_W^*	T_A^*	T_M^*	A_{cl}	M_{cl}	T_W^*	T_A^*	T_M^*	A_{cl}	M_{cl}
H_0^T	AR	(5,5)	5.4	5.2	5.8	9.8	8	6.3	6	7	13.7	12.7
		(10,10)	5.5	5.3	5.5	7.3	7.3	5	5	5.4	8	8.2
		(10,20)	5.4	5.6	5.7	7.1	7	5.7	5.3	6	8	8.1
		(15,15)	5	4.7	5.1	6.1	6.3	5.3	5.1	5.4	7.4	7.5
		(20,20)	5	5.1	5.2	6.2	6.2	5.2	5.2	5.5	6.6	7
		(30,30)	5	5	5.1	5.5	5.7	5.4	5.4	5.5	6.4	6.6
	CS	(5,5)	5.4	4.9	5.6	9.4	8.5	6.1	5.6	6.9	13.5	12.3
		(10,10)	4.6	4.7	5	6.6	6.7	5.3	5.2	5.8	8.6	9.2
		(10,20)	5.4	5.1	5.5	6.7	7	6	5.6	6.4	8.4	8.9
		(15,15)	4.8	4.7	4.9	5.8	6	5.4	5	5.4	7.1	7.8
		(20,20)	5.3	5.3	5.3	6.1	6.4	5	5	5.3	6.4	7
		(30,30)	4.8	4.8	4.9	5.4	5.5	5.2	5.2	5.2	6	6.2
	TP	(5,5)	5.2	5.2	5.6	9.8	7.6	5.5	5.3	6.1	12.8	11.4
		(10,10)	4.7	5.2	5	7.1	6.3	5.5	5.8	6.3	9.2	9.3
		(10,20)	5.9	5.8	6	7.4	7	5.9	5.6	6.1	8	8.3
		(15,15)	4.6	4.8	4.7	6.1	5.9	5.2	5.3	5.5	7.5	7.6
		(20,20)	4.7	4.9	4.8	5.7	6	5.3	5.2	5.3	6.5	6.9
		(30,30)	4.9	5.1	5	5.7	5.6	4.7	5	5	5.9	6
H_0^{GT}	AR	(5,5)	5.9	5.4	6.5	9.9	9.7	6.3	5.5	6.8	13.2	13.3
		(10,10)	5.1	5.1	5.3	6.4	6.7	5.3	5.2	5.9	8.6	8.6
		(10,20)	5.8	5.5	5.8	7.4	7	6.2	5.7	6.4	8.2	8.4
		(15,15)	5	4.9	5	6	6.1	5.6	5.6	5.9	7.4	8.1
		(20,20)	4.8	4.8	4.9	5.4	5.6	5.2	5	5.4	6.6	6.4
		(30,30)	5.2	5.2	5.3	5.6	5.6	5.2	5	5.3	5.7	6.4
	CS	(5,5)	5.6	4.7	6.1	8.9	9.5	5.9	4.9	6.4	12.4	13.2
		(10,10)	5.1	5.2	5.5	6.8	7	5.3	5.4	6	8.4	9.3
		(10,20)	5.5	5.1	5.6	6.6	6.6	6.3	5.9	6.6	8	8.4
		(15,15)	4.7	4.7	4.8	5.6	5.7	5.4	5.1	5.7	7.2	7.7
		(20,20)	5	5	5.2	6.1	6.3	5.2	5.3	5.6	6.7	6.7
		(30,30)	5.2	5.2	5.4	5.7	5.9	5.2	5.2	5.3	5.8	6
	TP	(5,5)	5.8	5.2	6.1	9.4	8.7	6.2	5.3	6.7	12.7	12.6
		(10,10)	5.3	5.1	5.4	7.2	7.1	5.4	5.2	5.7	8.4	8.7
		(10,20)	6	5.8	6.2	7.6	7.3	6.1	5.9	6.5	8.4	8.9
		(15,15)	5.5	5.8	5.7	6.9	6.8	4.9	4.9	5.1	6.5	7
		(20,20)	5	4.9	5	6.3	6.1	4.9	5.1	5	6.2	6.2
		(30,30)	4.9	5	5	5.7	5.9	5	5.2	5.4	5.9	6.1

Table 34. Simulation results for type-I error level ($\alpha = 0.05$) for lognormal distribution and different percentages r in case of the MCAR mechanism.

Hyp	Cov	n	$r=10$ WBtstrp T_W^*	T_A^*	T_M^*	Alter. A_{cl}	M_{cl}	$r=30$ WBtstrp T_W^*	T_A^*	T_M^*	Alter. A_{cl}	M_{cl}
H_0^T	AR	(5,5)	5.5	4.8	5.8	9.3	9.8	6.1	5.5	6.5	12.9	12.4
		(10,10)	5.6	5.1	5.5	7.1	8.1	5.7	5.6	6.2	9.1	10
		(10,20)	5.7	5.2	5.5	6.9	7.8	5.7	5.2	5.7	7.9	8.5
		(15,15)	5.7	5.3	5.6	6.5	7.4	5.4	5.2	5.7	7.2	8.2
		(20,20)	5.2	4.8	5	5.5	6.7	5.3	5.3	5.4	6.7	7.4
		(30,30)	5	4.7	4.9	5.2	5.9	5.3	5.1	5.4	5.9	6.6
	CS	(5,5)	6	4.6	5.5	9.3	10.6	5.8	5.2	6.2	13	13.2
		(10,10)	5.7	5.1	5.6	6.9	8	5.6	5.1	6	8.5	9.7
		(10,20)	6.1	5.1	5.7	6.9	8	5.8	5.4	5.9	8	9
		(15,15)	5.3	5	5.3	6.2	7.2	5.2	5.2	5.5	7.2	8
		(20,20)	5	4.8	4.8	5.8	6.5	5.2	5.2	5.6	6.6	7.2
		(30,30)	5.2	5.1	5.2	5.4	6	5.3	4.9	5.2	6	6.6
	TP	(5,5)	5.3	4.8	5.3	9.6	9.5	5.7	5.3	6.2	12.7	12.3
		(10,10)	5.5	5.1	5.4	7.3	8.2	5.1	5.1	5.7	8.3	9.3
		(10,20)	6.3	5.6	6	7.2	8.4	5.9	5.3	5.8	7.9	8.9
		(15,15)	5.4	5	5.1	6.4	7.2	5.4	5.3	5.4	7.1	8
		(20,20)	5.6	4.8	5	5.6	6.9	5.8	5.1	5.5	6.4	7.7
		(30,30)	6.1	5.3	5.7	5.9	7.2	5.4	5.3	5.5	6.2	6.9
H_0^{GT}	AR	(5,5)	5.1	4.7	5.7	9.1	9.4	5.9	5.1	6.7	13	13.8
		(10,10)	5.1	5.2	5.6	6.8	7.4	5.2	5.2	5.8	8.5	8.9
		(10,20)	5.5	4.9	5.3	6.5	7.2	6.1	5.8	6.3	8.9	9.1
		(15,15)	5.2	5.2	5.4	6.4	6.6	5.3	5.1	5.7	7.3	7.8
		(20,20)	4.7	4.7	4.8	5.5	5.9	5.2	5.2	5.5	6.9	7.1
		(30,30)	4.8	4.7	4.7	5.4	5.5	5.2	5	5.3	5.9	6.1
	CS	(5,5)	5.5	4.8	6.3	9.9	10.2	5.9	5.4	6.9	13.5	14.3
		(10,10)	5.3	5.2	5.7	6.8	7.3	5.4	5.2	5.7	8.6	9.5
		(10,20)	5.6	5	5.5	6.4	6.9	6.2	5.5	6.6	8.6	8.6
		(15,15)	5.2	5.4	5.4	6.6	6.4	5.2	5	5.6	6.8	7.4
		(20,20)	5.1	5.3	5.3	6.2	6	5.3	5.2	5.6	6.6	6.7
		(30,30)	5.2	5.2	5.3	6.2	6.4	4.9	4.9	4.9	5.7	6.1
	TP	(5,5)	5.3	5.6	6.2	10.1	10.3	6	5.8	7.1	14	14.1
		(10,10)	5.1	5.1	5.4	6.9	7.2	5.9	5.7	6.2	8.9	9.5
		(10,20)	5.6	5.5	5.4	7.1	7	5.9	5.6	6.1	8.5	8.7
		(15,15)	4.9	5	5.1	5.9	5.8	5.1	5	5.3	6.9	7.3
		(20,20)	5.1	5	5	5.6	6	5.1	5	5.2	6.4	6.6
		(30,30)	5.3	5	5.2	5.7	6.2	5.1	5.3	5.5	6.5	6.5

Table 35. Simulation results for type-I error level ($\alpha = 0.05$) for normal distribution and different percentages r in case of the MCAR mechanism.

Hyp	Cov	n	$r = 10$					$r = 30$				
			WBtstrp			Alter.		WBtstrp			Alter.	
			T_W^*	T_A^*	T_M^*	A_{cl}	M_{cl}	T_W^*	T_A^*	T_M^*	A_{cl}	M_{cl}
H_0^G	AR	(5,5)	3.5	3.5	4.9	4.6	1.7	4.3	4.3	4.3	6.4	2.7
		(10,10)	4.2	4.2	4.6	3.9	2.3	4.4	4.4	4.8	5.2	3.7
		(10,20)	4.7	4.7	5.1	4.3	2.9	4.8	4.8	5	5.2	3.8
		(15,15)	5	5	5.2	4.5	3.4	4.9	4.9	4.8	4.9	3.9
		(20,20)	4.7	4.7	4.8	4.1	3.3	4.8	4.8	4.7	4.7	4
		(30,30)	4.9	4.9	4.9	4.6	4.2	5.4	5.4	5.3	5.2	4.6
	CS	(5,5)	3.5	3.5	4.8	4.4	1.3	4.2	4.2	4.6	6.8	2.9
		(10,10)	4.1	4.1	4.4	3.6	2	3.9	3.9	4.4	4.8	3.3
		(10,20)	4.6	4.6	4.9	3.9	2.7	4.8	4.8	5	5.5	3.8
		(15,15)	4.7	4.7	4.8	4.1	3.1	4.9	4.9	4.9	4.8	3.7
		(20,20)	4.8	4.8	4.9	4	3.2	5	5	4.9	4.8	4.1
		(30,30)	5.1	5.1	5.1	4.5	3.9	4.9	4.9	4.8	4.9	4.4
	TP	(5,5)	3.1	3.1	4.9	4.7	1.5	4.6	4.6	4.7	7.1	3.1
		(10,10)	4.6	4.6	5	4.1	2.6	4.6	4.6	5.1	5.5	3.9
		(10,20)	4.7	4.7	4.9	4.4	3	4.3	4.3	4.5	5.1	3.7
		(15,15)	5	5	5.2	4.2	3.1	4.5	4.5	4.4	4.7	3.8
		(20,20)	5.1	5.1	5.2	4.5	3.8	4.7	4.7	4.7	4.5	3.8
		(30,30)	4.9	4.9	5	4.7	4.1	5.2	5.2	5.1	5.1	4.6

Table 36. Simulation results for type-I error level ($\alpha = 0.05$) for double exponential distribution and different percentages r in case of the MCAR mechanism.

Hyp	Cov	n	$r=10$					$r=30$				
			WBtstrp			Alter.		WBtstrp			Alter.	
			T_W^*	T_A^*	T_M^*	A_{cl}	M_{cl}	T_W^*	T_A^*	T_M^*	A_{cl}	M_{cl}
H_0^G	AR	(5,5)	3.6	3.6	5.6	5.4	1.9	4.3	4.3	4.3	6.7	2.8
		(10,10)	4.8	4.8	5.5	5.1	3.4	4.4	4.4	4.8	5.3	3.8
		(10,20)	5.1	5.1	5.4	4.9	3.6	4.4	4.4	4.6	5.1	3.4
		(15,15)	4.8	4.8	5.1	4.8	3.9	5	5	5	5.4	4.1
		(20,20)	5	5	5.2	4.6	3.9	5	5	5	4.8	4.2
		(30,30)	5.5	5.5	5.7	5.1	4.7	5.2	5.2	5.2	5.4	4.9
	CS	(5,5)	3.6	3.6	5.1	5.4	2	4.5	4.5	4.7	7.5	3.4
		(10,10)	4.4	4.4	5	4.5	3	4.1	4.1	4.7	5.2	3.6
		(10,20)	4.8	4.8	5.2	4.6	3.2	4.4	4.4	4.9	5.2	3.8
		(15,15)	4.4	4.4	4.7	4.2	3.2	5.1	5.1	5.2	5.3	4.3
		(20,20)	4.9	4.9	5.1	4.6	3.8	5	5	5	5	4.2
		(30,30)	5.2	5.2	5.3	5	4.5	5.2	5.2	5.2	5.1	4.5
	TP	(5,5)	3.1	3.1	5	5.4	2	4.3	4.3	4.3	6.9	3.1
		(10,10)	4.2	4.2	5.1	4.8	3.2	4.3	4.3	4.6	5.1	3.5
		(10,20)	4.7	4.7	5.3	5	3.5	5.2	5.2	5.4	5.5	4
		(15,15)	5	5	5.3	4.9	3.9	4.6	4.6	4.7	4.9	4
		(20,20)	5.2	5.2	5.4	5	4.2	4.8	4.8	4.8	4.6	3.9
		(30,30)	5.3	5.3	5.4	5	4.5	5.1	5.1	5	5	4.6

Table 37. Simulation results for type-I error level ($\alpha = 0.05$) for chi-square distribution and different percentages r in case of the MCAR mechanism.

			$r=10$					$r=30$				
Hyp	Cov	n	WBtstrp			Alter.		WBtstrp			Alter.	
			T_W^*	T_A^*	T_M^*	A_{cl}	M_{cl}	T_W^*	T_A^*	T_M^*	A_{cl}	M_{cl}
H_0^G	AR	(5,5)	3.5	3.5	5.2	5.6	2.1	4.2	4.2	4.7	6.9	3.2
		(10,10)	4.5	4.5	5.3	4.7	3.1	4.6	4.6	4.8	5.4	3.8
		(10,20)	4.7	4.7	5.1	4.7	3.3	5.1	5.1	5.4	5.6	4.2
		(15,15)	4.8	4.8	5.1	4.8	3.8	4.8	4.8	4.7	4.9	4
		(20,20)	5.1	5.1	5.2	4.8	4.1	4.8	4.8	4.7	4.8	4.1
		(30,30)	5	5	5	4.8	4.3	5.1	5.1	5	4.8	4.3
	CS	(5,5)	3.9	3.9	5.6	5.4	1.9	4	4	4.3	6.5	2.8
		(10,10)	4.2	4.2	5	4.4	2.7	4.8	4.8	5.1	5.6	3.9
		(10,20)	4.7	4.7	5.1	4.4	3.1	4.3	4.3	4.6	5.2	3.6
		(15,15)	4.7	4.7	5	4.4	3.6	5	5	5	5.2	4.1
		(20,20)	4.9	4.9	5.1	4.4	3.8	4.9	4.9	4.8	4.7	3.9
		(30,30)	5	5	5	4.8	4.2	5.2	5.2	5.3	5.1	4.7
	TP	(5,5)	3.3	3.3	5	5.4	2	4.2	4.2	4.3	6.6	2.9
		(10,10)	4.4	4.4	5	4.6	3.3	4.3	4.3	4.7	5.5	3.8
		(10,20)	4.8	4.8	5.4	4.7	3.6	4.2	4.2	4.5	5	3.4
		(15,15)	4.9	4.9	5.2	4.8	3.7	4.7	4.7	4.7	4.8	3.9
		(20,20)	4.8	4.8	5.1	4.5	3.8	4.8	4.8	4.7	4.7	4.1
		(30,30)	5	5	5.1	5.1	4.5	5.3	5.3	5.3	5.2	4.6

Table 38. Simulation results for type-I error level ($\alpha = 0.05$) for lognormal distribution and different percentages r in case of the MCAR mechanism.

			$r = 10$					$r = 30$				
Hyp	Cov	n	WBtstrp			Alter.		WBtstrp			Alter.	
			T_W^*	T_A^*	T_M^*	A_{cl}	M_{cl}	T_W^*	T_A^*	T_M^*	A_{cl}	M_{cl}
H_0^G	AR	(5,5)	3.9	3.9	6.6	8.4	4.1	4.6	4.6	5.2	8.3	4.2
		(10,10)	4.8	4.8	6.1	6.1	4.5	4.8	4.8	5.5	6.2	4.6
		(10,20)	5.4	5.4	6.2	6.1	4.7	5.3	5.3	5.4	5.9	4.3
		(15,15)	4.7	4.7	5.5	5.7	4.6	4.7	4.7	5.1	5.4	4.3
		(20,20)	5	5	5.3	5.1	4.5	4.6	4.6	4.7	4.9	4
		(30,30)	5.1	5.1	5.3	5.1	4.6	4.8	4.8	4.8	4.5	4.2
	CS	(5,5)	4	4	6.8	8.6	4.1	4.7	4.7	5.2	8.6	4.3
		(10,10)	5	5	6.1	6.1	4.4	4.7	4.7	5.4	6.3	4.6
		(10,20)	5	5	5.7	5.6	4.1	4.9	4.9	5.3	6	4.3
		(15,15)	5	5	5.8	5.8	4.7	5	5	5.4	5.7	4.7
		(20,20)	4.9	4.9	5.3	5.2	4.2	5.1	5.1	5.2	5.4	4.8
		(30,30)	5.4	5.4	5.7	5.5	4.9	5.2	5.2	5.3	5.2	4.7
	TP	(5,5)	3.9	3.9	6.2	8	3.9	4.7	4.7	5.3	8.6	4.2
		(10,10)	4.2	4.2	5.4	5.5	3.9	4.4	4.4	5	5.9	4.3
		(10,20)	5.2	5.2	6	6.2	4.7	5.3	5.3	5.7	6.4	4.6
		(15,15)	5	5	5.7	5.7	4.6	4.8	4.8	5.2	5.4	4.4
		(20,20)	5.3	5.3	5.7	5.7	4.9	5	5	5.1	5.3	4.5
		(30,30)	5.2	5.2	5.4	5.3	4.8	4.7	4.7	4.8	4.9	4.5

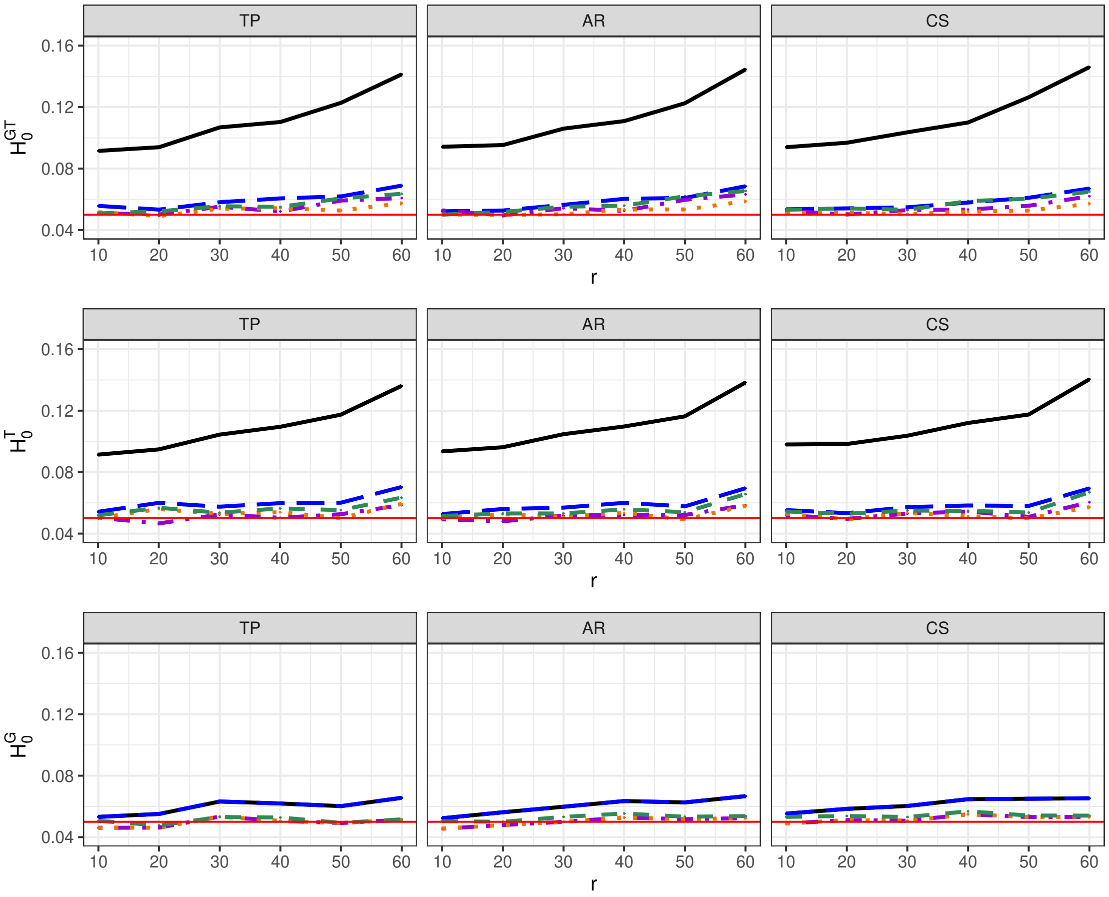

Figure 1. Type-I error simulation results ($\alpha = 0.05$) of the tests T_W (——), T_A (— —), T_W^* (–·–), T_A^* (···), and T_M^* (– - –) for double exponential distribution under different covariance structures with sample sizes $(n_1, n_2) = (15, 15)$ and $d = 4$ for varying percentages of MCAR data $r \in \{10\%, 20\%, 30\%, 40\%, 50\%, 60\%\}$.

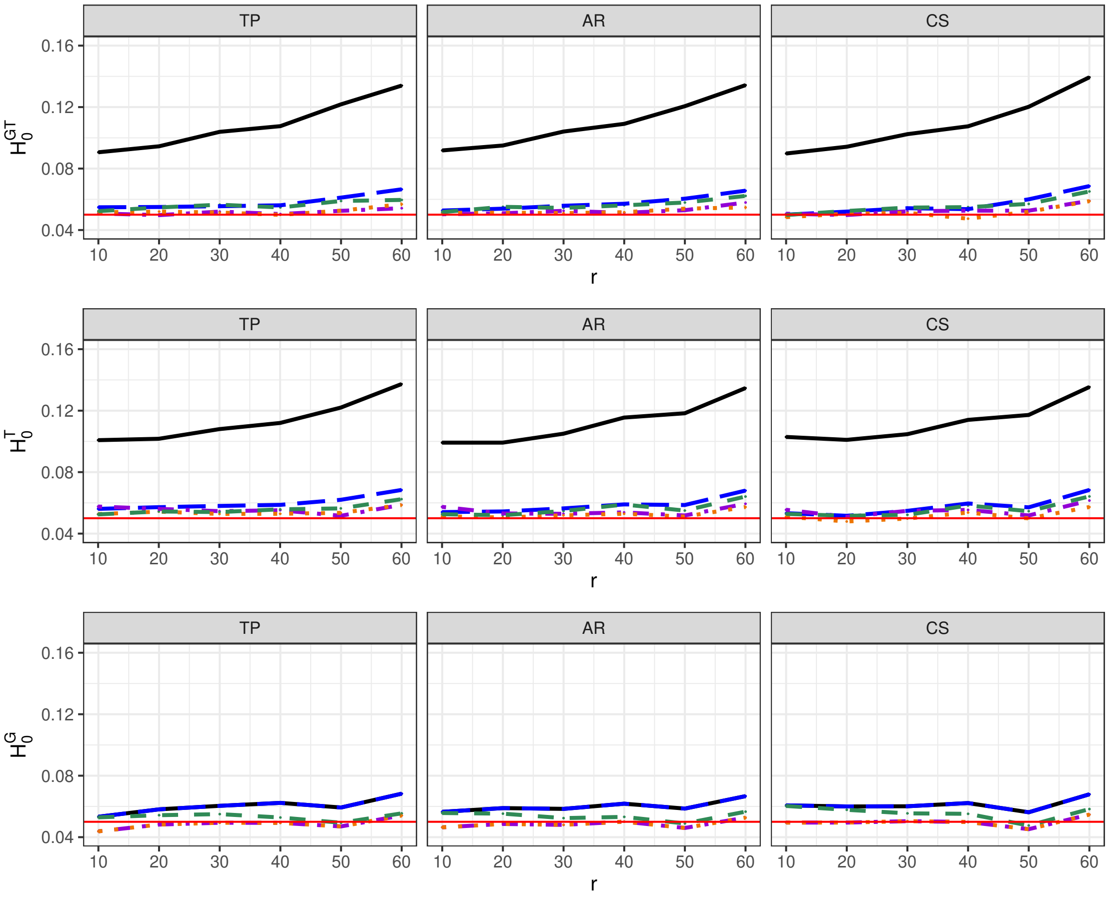

Figure 2. Type-I error simulation results ($\alpha = 0.05$) of the tests T_W (——), T_A (— —), T_W^* (– · –), T_A^* (· · ·), and T_M^* (– - –) for lognormal distribution under different covariance structures with sample sizes $(n_1, n_2) = (15, 15)$ and $d = 4$ for varying percentages of MCAR data $r \in \{10\%, 20\%, 30\%, 40\%, 50\%, 60\%\}$.

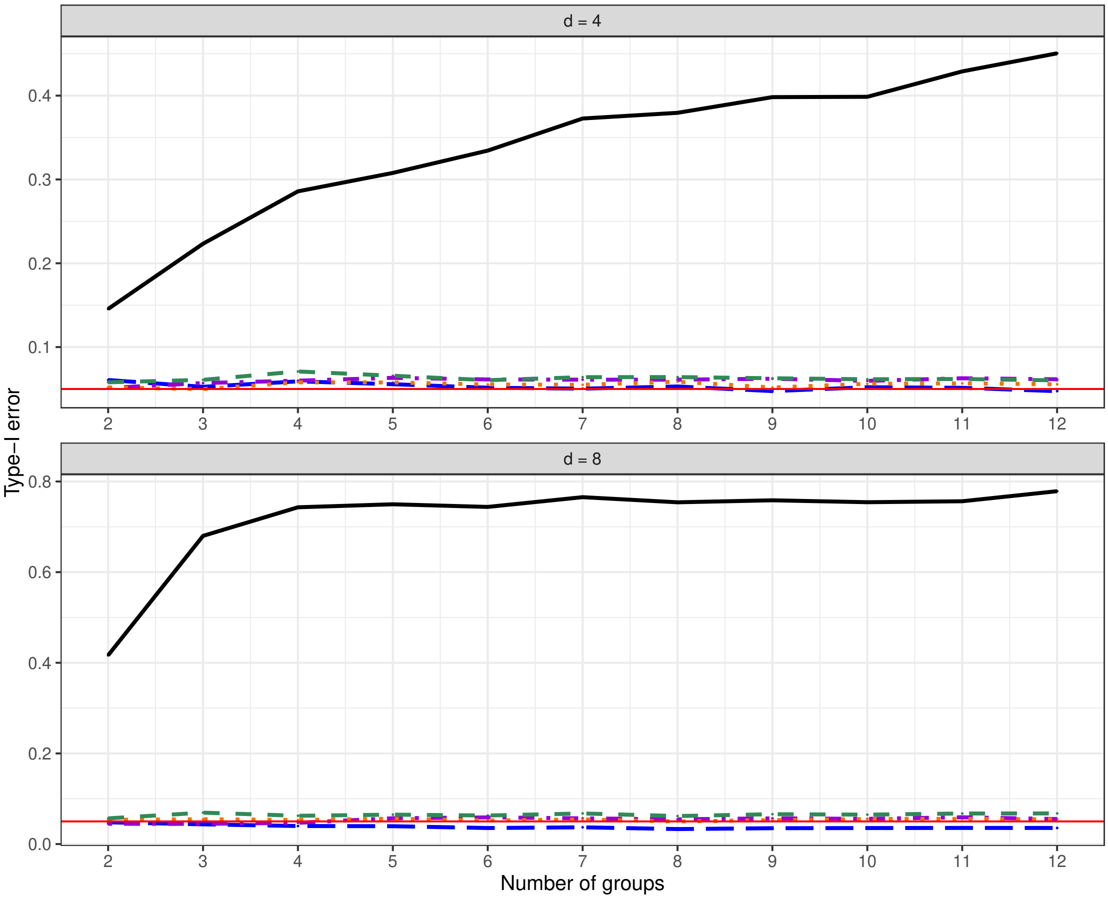

Figure 3. Type-I error simulation results ($\alpha = 0.05$) of the tests T_W (——), T_A (— —), T_W^* (− · −), T_A^* (· · ·), and T_M^* (− - −) for increasing number of groups under MCAR framework with sample sizes $n = (n_1, ..., n_{12}) = (10, 10, 15, 20, 35, 25, 15, 30, 20, 35, 20, 15)$, $d \in \{4, 8\}$ with observations generated from a lognormal distribution and under the hypothesis H_0^{GT}.

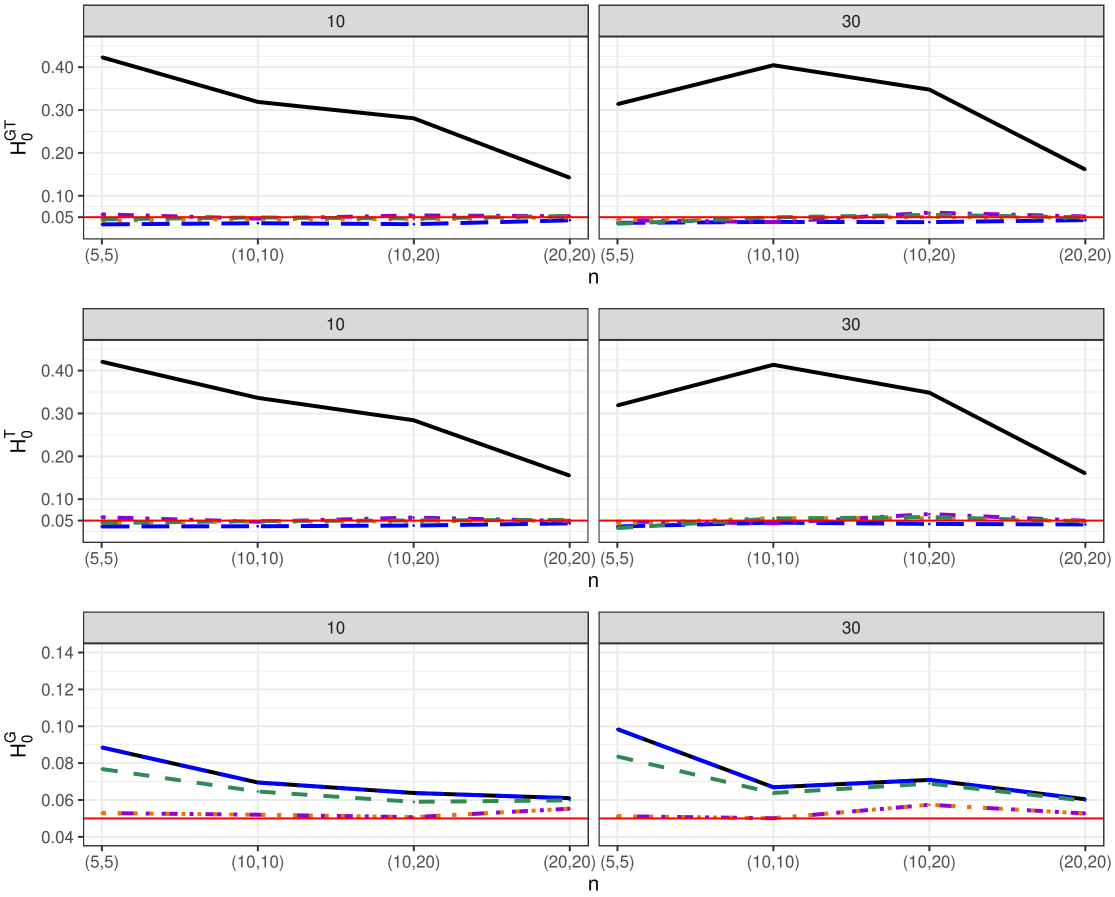

Figure 4. Type-I error simulation results ($\alpha = 0.05$) of the tests T_W (——), T_A (— —), T_W^* (–·–), T_A^* (···), and T_M^* (– – –) for ordinal data under MCAR framework with sample sizes $(n_1, n_2) = \{(5,5),(10,10),(10,20),(20,20)\}$ and $d = 8$.

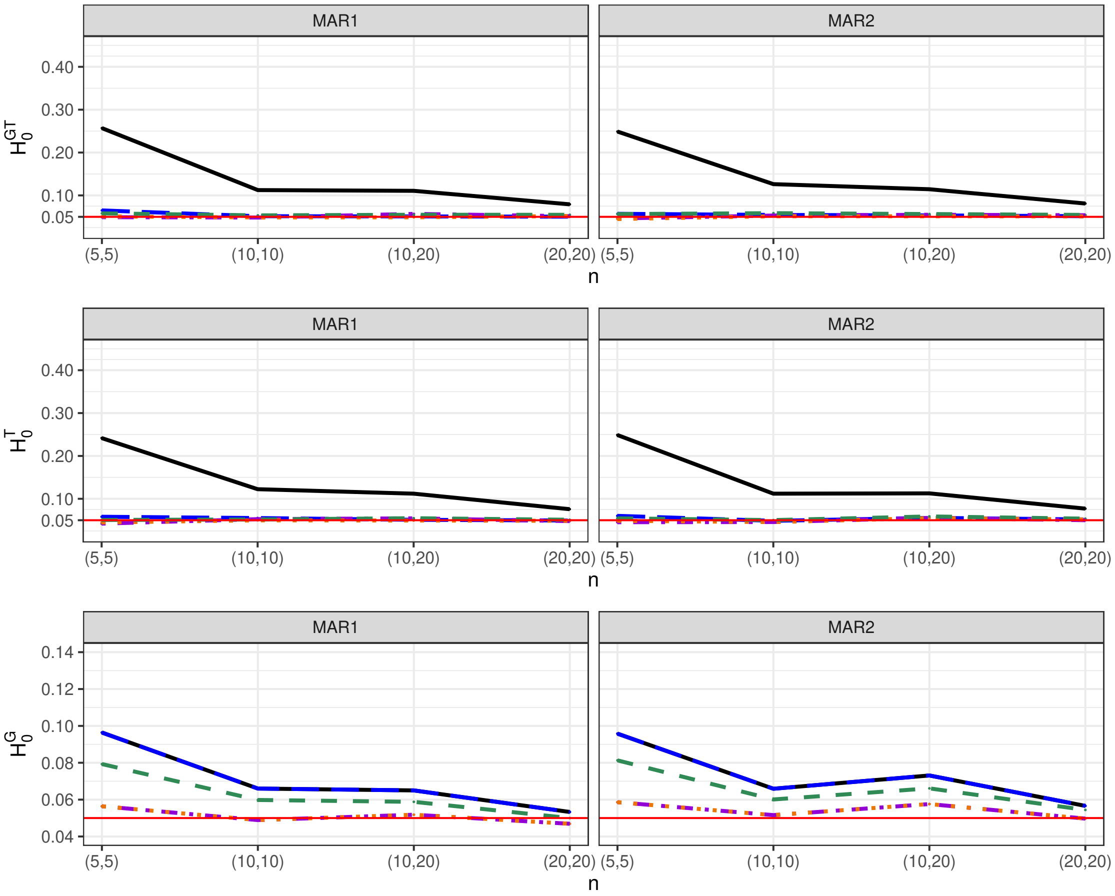

Figure 5. Type-I error simulation results ($\alpha = 0.05$) of the tests T_W (——), T_A (— —), T_W^* (–·–), T_A^* (···), and T_M^* (– – –) for ordinal data under MAR framework with sample sizes $(n_1, n_2) = \{(5,5), (10,10), (10,20), (20,20)\}$ and $d = 4$.

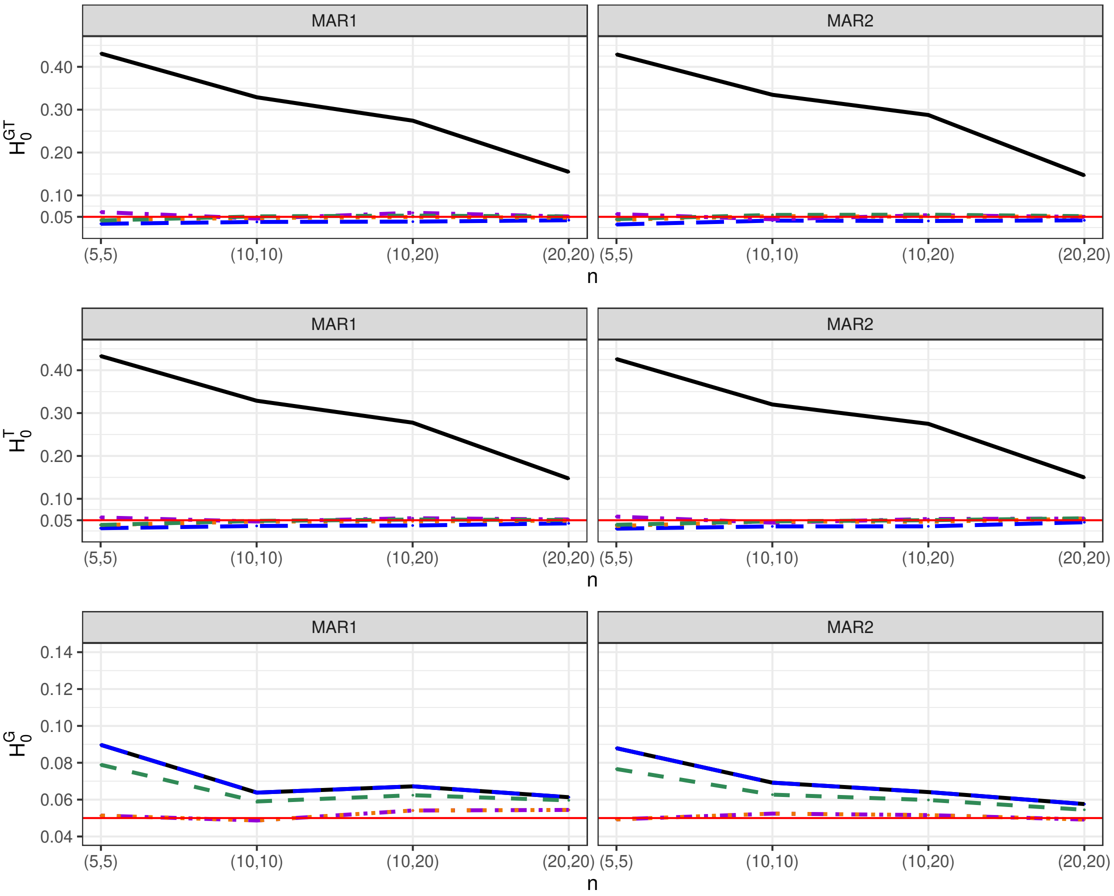

Figure 6. Type-I error simulation results ($\alpha = 0.05$) of the tests T_W (——), T_A (—— ——), T_W^* (– · –), T_A^* (· · ·), and T_M^* (– – –) for ordinal data under MAR framework with sample sizes $(n_1, n_2) = \{(5,5), (10,10), (10,20), (20,20)\}$ and $d = 8$.

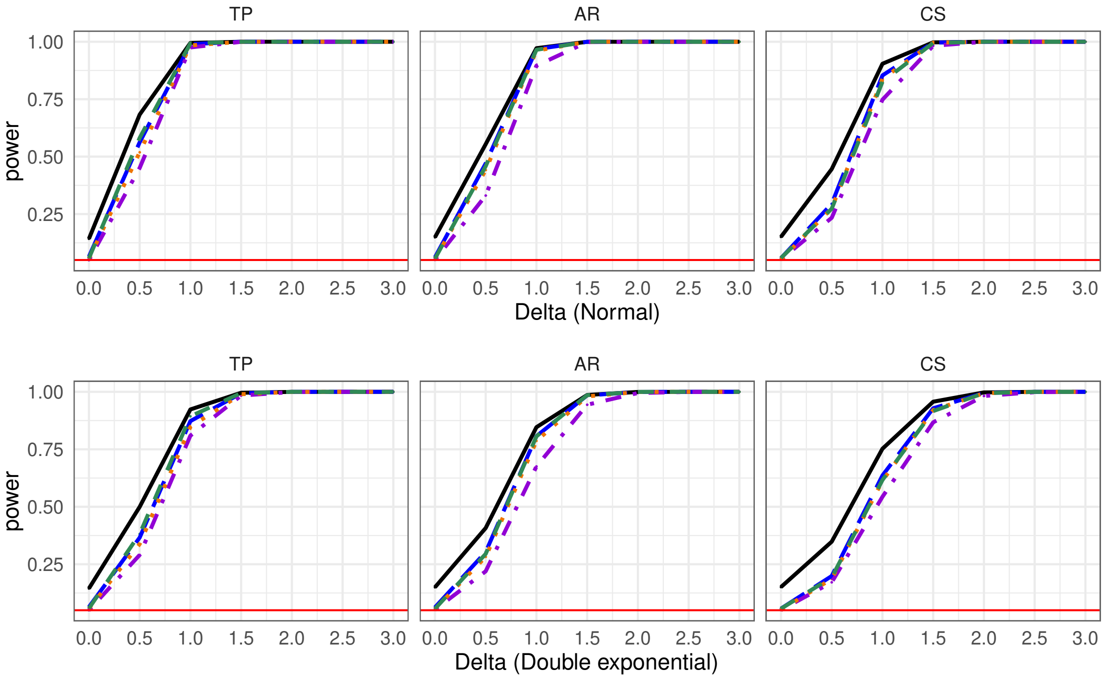

Figure 7. Power simulation results of the tests T_W (——), T_A (—— ——) , T_W^* (– · –), T_A^* (· · ·), and T_M^* (– - –) under different covariance structures with sample size $n = 15$ and $d = 4$ under alternative 1, for MCAR data with missing rate $r = 10\%$ with observations generated from a normal (upper row) and a double exponential (bottom row) distribution, respectively.

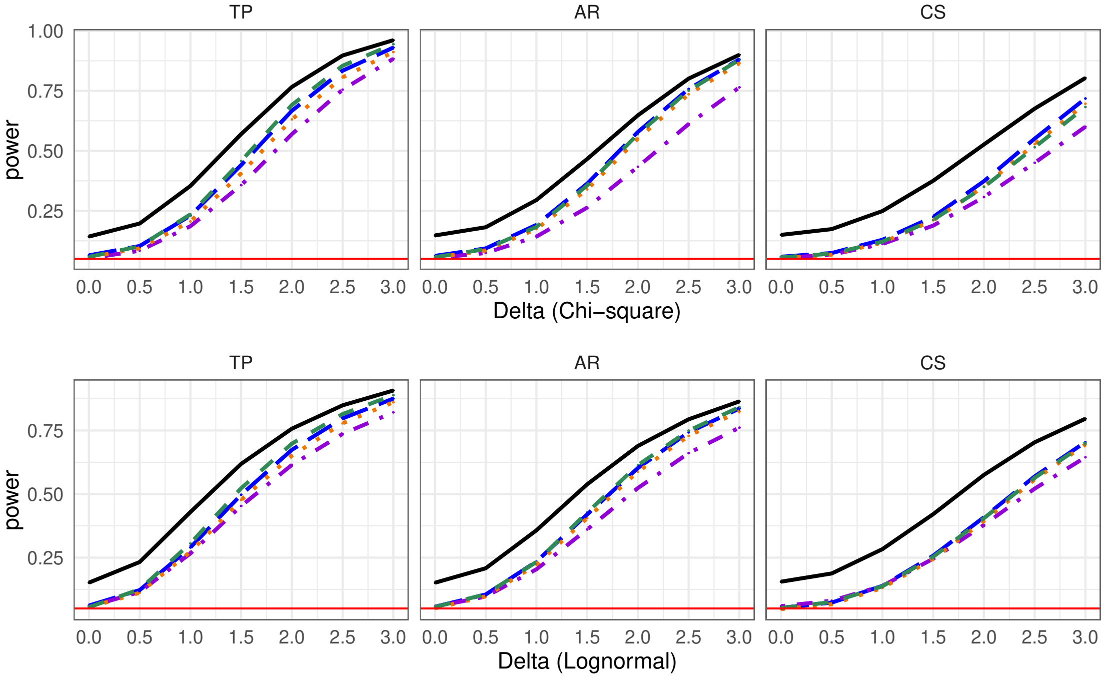

Figure 8. Power simulation results of the tests T_W (——), T_A (—— ——) , T_W^* (– · –), T_A^* (· · ·), and T_M^* (– - –) under different covariance structures with sample size $n = 15$ and $d = 4$ under alternative 1, for MCAR data with missing rate $r = 10\%$ with observations generated from a χ^2_{15} (upper row) and a lognormal (bottom row) distribution, respectively.

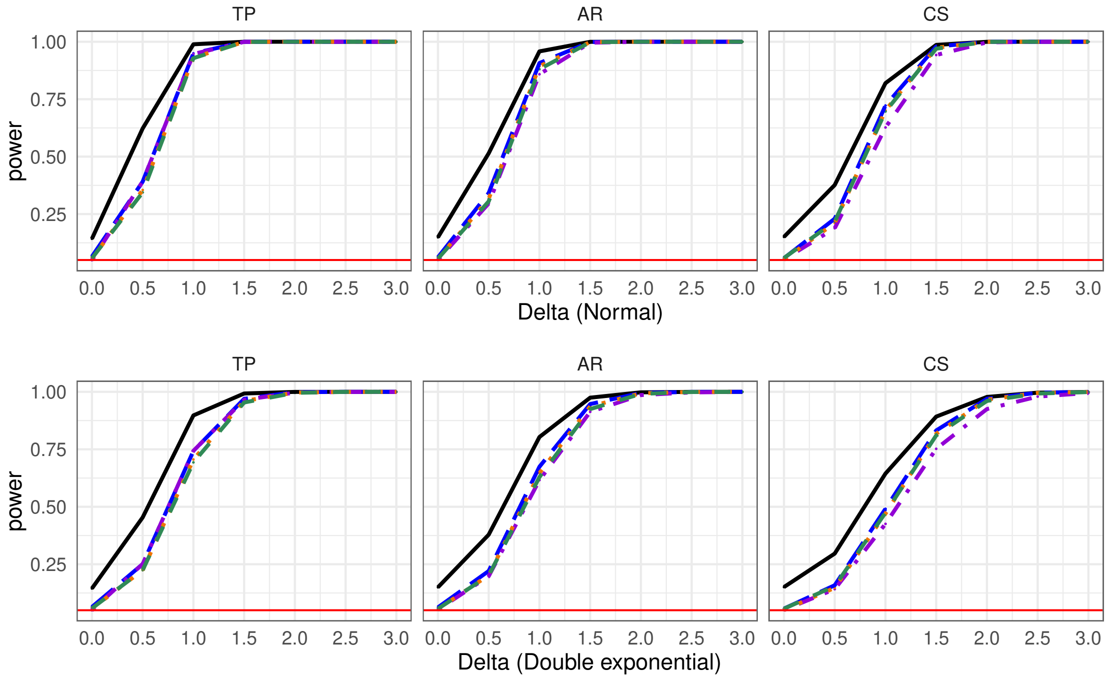

Figure 9. Power simulation results of the tests T_W (——), T_A (—— ——) , T_W^* (−·−), T_A^* (···), and T_M^* (− − −) under different covariance structures with sample size $n = 15$ and $d = 4$ under alternative 2, for MCAR data with missing rate $r = 10\%$ with observations generated from a normal (upper row) and a double exponential (bottom row) distribution, respectively.

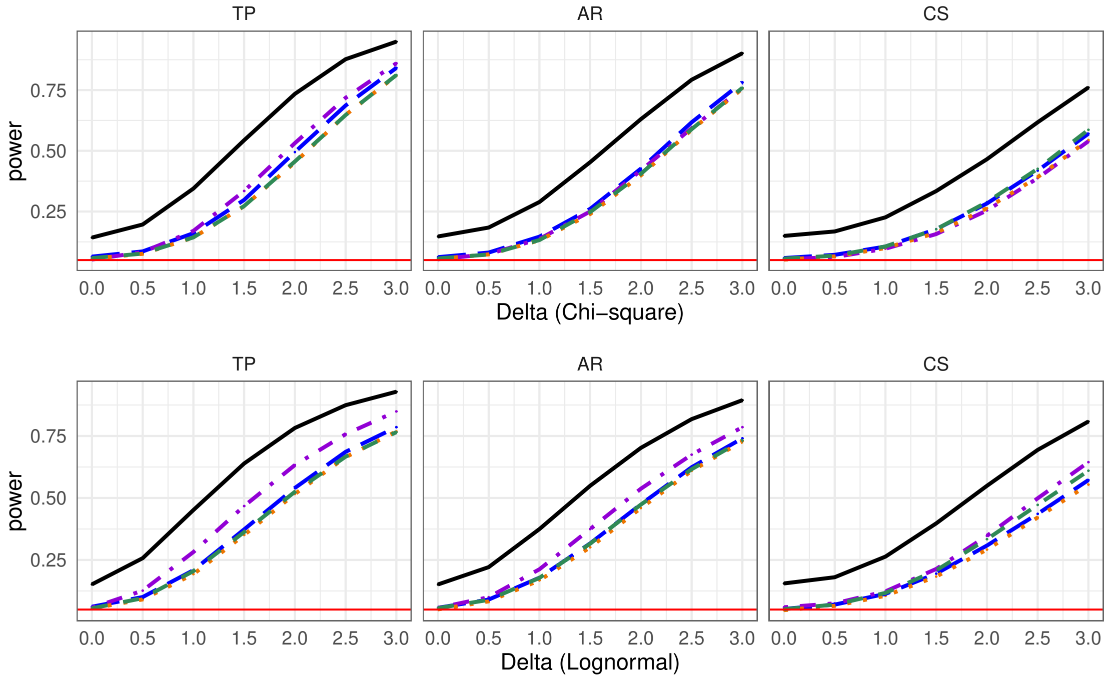

Figure 10. Power simulation results of the tests T_W (——), T_A (—— ——) , T_W^* (−·−), T_A^* (···), and T_M^* (− − −) under different covariance structures with sample size $n = 15$ and $d = 4$ under alternative 2, for MCAR data with missing rate $r = 10\%$ with observations generated from a χ^2_{15} (upper row) and a lognormal (bottom row) distribution, respectively.

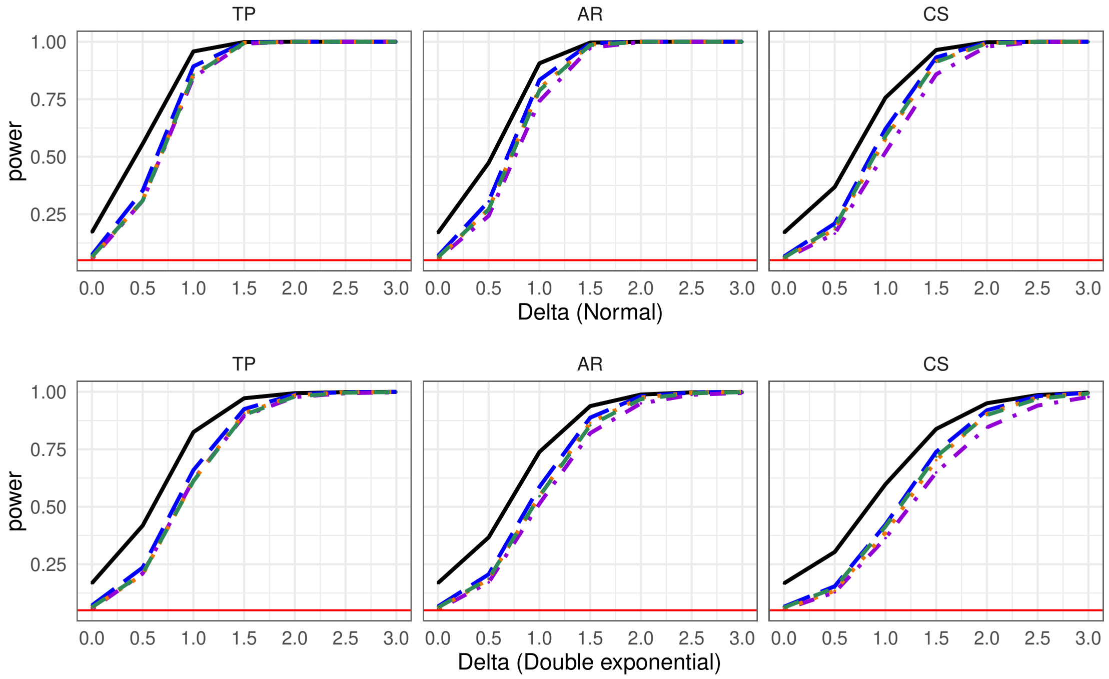

Figure 11. Power simulation results of the tests T_W (——), T_A (— —), T_W^* (– · –), T_A^* (· · ·), and T_M^* (– - –) under different covariance structures with sample size $n = 15$ and $d = 4$ under alternative 2, for MCAR data with missing rate $r = 30\%$ with observations generated from a normal (upper row) and a double exponential (bottom row) distribution, respectively.

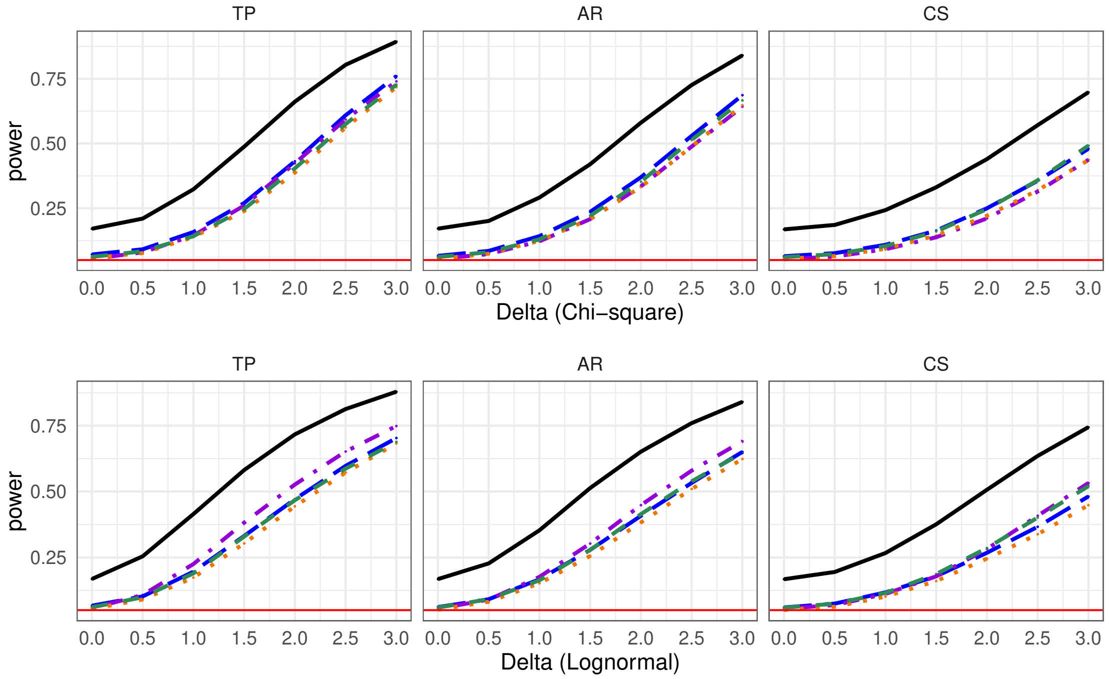

Figure 12. Power simulation results of the tests T_W (——), T_A (— —), T_W^* (– · –), T_A^* (· · ·), and T_M^* (– - –) under different covariance structures with sample size $n = 15$ and $d = 4$ under alternative 2, for MCAR data with missing rate $r = 30\%$ with observations generated from a χ^2_{15} (upper row) and a lognormal (bottom row) distribution, respectively.

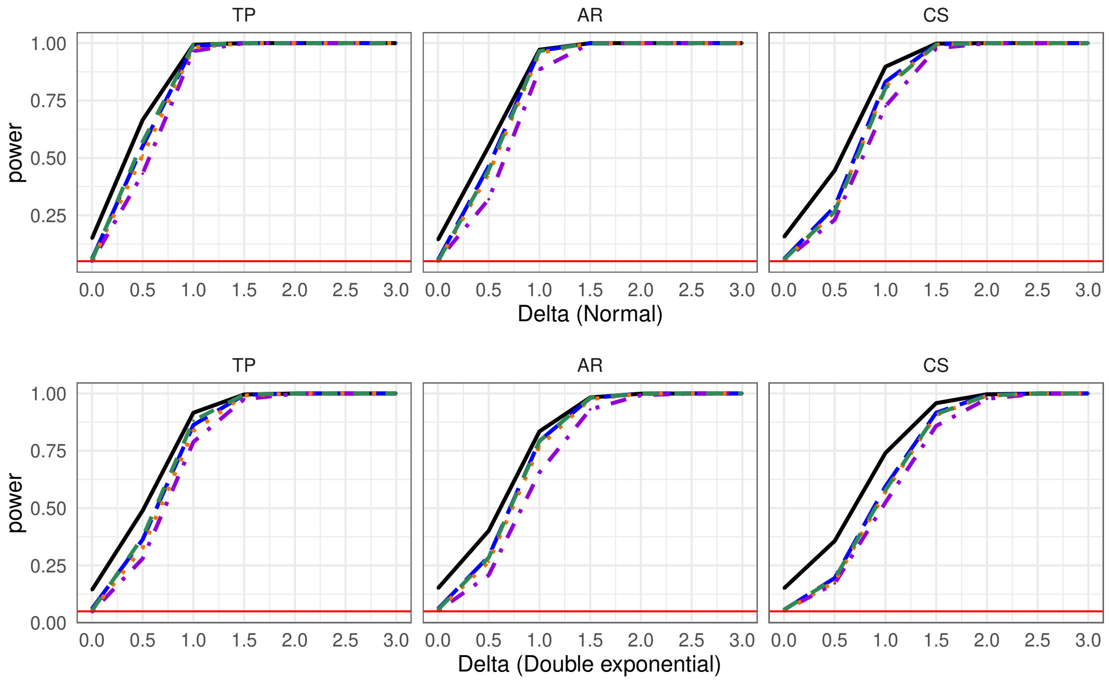

Figure 13. Power simulation results of the tests T_W (——), T_A (—— ——) , T_W^* (– · –), T_A^* (· · ·), and T_M^* (– - –) under different covariance structures with sample size $n = 15$ and $d = 4$ under alternative 1, under MAR1 with observations generated from a normal (upper row) and a double exponential (bottom row) distribution, respectively.

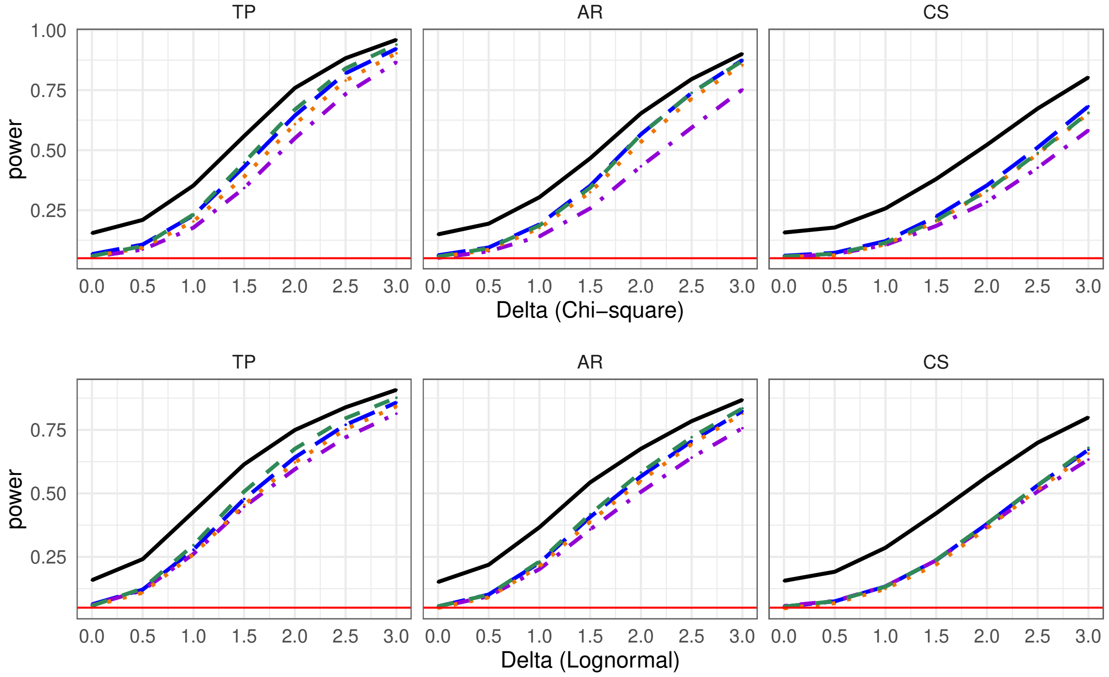

Figure 14. Power simulation results of the tests T_W (——), T_A (—— ——) , T_W^* (– · –), T_A^* (· · ·), and T_M^* (– - –) under different covariance structures with sample size $n = 15$ and $d = 4$ under alternative 1, under MAR1 with observations generated from a χ^2_{15} (upper row) and a lognormal (bottom row) distribution, respectively.

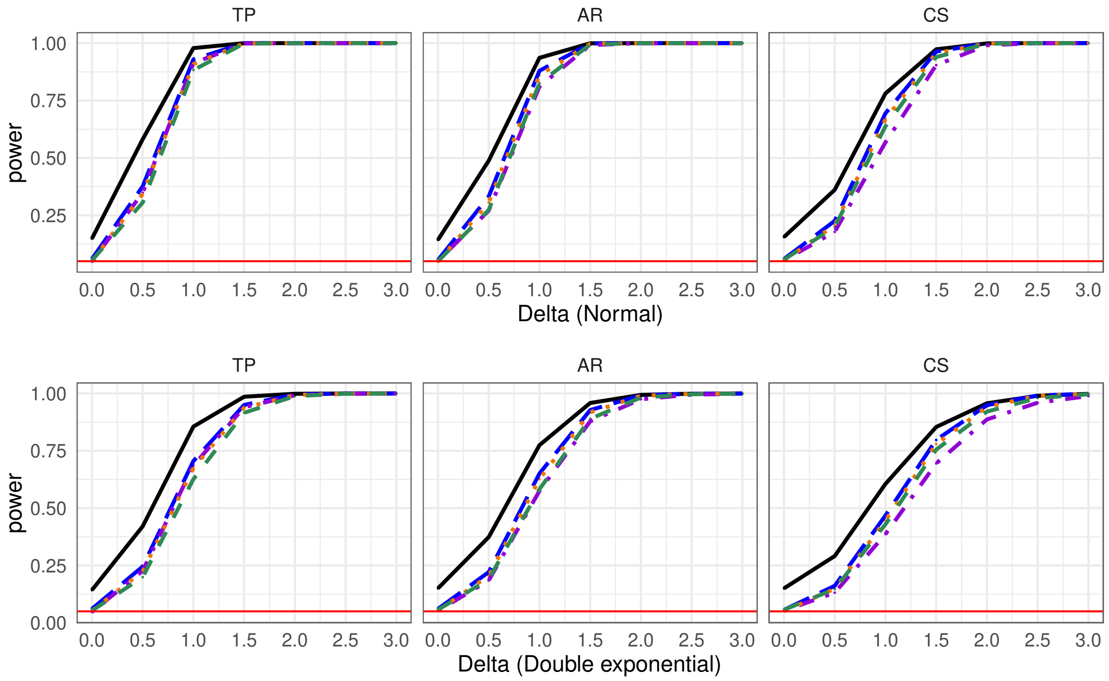

Figure 15. Power simulation results of the tests T_W (——), T_A (—— ——) , T^*_W (– · –), T^*_A (· · ·), and T^*_M (– - –) under different covariance structures with sample size $n = 15$ and $d = 4$ under alternative 2, under MAR1 with observations generated from a normal (upper row) and a double exponential (bottom row) distribution, respectively.

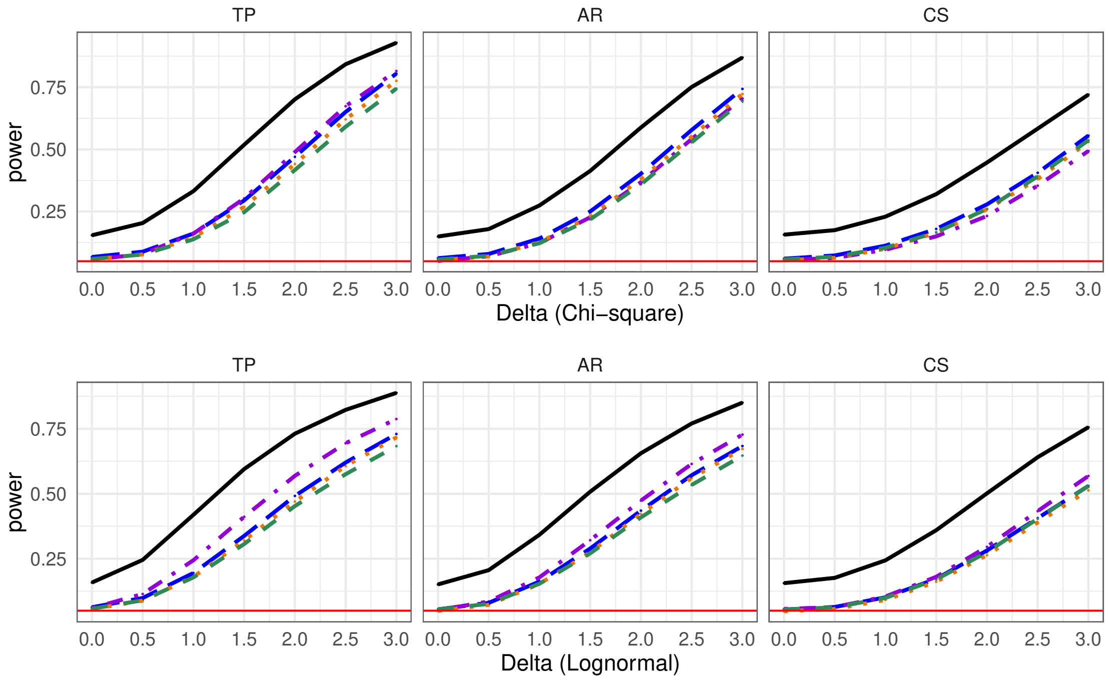

Figure 16. Power simulation results of the tests T_W (——), T_A (—— ——) , T^*_W (– · –), T^*_A (· · ·), and T^*_M (– - –) under different covariance structures with sample size $n = 15$ and $d = 4$ under alternative 2, under MAR1 with observations generated from a χ^2_{15} (upper row) and a lognormal (bottom row) distribution, respectively.

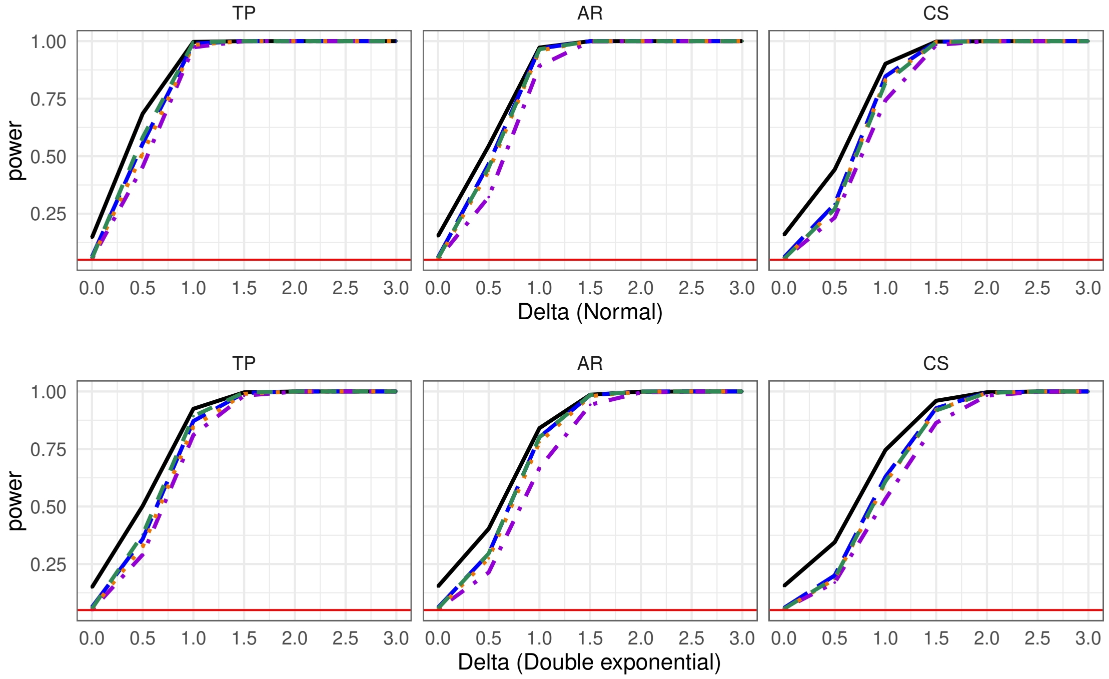

Figure 17. Power simulation results of the tests T_W (——), T_A (— —) , T_W^* (– · –), T_A^* (· · ·), and T_M^* (– - –) under different covariance structures with sample size $n = 15$ and $d = 4$ under alternative 1, under MAR2 with observations generated from a normal (upper row) and a double exponential (bottom row) distribution, respectively.

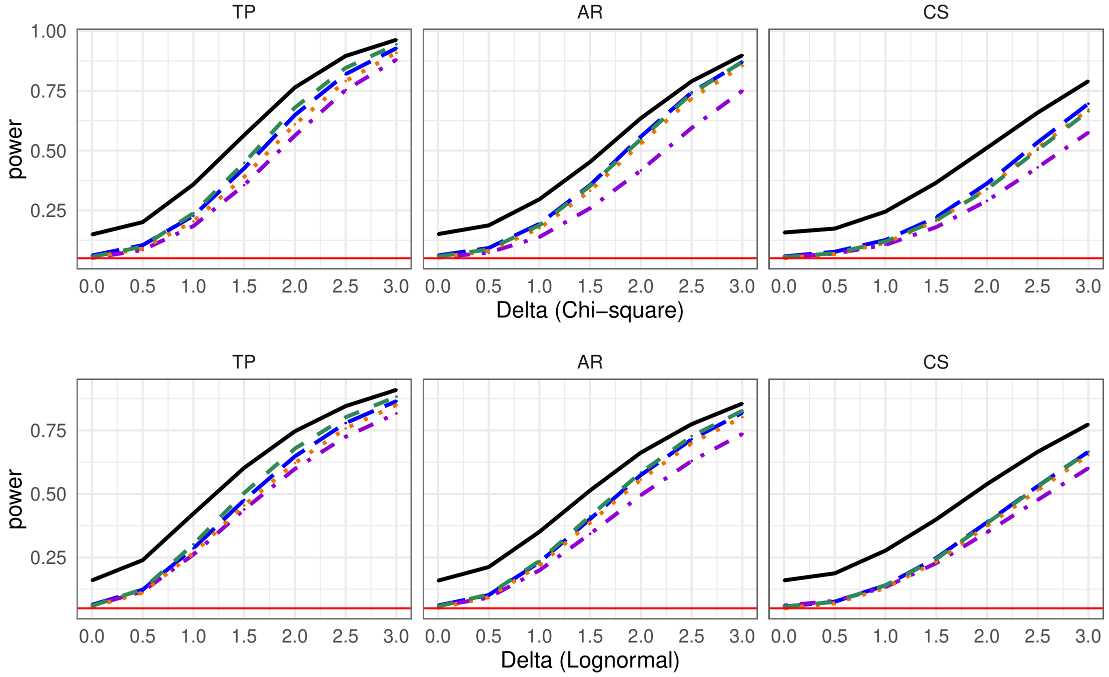

Figure 18. Power simulation results of the tests T_W (——), T_A (— —) , T_W^* (– · –), T_A^* (· · ·), and T_M^* (– - –) under different covariance structures with sample size $n = 15$ and $d = 4$ under alternative 1, under MAR2 with observations generated from a χ^2_{15} (upper row) and a lognormal (bottom row) distribution, respectively.

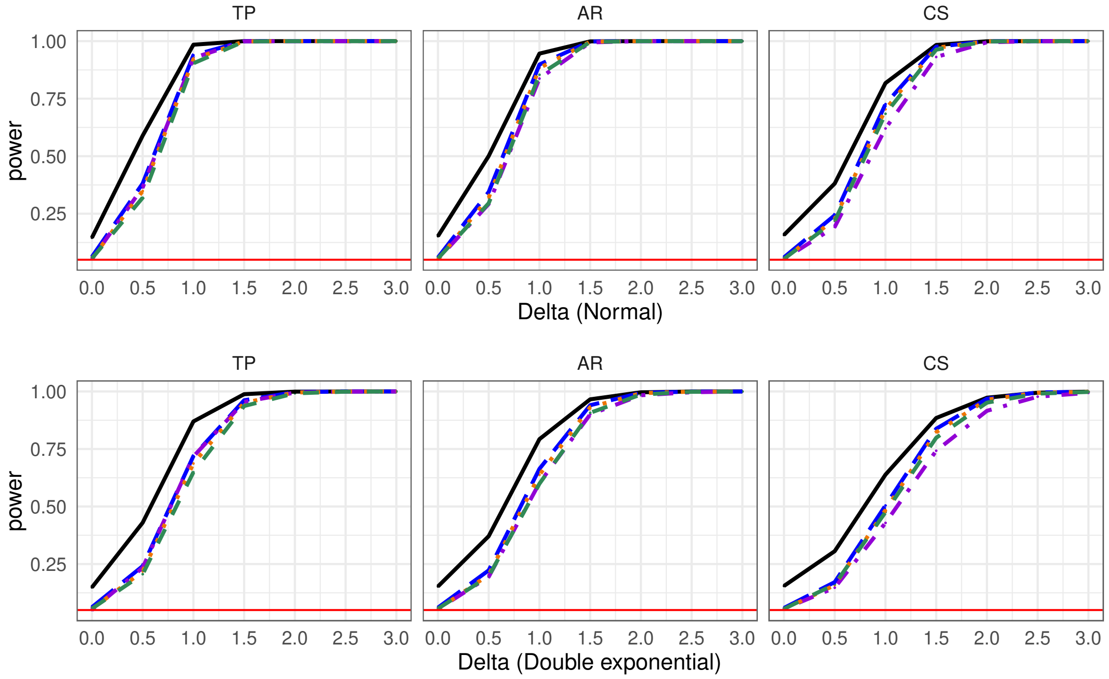

Figure 19. Power simulation results of the tests T_W (——), T_A (—— ——) , T_W^* (–·–), T_A^* (···), and T_M^* (– - –) under different covariance structures with sample size n = 15 and d = 4 under alternative 2, under MAR2 with observations generated from a normal (upper row) and a double exponential (bottom row) distribution, respectively.

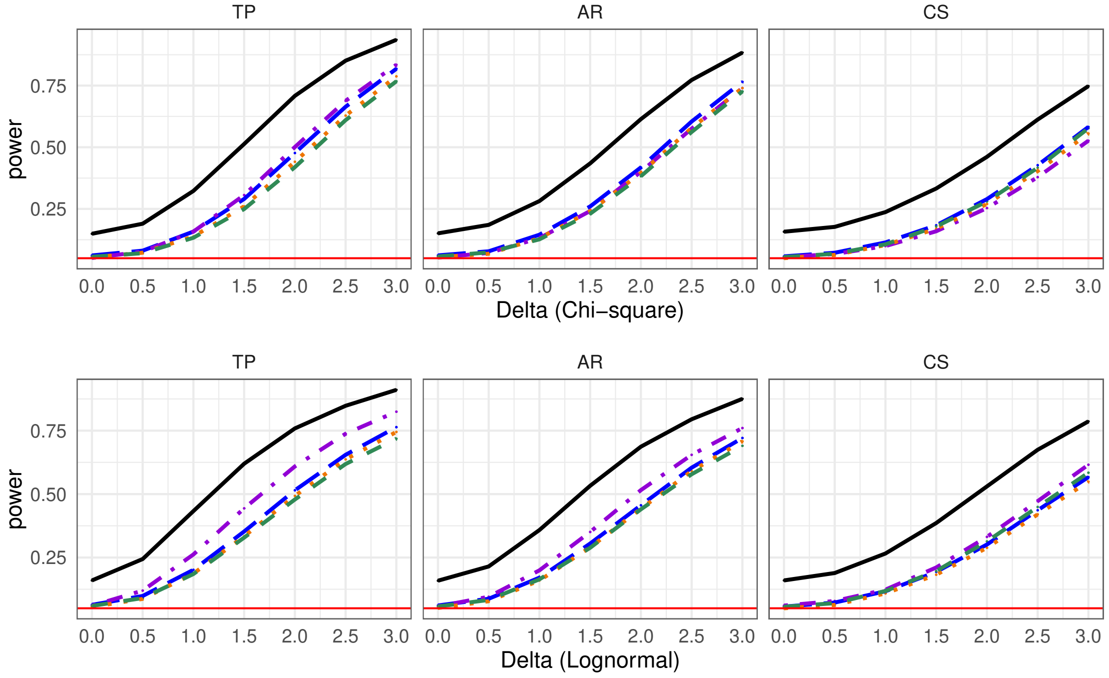

Figure 20. Power simulation results of the tests T_W (——), T_A (—— ——) , T_W^* (–·–), T_A^* (···), and T_M^* (– - –) under different covariance structures with sample size n = 15 and d = 4 under alternative 2, under MAR2 with observations generated from a χ^2_{15} (upper row) and a lognormal (bottom row) distribution, respectively.

References

Beyersmann, J., Termini, S. D., and Pauly, M. (2013). Weak convergence of the wild bootstrap for the aalen–johansen estimator of the cumulative incidence function of a competing risk. *Scandinavian Journal of Statistics*, 40(3):387–402.

Brunner, E., Munzel, U., Puri, M. L., et al. (1999). Rank-score tests in factorial designs with repeated measures. *Journal of Multivariate Analysis*, 70(2):286–317.

Brunner, E. and Puri, M. L. (2001). Nonparametric methods in factorial designs. *Statistical papers*, 42(1):1–52.

Friedrich, S. and Pauly, M. (2018). Mats: Inference for potentially singular and heteroscedastic manova. *Journal of Multivariate Analysis*, 165:166–179.

Graybill, F. A. (1976). *Theory and application of the linear model*, volume 183. Duxbury press North Scituate, MA.

Little, R. J. and Rubin, D. B. (2019). *Statistical analysis with missing data*. John Wiley & Sons.

Rao, C. R., Mitra, S. K., et al. (1972). Generalized inverse of a matrix and its applications. In *Proceedings of the Sixth Berkeley Symposium on Mathematical Statistics and Probability, Volume 1: Theory of Statistics*. The Regents of the University of California.

Rubarth, K., Pauly, M., and Konietschke, F. (2022a). Ranking procedures for repeated measures designs with missing data: Estimation, testing and asymptotic theory. *Statistical Methods in Medical Research*, 31(1):105–118.

Rubarth, K., Sattler, P., Zimmermann, H. G., and Konietschke, F. (2022b). Estimation and testing of wilcoxon–mann–whitney effects in factorial clustered data designs. *Symmetry*, 14(2).

Rubin, D. B. (1976). Inference and missing data. *Biometrika*, 63(3):581–592.
